# Supplementary material for: Highly porous metal-organic framework liquids and glasses via a solvent-assisted linker exchange strategy of ZIF-8
Source: Nat Commun. 2024 May 24;15:4420. doi: 10.1038/s41467-024-48703-5 (PMC11126584; doi:10.1038/s41467-024-48703-5)
Supplement: Supplementary file 1 — Supplementary Information [file 41467_2024_48703_MOESM1_ESM.pdf]

Supplementary Information for

Highly porous metal-organic framework liquids and glasses via a solvent-assisted linker exchange strategy of ZIF-8

Wen-Long Xue[1], Pascal Kolodzeiski[1], Hanna Kavaleuskaya[2], Suresh Vasa[2], Athanasios Koutsianos[1], Roman Pallach[1], Jianbo Song[1], Louis Frentzel-Beyme[1], Rasmus Linser[2], Sebastian Henke[1]*

[1]Anorganische Chemie, Fakultät für Chemie und Chemische Biologie, Technische Universität Dortmund, Otto-Hahn Straße 6, 44227 Dortmund, Germany. Email: sebastian.henke@tu-dortmund.de

[2]Physikalische Chemie, Fakultät für Chemie und Chemische Biologie, Technische Universität Dortmund, Otto-Hahn-Straße 4a, 44227 Dortmund, Germany.

Contents

Supplementary Methods 1 – Compositional analysis by ^{1}H NMR spectroscopy 2
Supplementary Methods 2 – X-ray powder diffraction data 13
Supplementary Methods 2.1 – Ambient temperature XRPD 13
Supplementary Methods 2.2 – Variable temperature XRPD 25
Supplementary Methods 3 – X-ray total scattering 31
Supplementary Methods 4 – Solid state 2D spin diffusion NMR spectroscopy 36
Supplementary Methods 5 – FTIR spectra 38
Supplementary Methods 6 – Thermal analysis 41
Supplementary Methods 6.1 – TGA and DSC 41
Supplementary Methods 6.2 – Heat capacity measurements 52
Supplementary Methods 6.3 – Determination of the calorimetric fragility 53
Supplementary Methods 7 – Micrography 54
Supplementary Methods 8 – Gas physisorption studies 58
Supplementary Methods 8.1 – Determination of pore volumes 58
Supplementary Methods 8.2 – Kinetic gas adsorption 62
Supplementary References 68

Supplementary Methods 1 – Compositional analysis by ^{1}H NMR spectroscopy

Solution ^{1}H NMR spectroscopy was performed on digested crystalline and glassy ZIF samples. The composition of ZIF-8 derivatives is determined using the integral ratio of the aromatic protons of the different linkers. Note that in a few spectra, there are peaks between 0 and 4 ppm, which are residual traces of *n*-butanol. This is because the materials have not been completely activated prior to the ^{1}H NMR analysis. ^{1}H NMR data of the digested parent material ZIF-8, and *n*-butanol (with DMSO-d_6 and DCl/D_2O) can be found in Supplementary Figure 3.

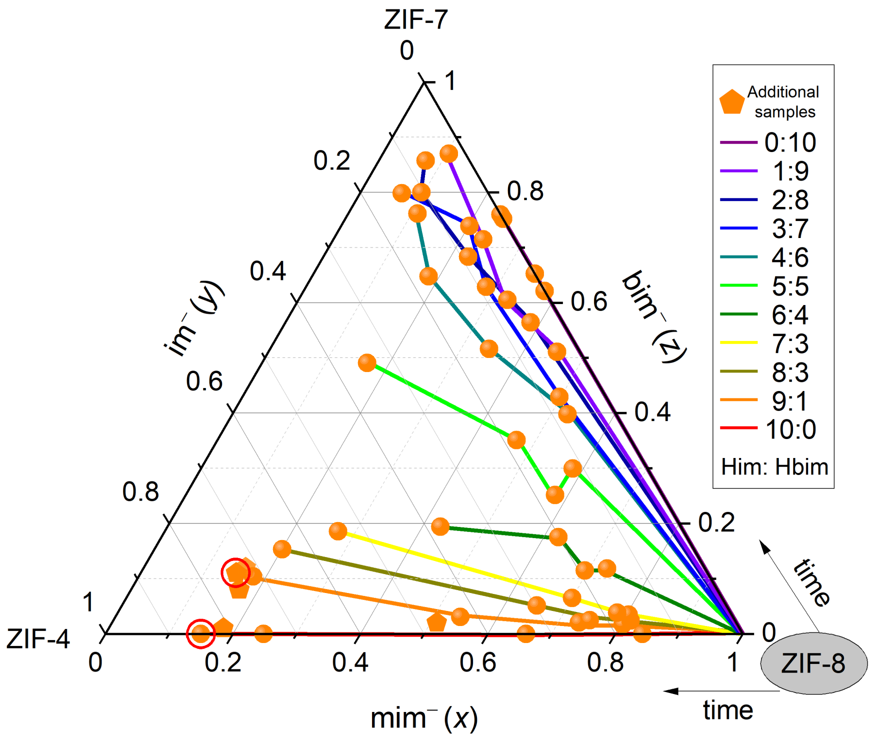

Supplementary Figure 1. Plot of the linker fractions found in the crystalline ZIF-8 derivatives after the various SALE experiments performed in this work. The orange spheres on the coloured lines show the compositions of the materials after 1 day, 3 days, 5 days and 7 days of SALE in *n*-butanol solutions containing Him and Hbim in varying ratios. The lines correspond to a SALE solution with a particular Him:Hbim ratio with the same overall molar linker concentration (i.e. c_{Him} + c_{Hbim} = constant). Lines are just a guide to the eye. The pentagons represent additional samples prepared for gathering additional data points to explore the compositional limits of meltability/glassformability in the triangular phase diagram in more detail (see Supplementary Table 1). The symbols highlighted by the red circles are the most critical samples (ZIF-8-$mim_{0.15}im_{0.74}bim_{0.11}$ and ZIF-8-$mim_{0.15}im_{0.85}$) for this work.

Supplementary Table 1. Applied masses and molar amounts of Him and Hbim with 100 mg ZIF-8 (or ZIF-67) in the SALE reaction mixtures for ZIF-8-mim$_x$im$_y$bim$_z$ (ZIF-67- mim$_x$im$_y$bim$_z$) synthesis as well as the corresponding mim$^-$: im$^-$: bim$^-$ ratios found in the corresponding framework (determined by ^{1}H NMR spectroscopy; see later spectrums). Data for an additional linker exchange experiment of ZIF-8 with Him and HClbim (5-chlorobenzimidazole) is included as well. In all exchange experiments except one, the samples have not been stirred over the course of the SALE process. In one SALE experiment, the samples suspension was stirred for 15 min/day with a total reaction time of 3 days. In this process, ZIF-8 is fully converted to a ZIF-62 derivative as proven by XRPD (see Supplementary Figure 35).

Applied Him: Hbim	Applied Him (mmol, mg)		Applied Hbim (mmol, mg)		Time (day)	Linkers fractions determined by ^{1}H NMR spectroscopy			Sample name
						mim$^-$ (%)	im$^-$ (%)	bim$^-$ (%)	
10 : 0	2.94	200	0	0	1	84	16	0	ZIF-8-mim$_{0.84}$im$_{0.16}$
					3	66	34	0	ZIF-8-mim$_{0.66}$im$_{0.34}$
					5	25	75	0	ZIF-8-mim$_{0.25}$im$_{0.75}$
					7	15	85	0	ZIF-8-mim$_{0.15}$im$_{0.85}$
9 : 1	2.64	180	0.29	34.7	1	80	18	2	ZIF-8-mim$_{0.80}$im$_{0.18}$bim$_{0.02}$
					3	73	25	2	ZIF-8-mim$_{0.73}$im$_{0.25}$bim$_{0.02}$
					5	54	43	3	ZIF-8-mim$_{0.54}$im$_{0.43}$bim$_{0.03}$
					7	18	72	10	ZIF-8-mim$_{0.18}$im$_{0.72}$bim$_{0.10}$
8 : 2	2.35	160	0.59	69.4	1	82	16	2	ZIF-8-mim$_{0.82}$im$_{0.16}$bim$_{0.02}$
					3	75	23	2	ZIF-8-mim$_{0.75}$im$_{0.23}$bim$_{0.02}$
					5	65	30	5	ZIF-8-mim$_{0.65}$im$_{0.30}$bim$_{0.05}$
					7	20	65	15	ZIF-8-mim$_{0.20}$im$_{0.65}$bim$_{0.15}$
7 : 3	2.06	140	0.88	104.1	1	80	16	4	ZIF-8-mim$_{0.80}$im$_{0.16}$bim$_{0.04}$
					3	78	18	4	ZIF-8-mim$_{0.78}$im$_{0.18}$bim$_{0.04}$
					5	70	24	6	ZIF-8-mim$_{0.70}$im$_{0.24}$bim$_{0.06}$
					7	27	54	19	ZIF-8-mim$_{0.27}$im$_{0.54}$bim$_{0.19}$
6 : 4	1.76	120	1.18	138.8	1	73	15	15	ZIF-8-mim$_{0.73}$im$_{0.15}$bim$_{0.12}$
					3	70	19	11	ZIF-8-mim$_{0.70}$im$_{0.19}$bim$_{0.11}$
					5	62	20	18	ZIF-8-mim$_{0.62}$im$_{0.20}$bim$_{0.18}$
					7	43	38	19	ZIF-8-mim$_{0.43}$im$_{0.38}$bim$_{0.19}$
5 : 5	1.47	100	1.47	173.5	1	58	12	30	ZIF-8-mim$_{0.58}$im$_{0.12}$bim$_{0.30}$
					3	58	17	25	ZIF-8-mim$_{0.58}$im$_{0.17}$bim$_{0.25}$
					5	47	18	35	ZIF-8-mim$_{0.47}$im$_{0.18}$bim$_{0.35}$
					7	17	34	49	ZIF-8-mim$_{0.17}$im$_{0.34}$bim$_{0.49}$
4 : 6	1.18	80	1.76	208.2	1	53	7	40	ZIF-8-mim$_{0.53}$im$_{0.07}$bim$_{0.40}$
					3	34	14	52	ZIF-8-mim$_{0.34}$im$_{0.14}$bim$_{0.52}$
					5	18	17	65	ZIF-8-mim$_{0.18}$im$_{0.17}$bim$_{0.65}$
					7	11	13	76	ZIF-8-mim$_{0.11}$im$_{0.13}$bim$_{0.76}$
3 : 7	0.88	60	2.06	242.9	1	50	7	43	ZIF-8-mim$_{0.50}$im$_{0.07}$bim$_{0.43}$
					3	28	9	63	ZIF-8-mim$_{0.28}$im$_{0.09}$bim$_{0.63}$
					5	20	6	74	ZIF-8-mim$_{0.20}$im$_{0.06}$bim$_{0.74}$
					7	7	13	80	ZIF-8-mim$_{0.07}$im$_{0.13}$bim$_{0.80}$
2 : 8	0.59	40	2.35	277.6	1	39	5	56	ZIF-8-mim$_{0.39}$im$_{0.05}$bim$_{0.56}$
					3	23	9	68	ZIF-8-mim$_{0.23}$im$_{0.09}$bim$_{0.68}$
					5	10	10	80	ZIF-8-mim$_{0.10}$im$_{0.10}$bim$_{0.80}$
					7	7	7	86	ZIF-8-mim$_{0.07}$im$_{0.07}$bim$_{0.86}$
1 : 9	0.29	20	2.64	312.4	1	45	4	51	ZIF-8-mim$_{0.45}$im$_{0.04}$bim$_{0.51}$
					3	33	7	60	ZIF-8-mim$_{0.33}$im$_{0.07}$bim$_{0.60}$
					5	24	5	71	ZIF-8-mim$_{0.24}$im$_{0.05}$bim$_{0.71}$
					7	10	3	87	ZIF-8-mim$_{0.10}$im$_{0.03}$bim$_{0.87}$
0 : 10	0	0	2.94	347.1	1	38	0	62	ZIF-8-mim$_{0.38}$bim$_{0.62}$
					3	35	0	65	ZIF-8-mim$_{0.35}$bim$_{0.65}$
					5	25	0	75	ZIF-8-mim$_{0.25}$bim$_{0.75}$
					7	24	0	76	ZIF-8-mim$_{0.24}$bim$_{0.76}$
8.7 : 1.3	2.35	160	0.34	40	7	15	74	11	ZIF-8-mim$_{0.15}$im$_{0.74}$bim$_{0.11}$
9.3 : 0.7	2.60	177	0.19	23	7	17	75	8	ZIF-8-mim$_{0.17}$im$_{0.75}$bim$_{0.08}$
8.8 : 1.2	2.57	175	0.36	43	7	16	72	12	ZIF-8-mim$_{0.16}$im$_{0.72}$bim$_{0.12}$
9.7 : 0.3	2.79	190	0.08	10	7	18	81	1	ZIF-8-mim$_{0.18}$im$_{0.81}$bim$_{0.01}$
9.3 : 0.7	2.60	177	0.19	23	3.5	51	47	2	ZIF-8-mim$_{0.51}$im$_{0.47}$bim$_{0.02}$
8.7 : 1.3	2.35	160	0.34	40	3 (stirred 15 min/day)	5	80	15	ZIF-62-mim$_{0.05}$im$_{0.80}$bim$_{0.15}$
8.7 : 1.3 (ZIF-67)	2.35	160	0.34	40	7	18	68	14	ZIF-67-mim$_{0.18}$im$_{0.68}$bim$_{0.14}$
8.7 : 1.3 (Him: HClbim)	2.35	160	0.34	52	7	20	70	10	ZIF-8-mim$_{0.20}$im$_{0.70}$Clbim$_{0.10}$

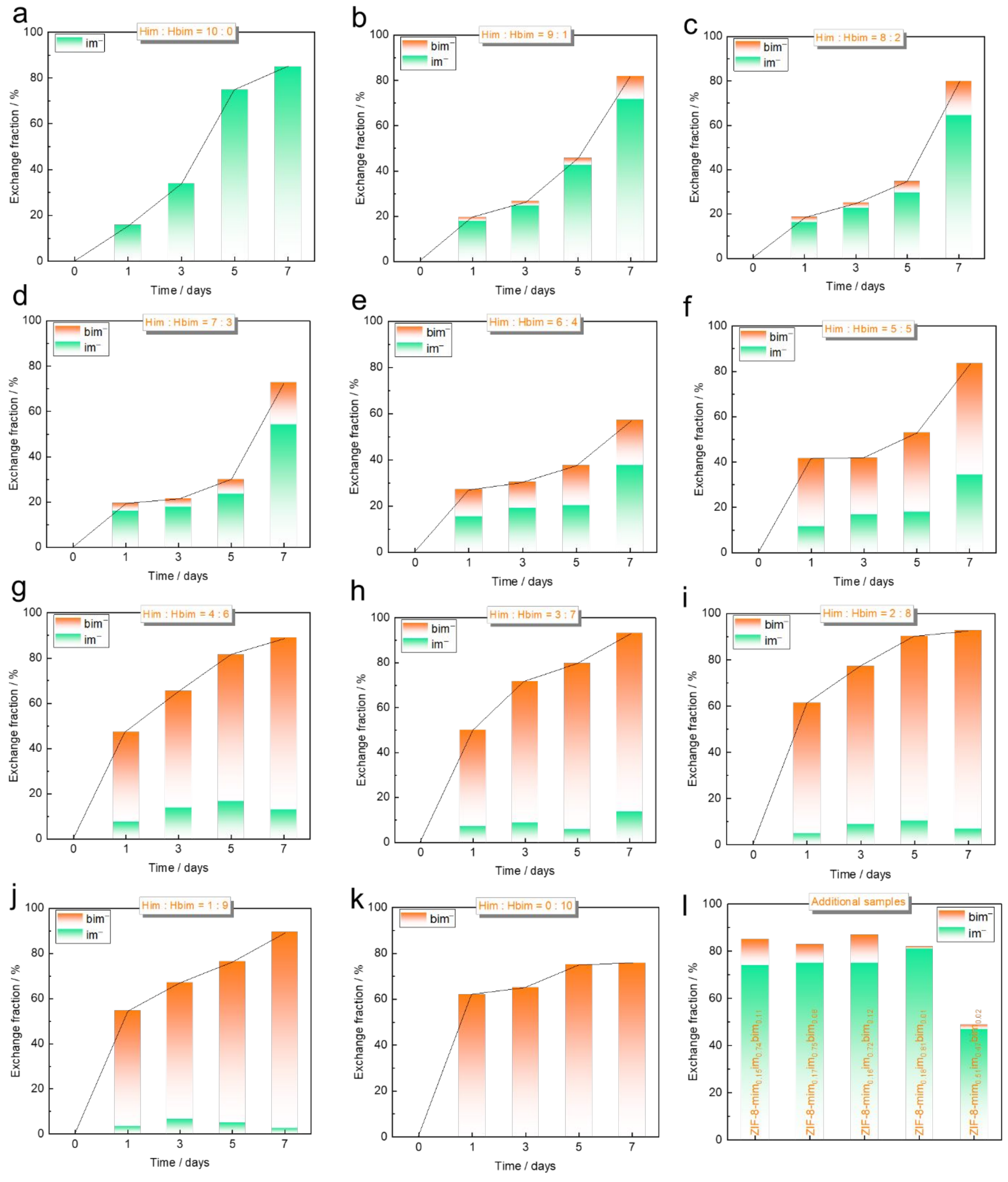

Supplementary Figure 2. Progress of the SALE reactions expressed as the fraction of im^- and bim^- in ZIF-8. The fraction of mim^- can be obtained by subtracting the fractions of im^- and bim^- from 100%. As to be expected, the fraction of exchanged linkers (i.e. the inclusion of im^- and/or bim^- and the removal of mim^-) is increasing with the reaction time. Moreover, bim^- is incorporated preferentially over im^- so that the rate for the incorporation of im^- is much slower than the rate for the incorporation of bim^-. These observations seem counterintuitive at first sight, as Hbim is significantly larger than Him and thus a considerable restriction of diffusion into the pores of ZIF-8 is expected for the former. However, the observations are consistent with the considerably higher acidic strength of Hbim (pK_a = 12.78) compared to Him (pK_a = 14.52)[1].

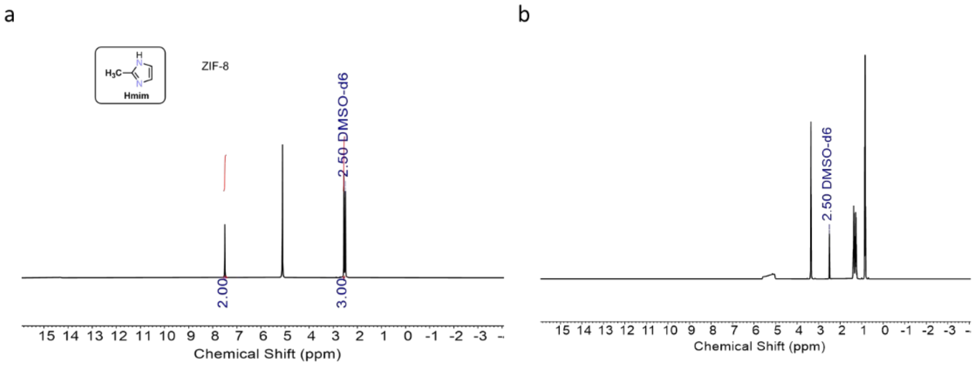

Supplementary Figure 3. ^{1}H NMR data collected for **a** ZIF-8 and **b** *n*-butanol using DMSO-d_6 (0.5 mL) and DCl/D_2O (35 wt%, 0.015 mL) as solvents.

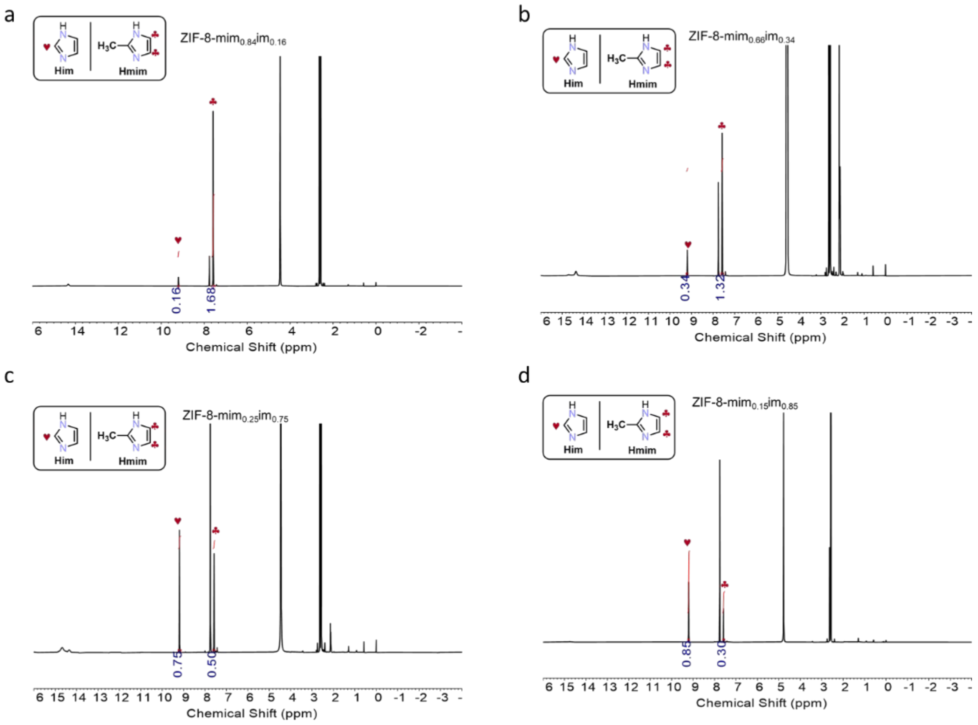

Supplementary Figure 4. ^{1}H NMR data collected for ZIF-8 derivatives after varying SALE reaction times with a solution containing only Him. The SALE time from **a** to **d** is 1, 3, 5, and 7 days, respectively.

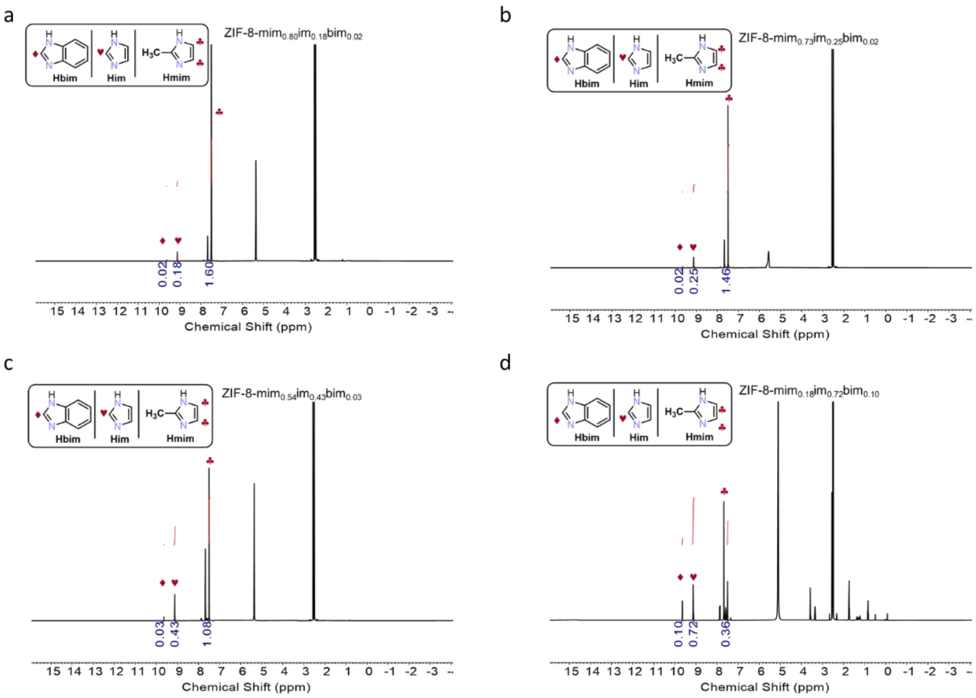

Supplementary Figure 5. ^{1}H NMR data collected for ZIF-8 derivatives after varying SALE reaction times with a solution containing Him and Hbim in a molar ratio of 9:1. The SALE time from **a** to **d** is 1, 3, 5, and 7 days, respectively.

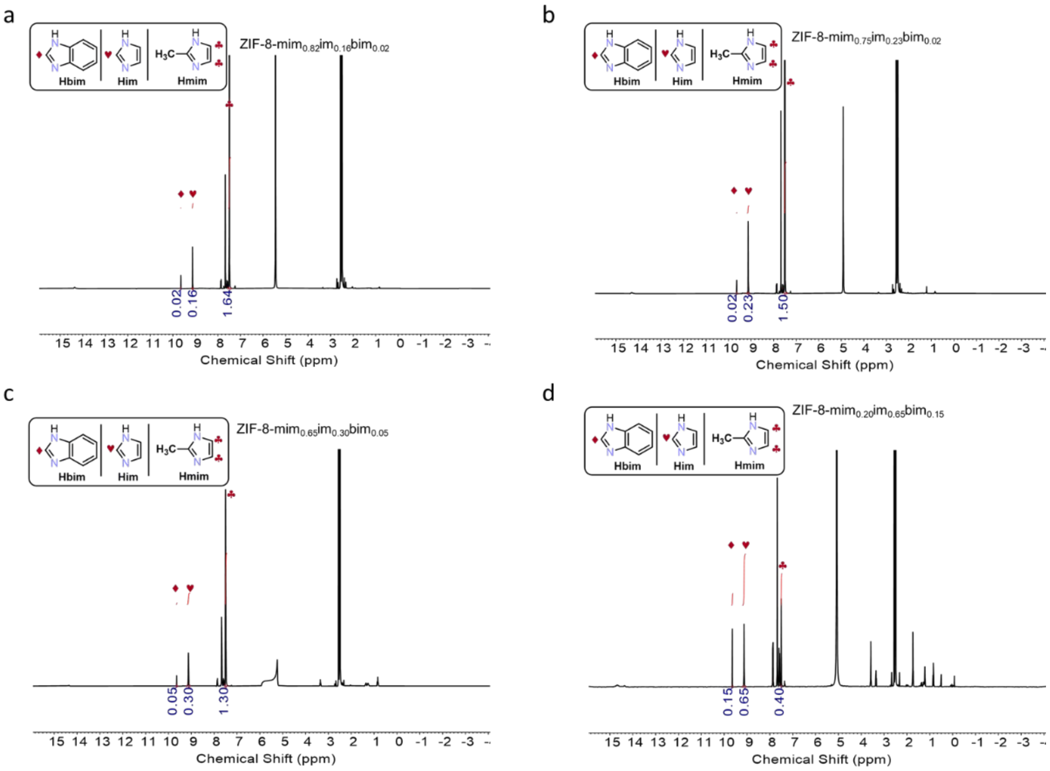

Supplementary Figure 6. ^{1}H NMR data collected for ZIF-8 derivatives after varying SALE reaction times with a solution containing Him and Hbim in a molar ratio of 8:2. The SALE time from **a** to **d** is 1, 3, 5, and 7 days, respectively.

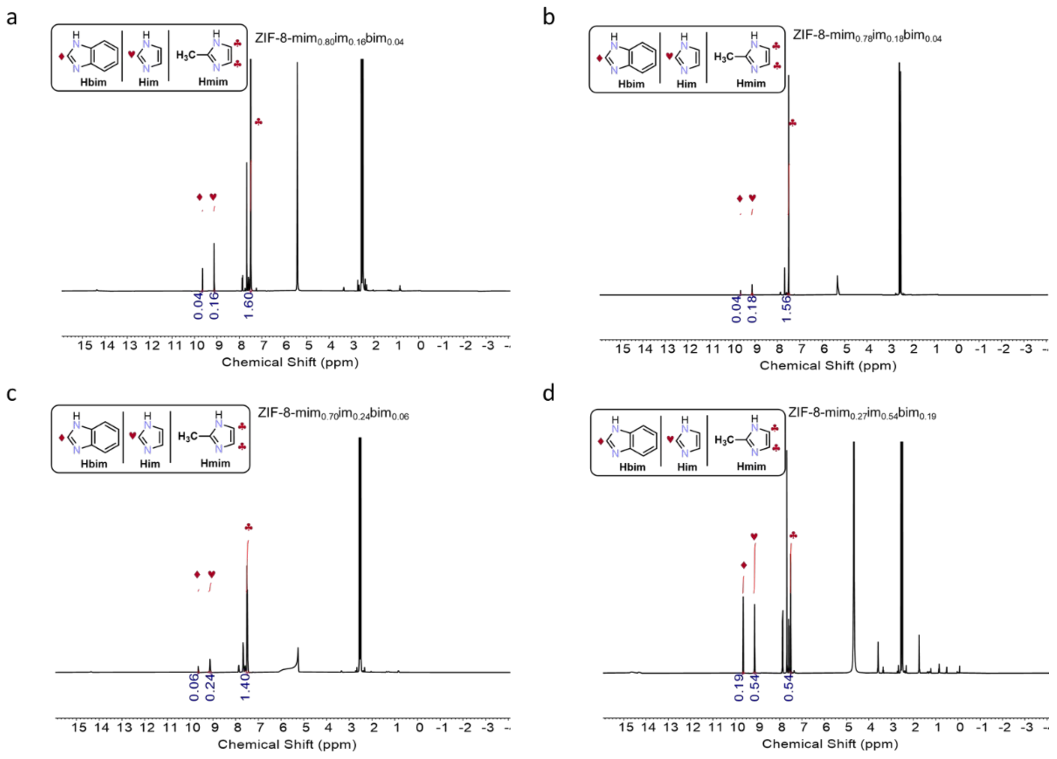

Supplementary Figure 7. ^{1}H NMR data collected for ZIF-8 derivatives after varying SALE reaction times with a solution containing Him and Hbim in a molar ratio of 7:3. The SALE time from **a** to **d** is 1, 3, 5, and 7 days, respectively.

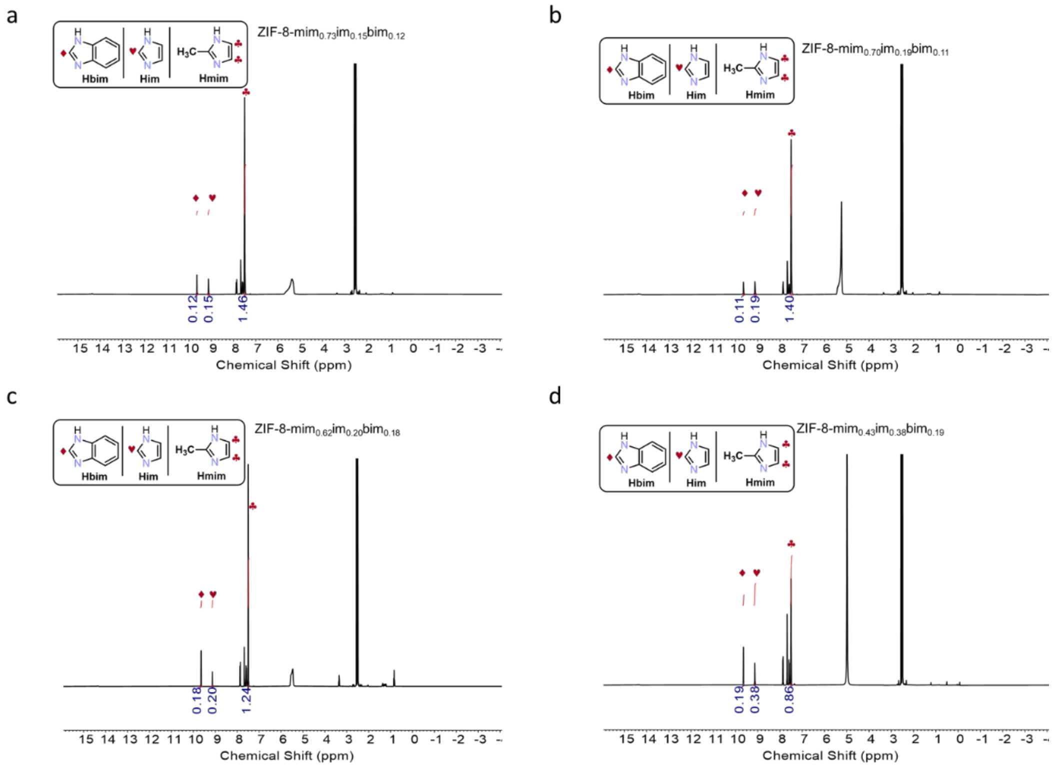

Supplementary Figure 8. ^{1}H NMR data collected for ZIF-8 derivatives after varying SALE reaction times with a solution containing Him and Hbim in a molar ratio of 6:4. The SALE time from **a** to **d** is 1, 3, 5, and 7 days, respectively.

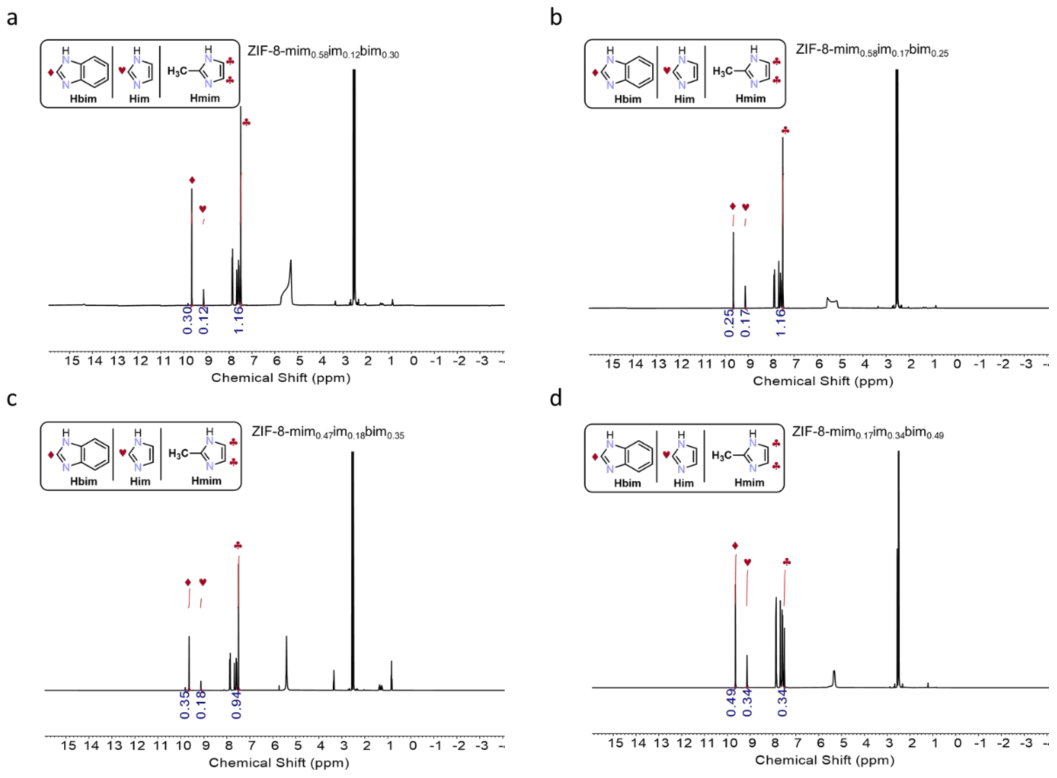

Supplementary Figure 9. ^{1}H NMR data collected for ZIF-8 derivatives after varying SALE reaction times with a solution containing Him and Hbim in a molar ratio of 5:5. The SALE time from **a** to **d** is 1, 3, 5, and 7 days, respectively.

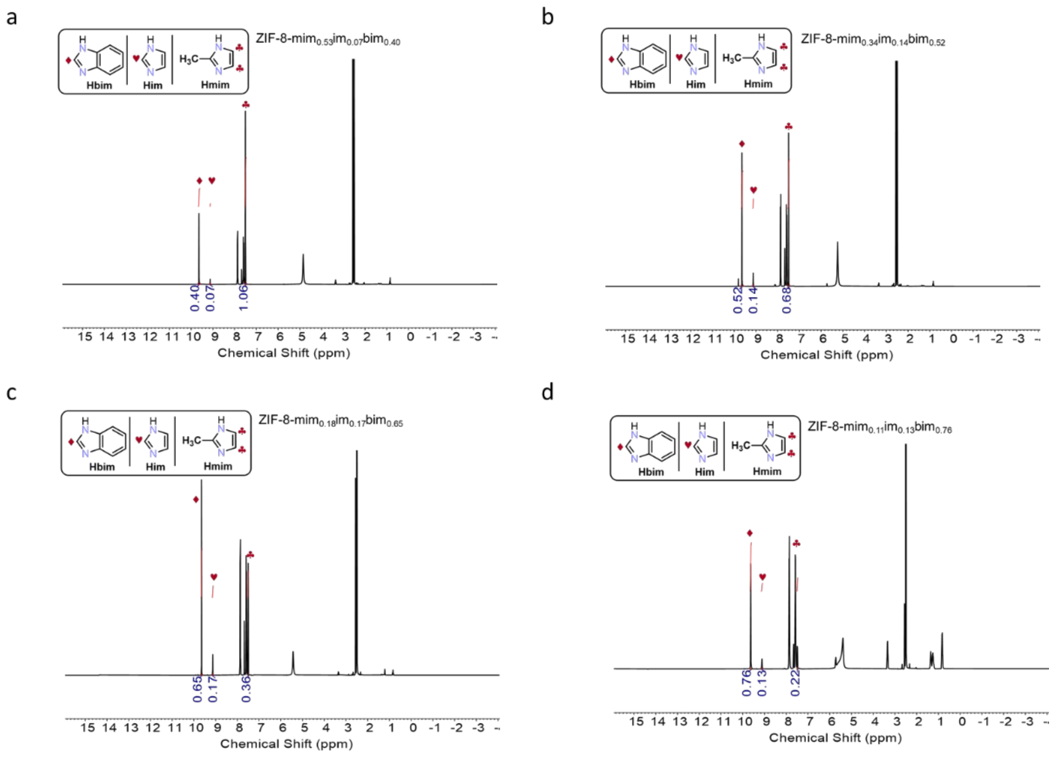

Supplementary Figure 10. ^{1}H NMR data collected for ZIF-8 derivatives after varying SALE reaction times with a solution containing Him and Hbim in a molar ratio of 4:6. The SALE time from **a** to **d** is 1, 3, 5, and 7 days, respectively.

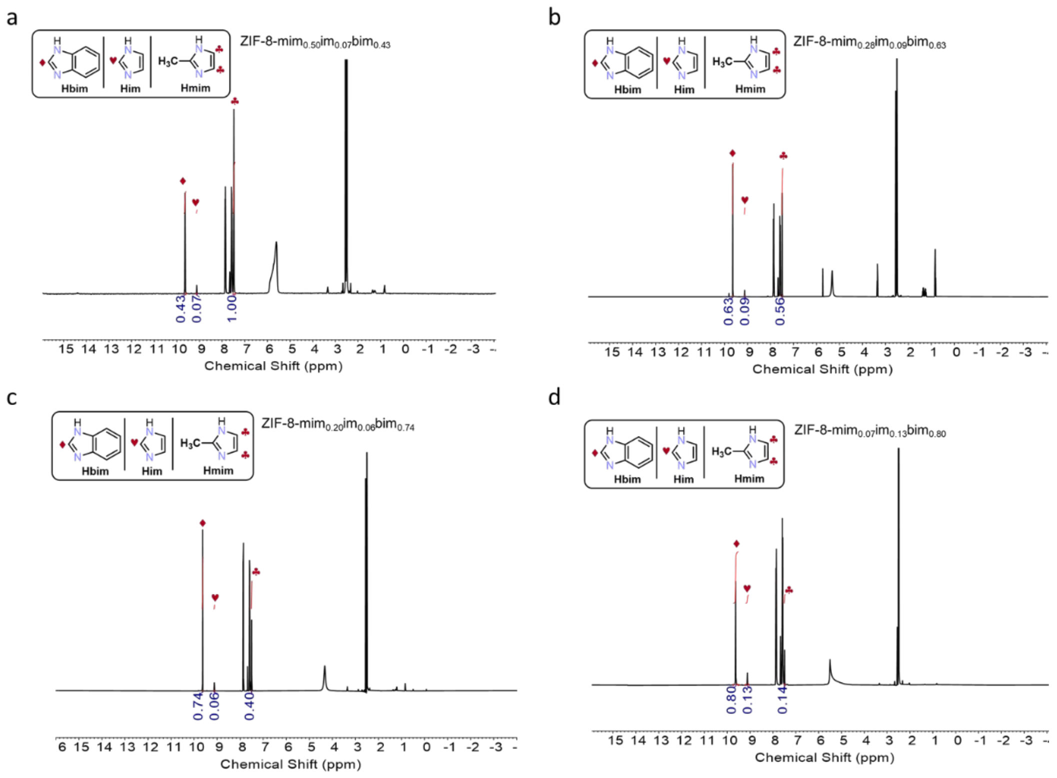

Supplementary Figure 11. ^{1}H NMR data collected for ZIF-8 derivatives after varying SALE reaction times with a solution containing Him and Hbim in a molar ratio of 3:7. The SALE time from **a** to **d** is 1, 3, 5, and 7 days, respectively.

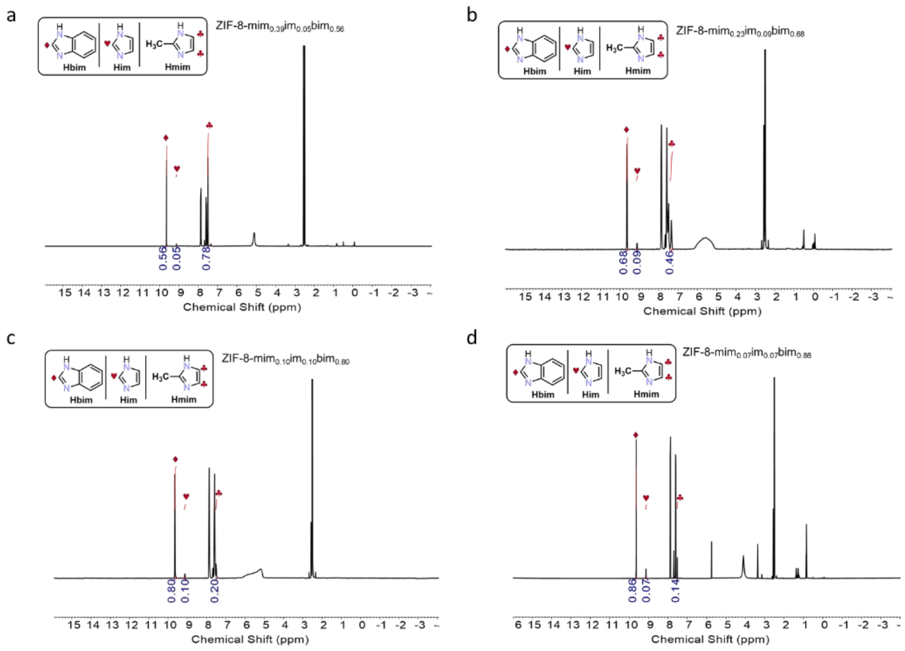

Supplementary Figure 12. ^{1}H NMR data collected for ZIF-8 derivatives after varying SALE reaction times with a solution containing Him and Hbim in a molar ratio of 2:8. The SALE time from **a** to **d** is 1, 3, 5, and 7 days, respectively.

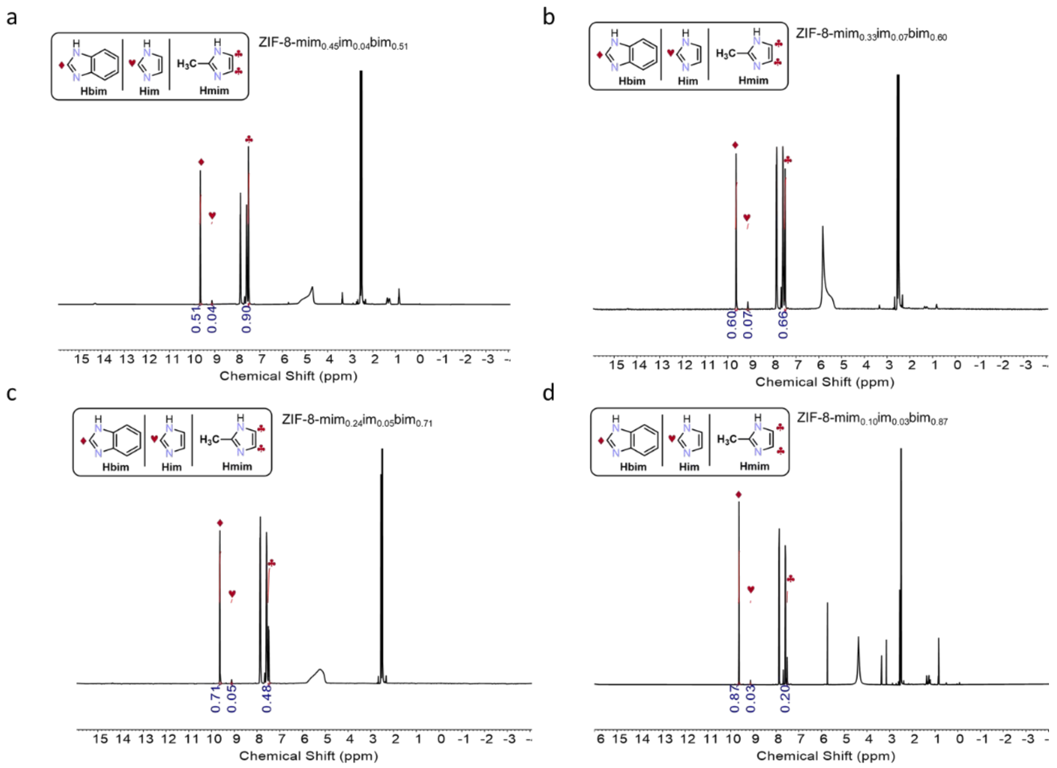

Supplementary Figure 13. ^{1}H NMR data collected for ZIF-8 derivatives after varying SALE reaction times with a solution containing Him and Hbim in a molar ratio of 1:9. The SALE time from **a** to **d** is 1, 3, 5, and 7 days, respectively.

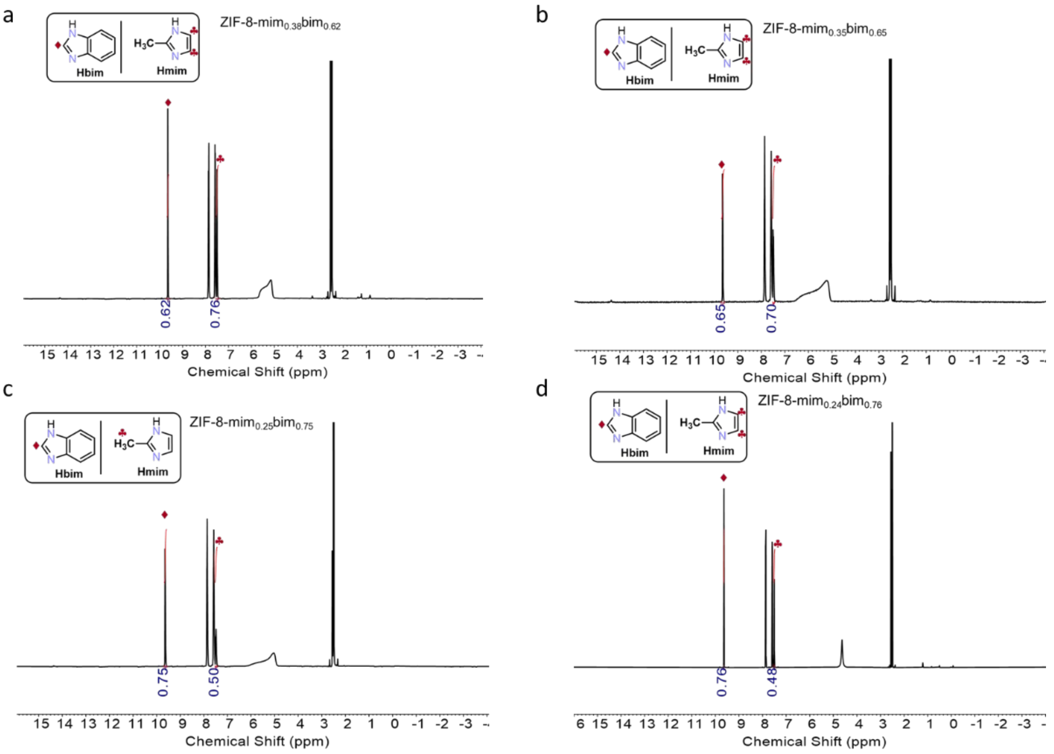

Supplementary Figure 14. ^{1}H NMR data collected for ZIF-8 derivatives after varying SALE reaction times with a solution containing only Hbim. The SALE time from **a** to **d** is 1, 3, 5, and 7 days, respectively.

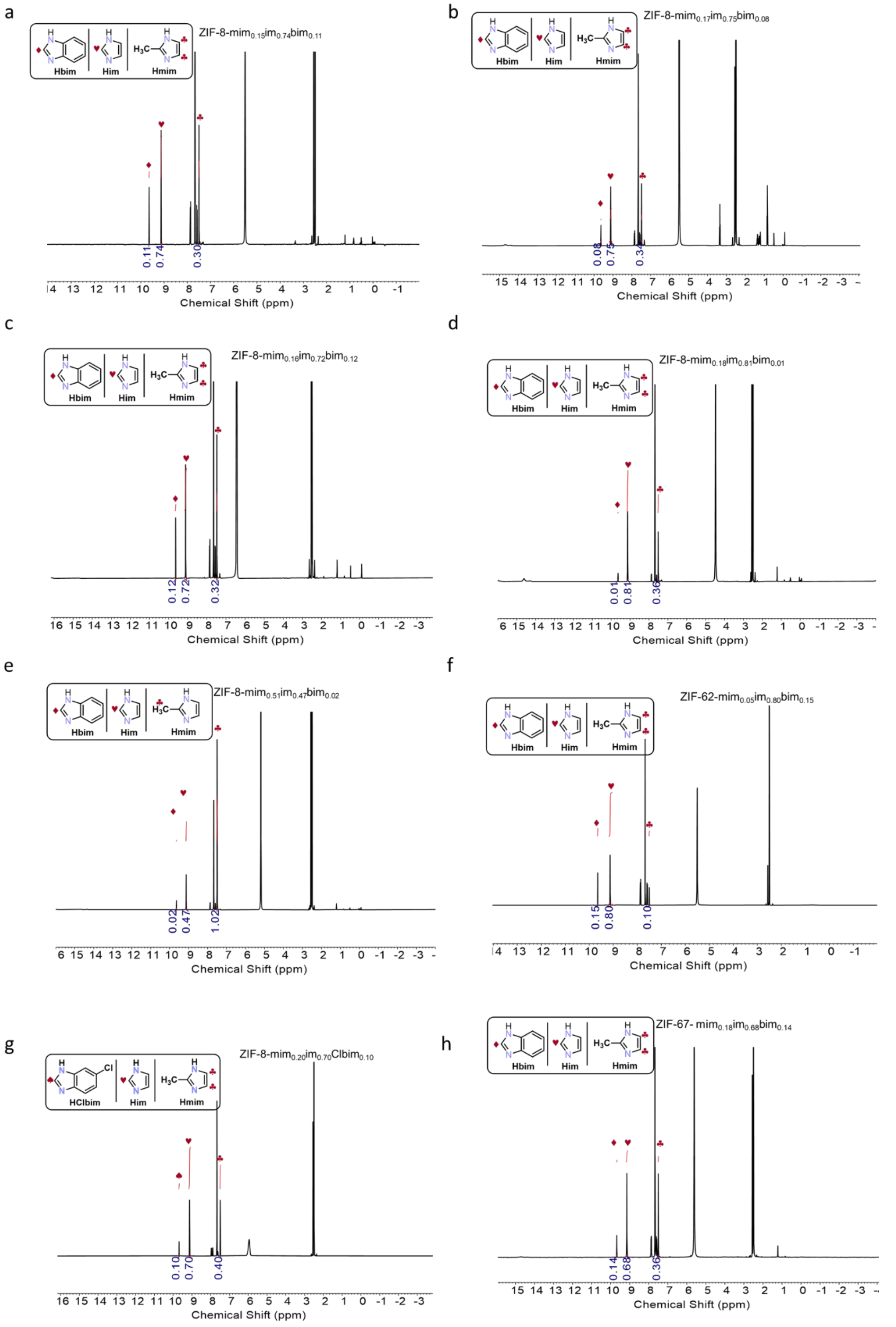

Supplementary Figure 15. ^{1}H NMR data collected for seven additional ZIF-8 derivatives and one ZIF-67 derivative.

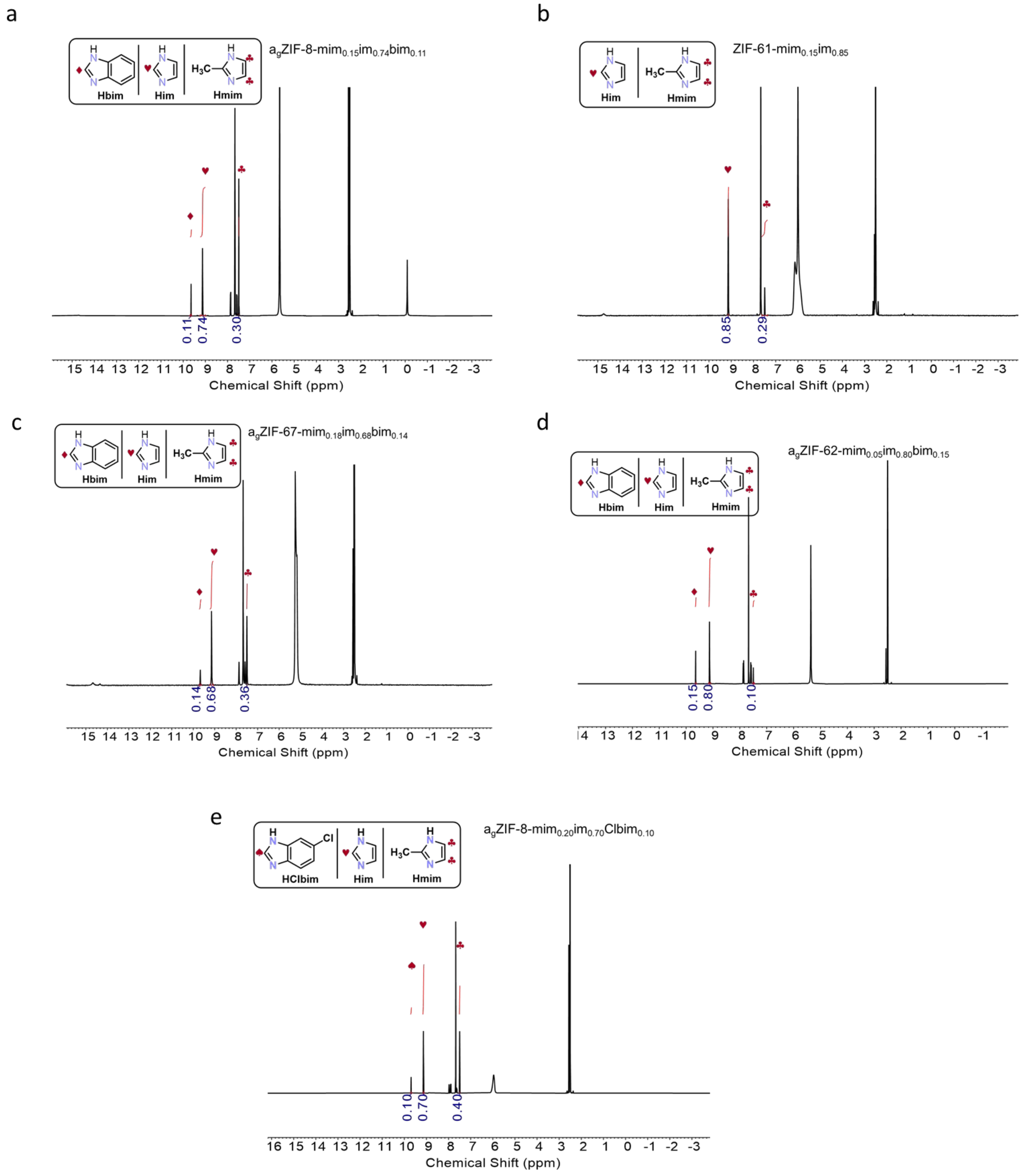

Supplementary Figure 16. ^{1}H NMR data collected for representative ZIF-8 derivatives and ZIF-67 derivative after heat treatment. **a** a_gZIF-8-$mim_{0.15}im_{0.74}bim_{0.11}$. **b** ZIF-61-$mim_{0.15}im_{0.85}$. **c** a_gZIF-67-$mim_{0.18}im_{0.68}bim_{0.14}$. **d** a_gZIF-62-$mim_{0.05}im_{0.80}bim_{0.15}$. **e** a_gZIF-8-$mim_{0.20}im_{0.70}Clbim_{0.10}$.

Supplementary Methods 2 – X-ray powder diffraction data

Supplementary Methods 2.1 – Ambient temperature XRPD

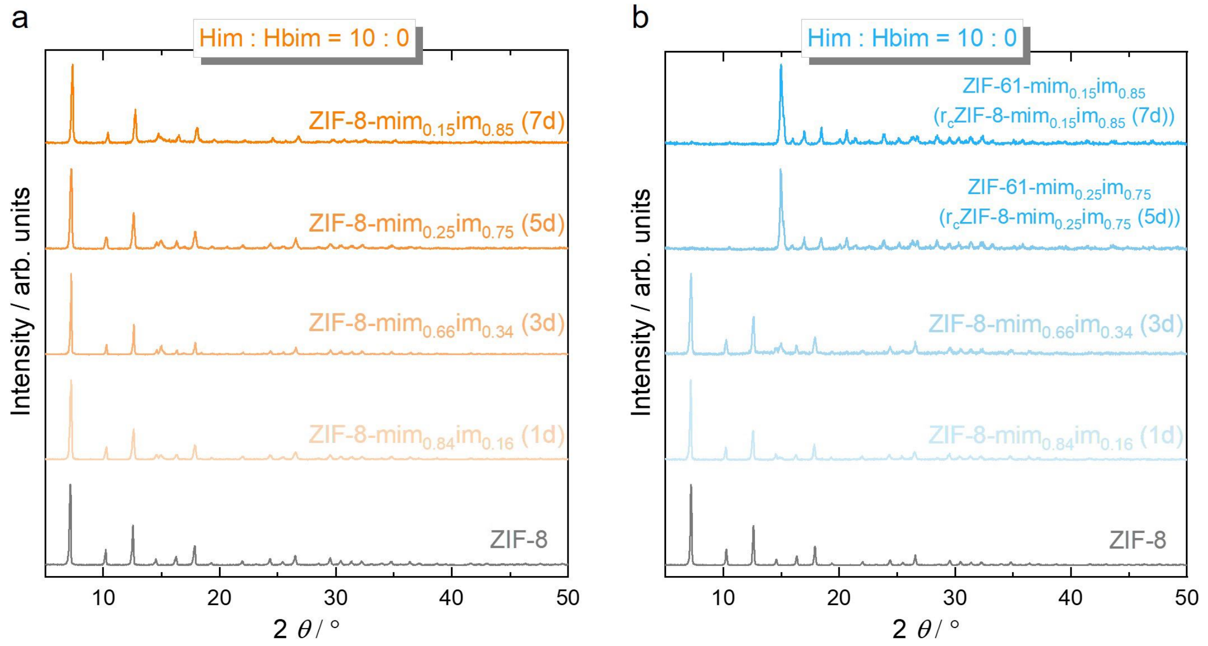

Supplementary Figure 17. a XRPD patterns collected for ZIF-8 derivatives after varying SALE reaction times with a solution containing only Him. **b** Corresponding XRPD patterns of samples obtained after heating the materials to 450 °C followed by rapid cooling to room temperature. For the SALE time of 5- and 7- day samples, the presence of a large amount of im^{-} led to the destabilization of the ZIF-8 **sod** structure and a solid-state transition to a dense **zni** topology (ZIF-61-like) was obtained by heat treatment (validated by Pawley fitting, see Supplementary Figure 33).

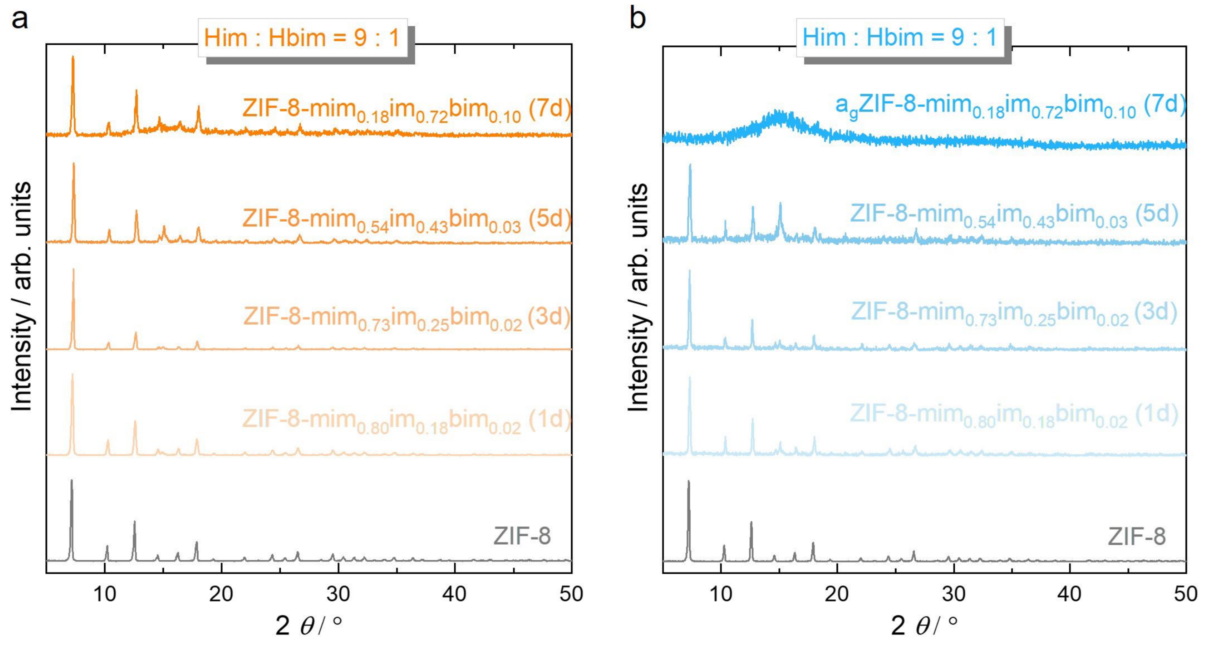

Supplementary Figure 18. a XRPD patterns collected for ZIF-8 derivatives after varying SALE reaction times with a solution containing Him and Hbim in a molar ratio of 9:1. **b** Corresponding XRPD patterns of samples obtained after heating the materials to 450 °C followed by rapid cooling to room temperature.

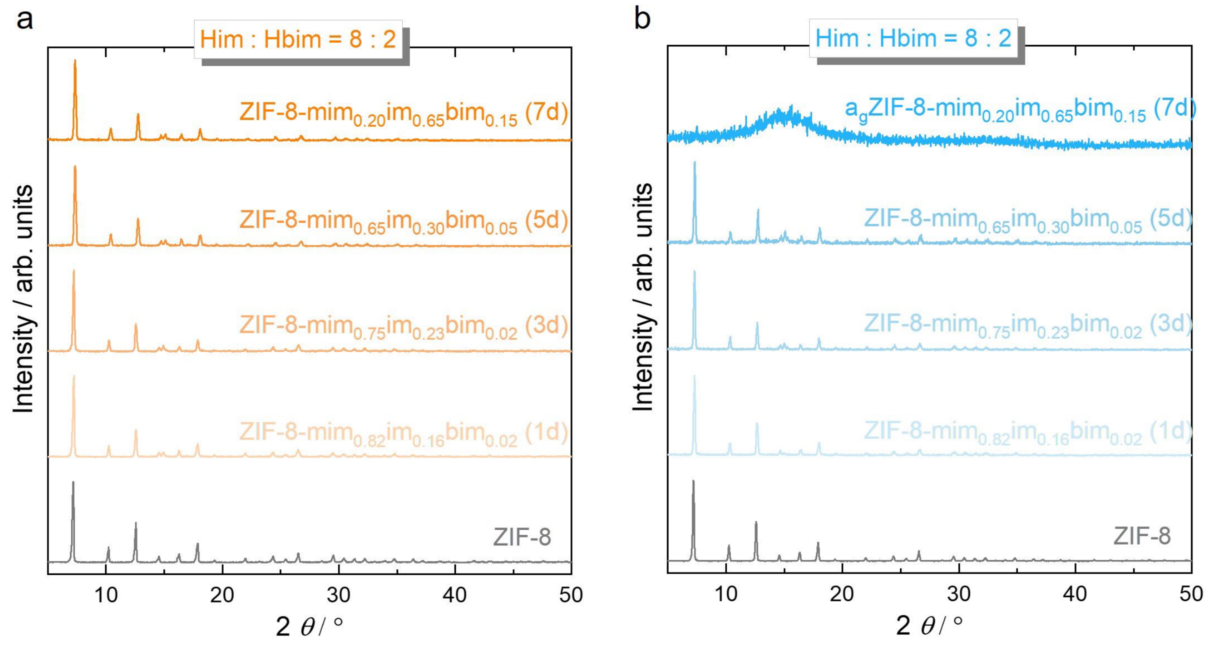

Supplementary Figure 19. a XRPD patterns collected for ZIF-8 derivatives after varying SALE reaction times with a solution containing Him and Hbim in a molar ratio of 8:2. **b** Corresponding XRPD patterns of samples obtained after heating the materials to 450 °C followed by rapid cooling to room temperature.

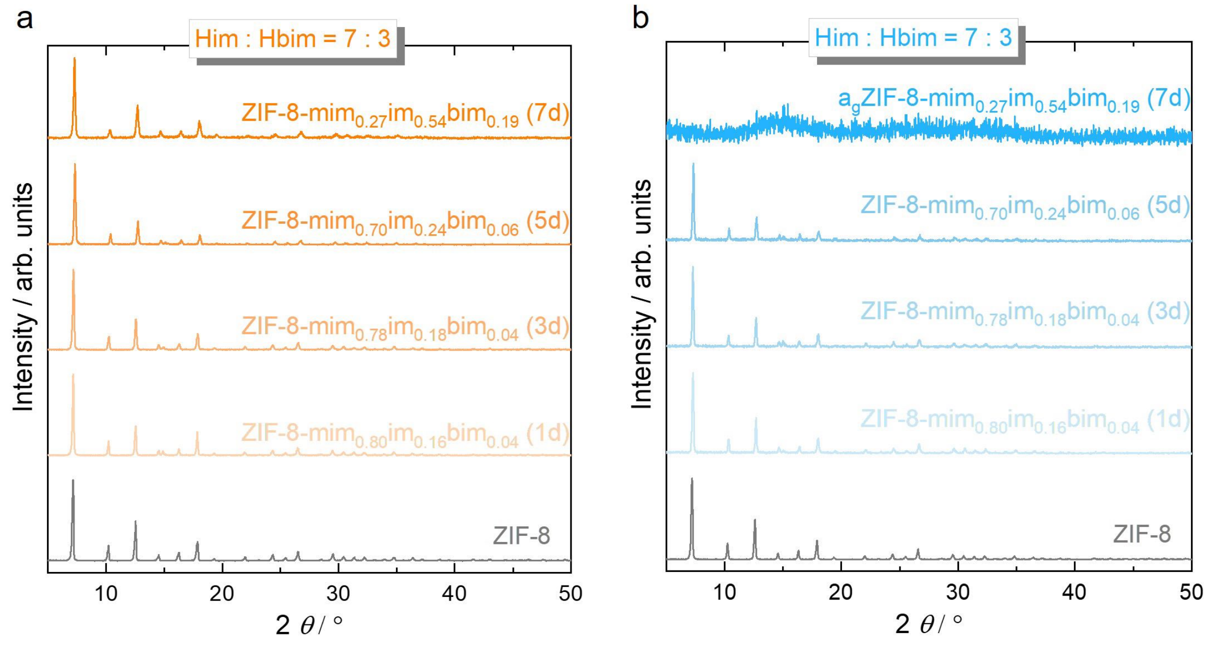

Supplementary Figure 20. A XRPD patterns collected for ZIF-8 derivatives after varying SALE reaction times with a solution containing Him and Hbim in a molar ratio of 7:3. **B** Corresponding XRPD patterns of samples obtained after heating the materials to 450 °C followed by rapid cooling to room temperature.

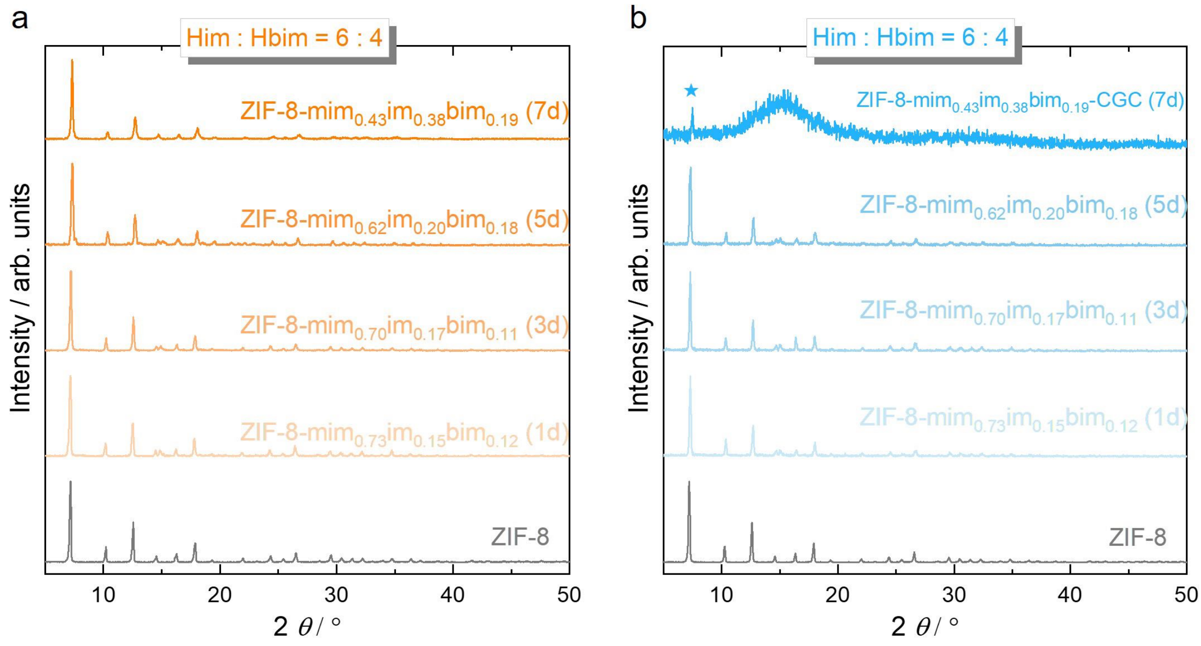

Supplementary Figure 21. A XRPD patterns collected for ZIF-8 derivatives after varying SALE reaction times with a solution containing Him and Hbim in a molar ratio of 6:4. **B** Corresponding XRPD patterns of samples obtained after heating the materials to 450 °C followed by rapid cooling to room temperature.

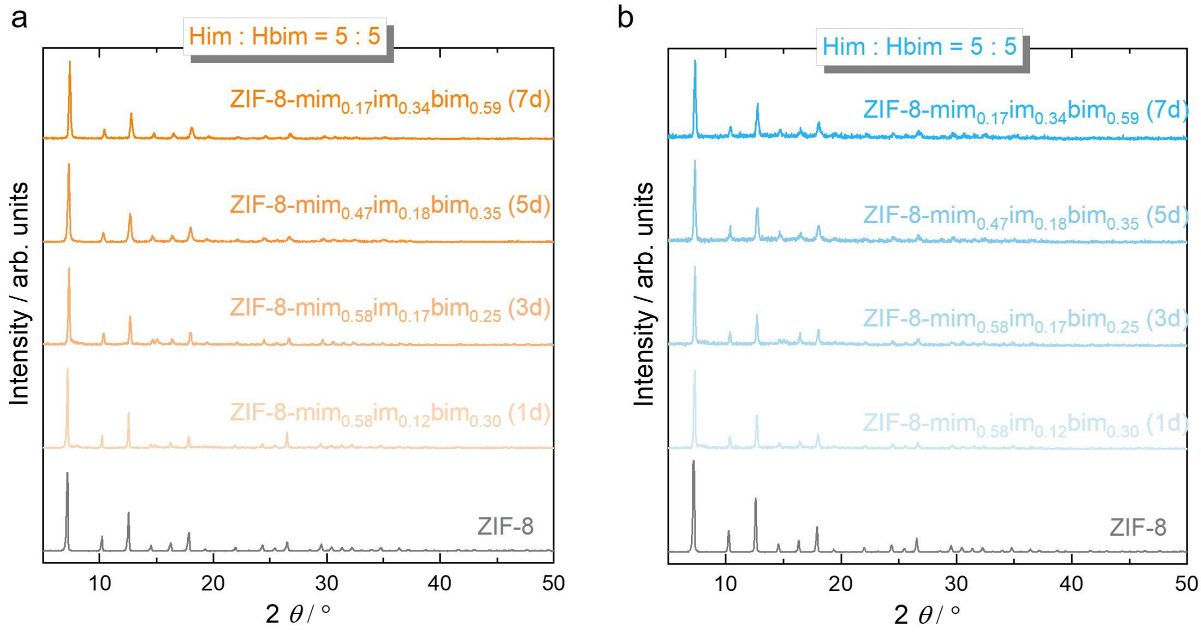

Supplementary Figure 22. a XRPD patterns collected for ZIF-8 derivatives after varying SALE reaction times with a solution containing Him and Hbim in a molar ratio of 5:5. **b** Corresponding XRPD patterns of samples obtained after heating the materials to 450 °C followed by rapid cooling to room temperature.

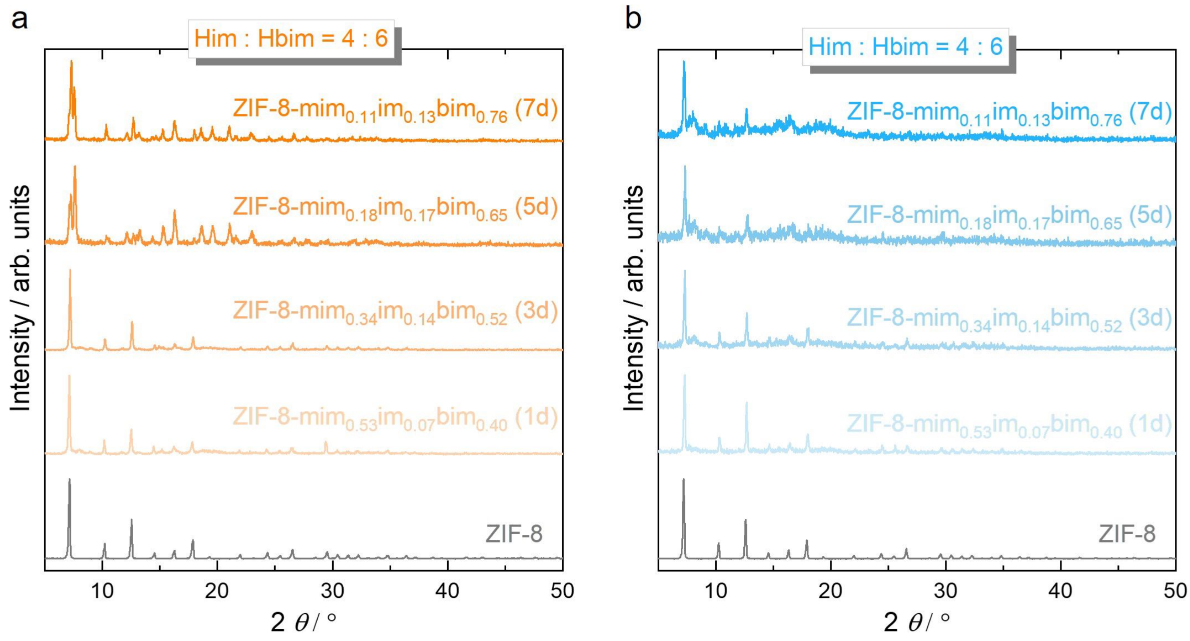

Supplementary Figure 23. a XRPD patterns collected for ZIF-8 derivatives after varying SALE reaction times with a solution containing Him and Hbim in a molar ratio of 4:6. **b** Corresponding XRPD patterns of samples obtained after heating the materials to 450 °C followed by rapid cooling to room temperature. For the samples with 5 and 7 days SALE time, the presence of a large amount of bim⁻ led to the heavily distorted rhombohedral or triclinic **sod** phase typical for ZIF-7 ($Zn(bim)_2$) (validated by Pawley fitting, see Supplementary Figure 32). The subsequent samples obtained by SALE solutions with higher molar fractions of bim⁻ were all the ZIF-7/8 mixed phase.

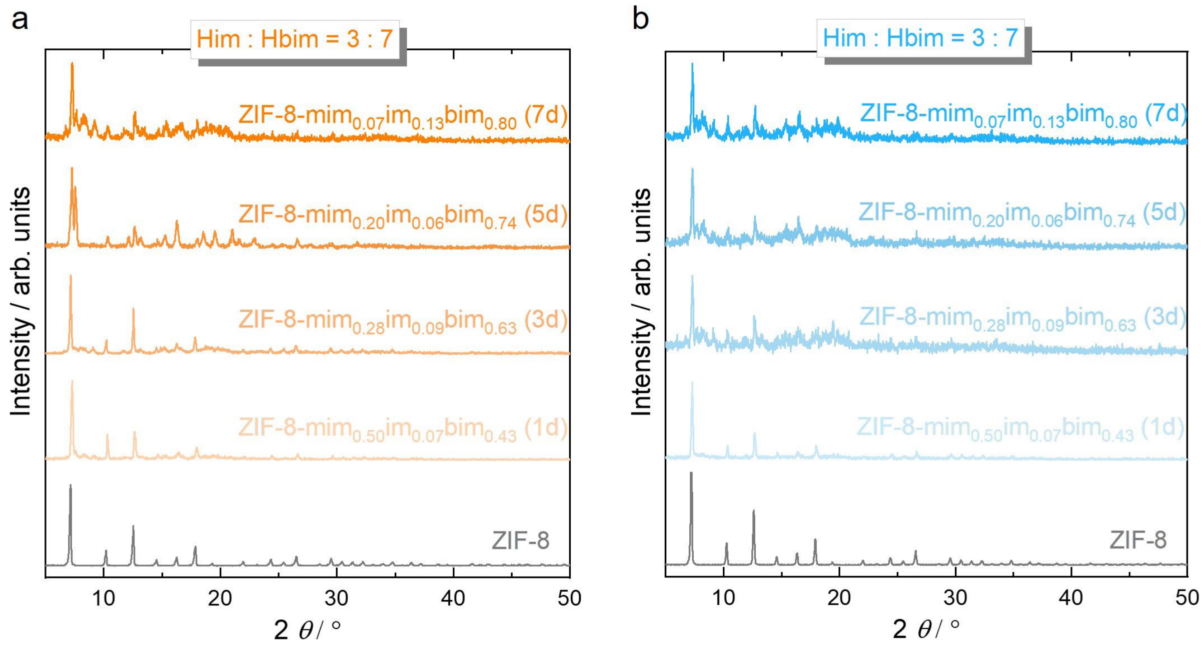

Supplementary Figure 24. a XRPD patterns collected for ZIF-8 derivatives after varying SALE reaction times with a solution containing Him and Hbim in a molar ratio of 3:7. **b** Corresponding XRPD patterns of samples obtained after heating the materials to 450 °C followed by rapid cooling to room temperature. Similar to the above, the samples after 3 and 7 days of SALE are ZIF-7/8 mixed phase.

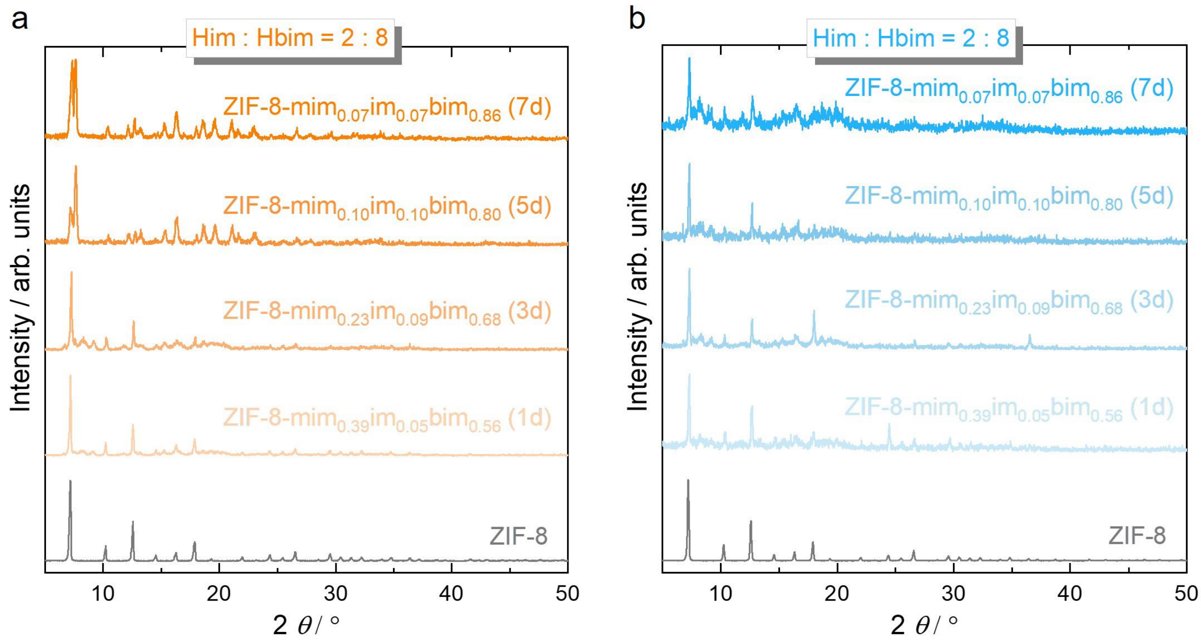

Supplementary Figure 25. a XRPD patterns collected for ZIF-8 derivatives after varying SALE reaction times with a solution containing Him and Hbim in a molar ratio of 2:8. **b** Corresponding XRPD patterns of samples obtained after heating the materials to 450 °C followed by rapid cooling to room temperature. Similar to the above, the samples after 3 and 7 days of SALE are ZIF-7/8 mixed phase.

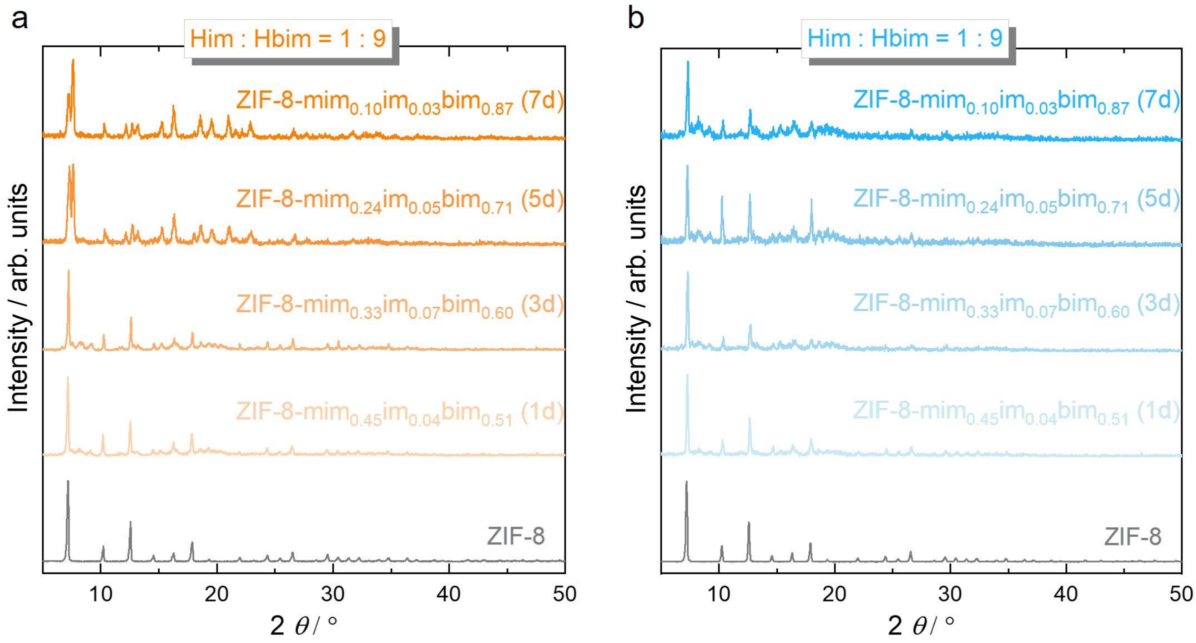

Supplementary Figure 26. a XRPD patterns collected for ZIF-8 derivatives after varying SALE reaction times with a solution containing Him and Hbim in a molar ratio of 1:9. **b** Corresponding XRPD patterns of samples obtained after heating the materials to 450 °C followed by rapid cooling to room temperature. Similar to the above, the samples after 3 and 7 days of SALE are ZIF-7/8 mixed phase.

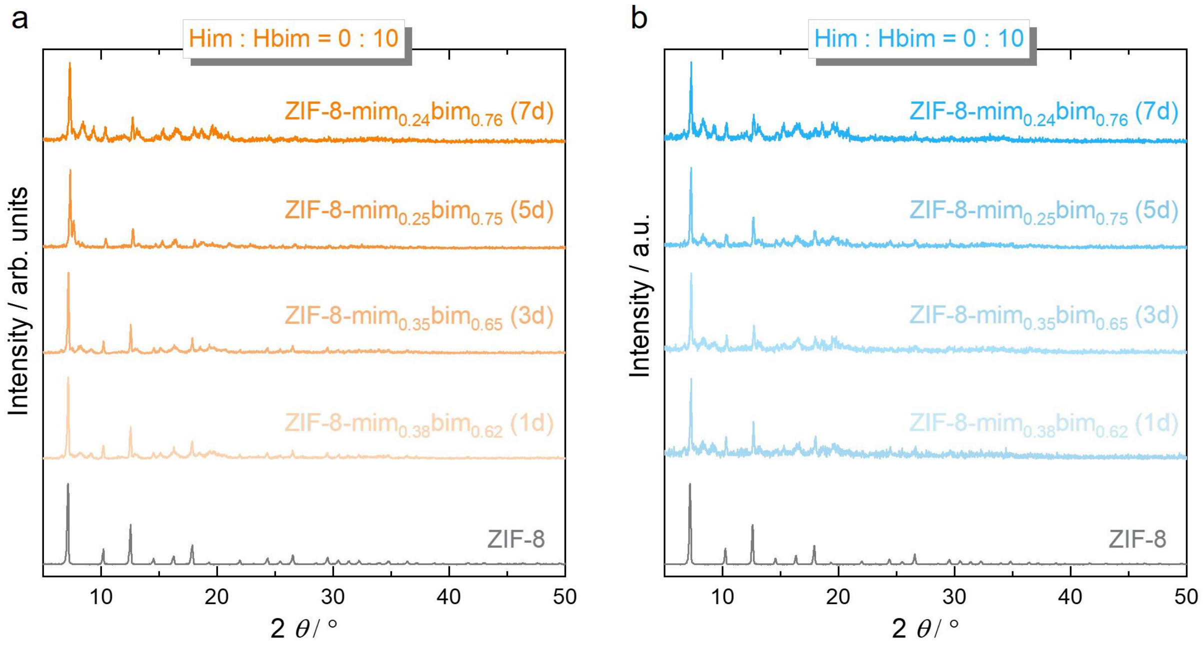

Supplementary Figure 27. a XRPD patterns collected for ZIF-8 derivatives after varying SALE reaction times with a solution containing only Hbim. **b** Corresponding XRPD patterns of samples obtained after heating the materials to 450 °C followed by rapid cooling to room temperature. Similar to the above, all the samples here are ZIF-7/8 mixed phase.

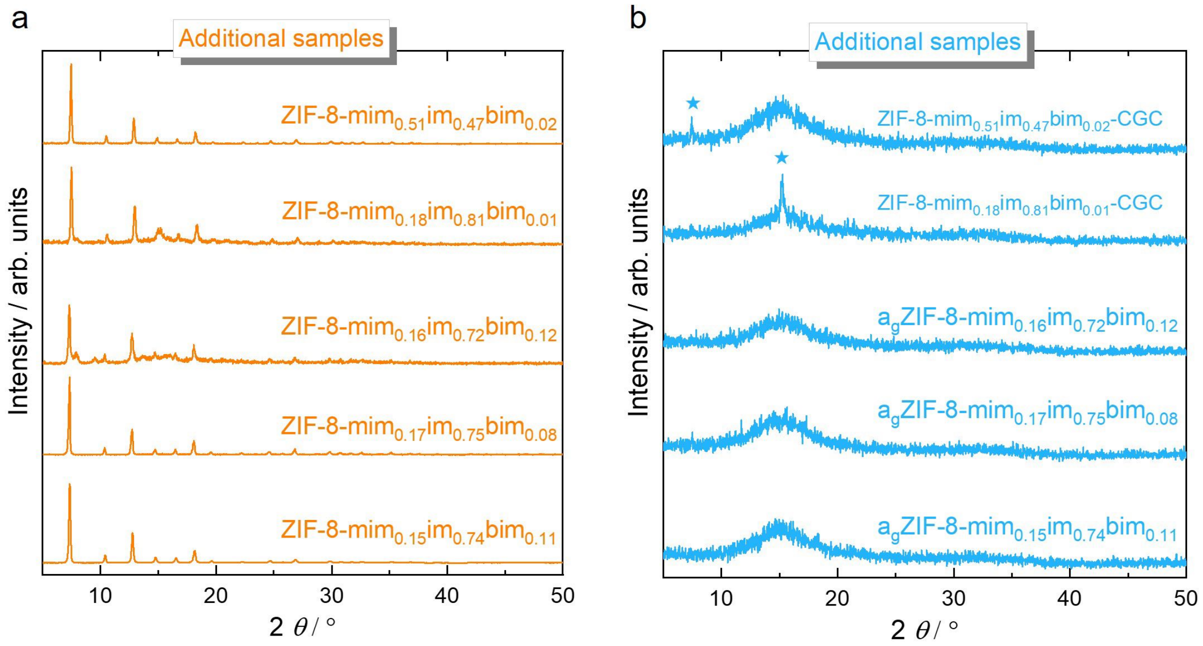

Supplementary Figure 28. a XRPD patterns collected for ZIF-8 derivatives after varying SALE reaction times with a solution containing Him and Hbim in other molar ratios (see Supplementary Table 1). **b** Corresponding XRPD patterns of samples obtained after heating the materials to 430 or 450 °C followed by rapid cooling to room temperature. The reflections marked with an asterisk stem from crystalline ZIF-8 in the case of ZIF-8-mim$_{0.51}$im$_{0.47}$bim$_{0.02}$-CGC and crystalline ZIF-61 in the case of ZIF-8-mim$_{0.18}$im$_{0.81}$bim$_{0.01}$-CGC

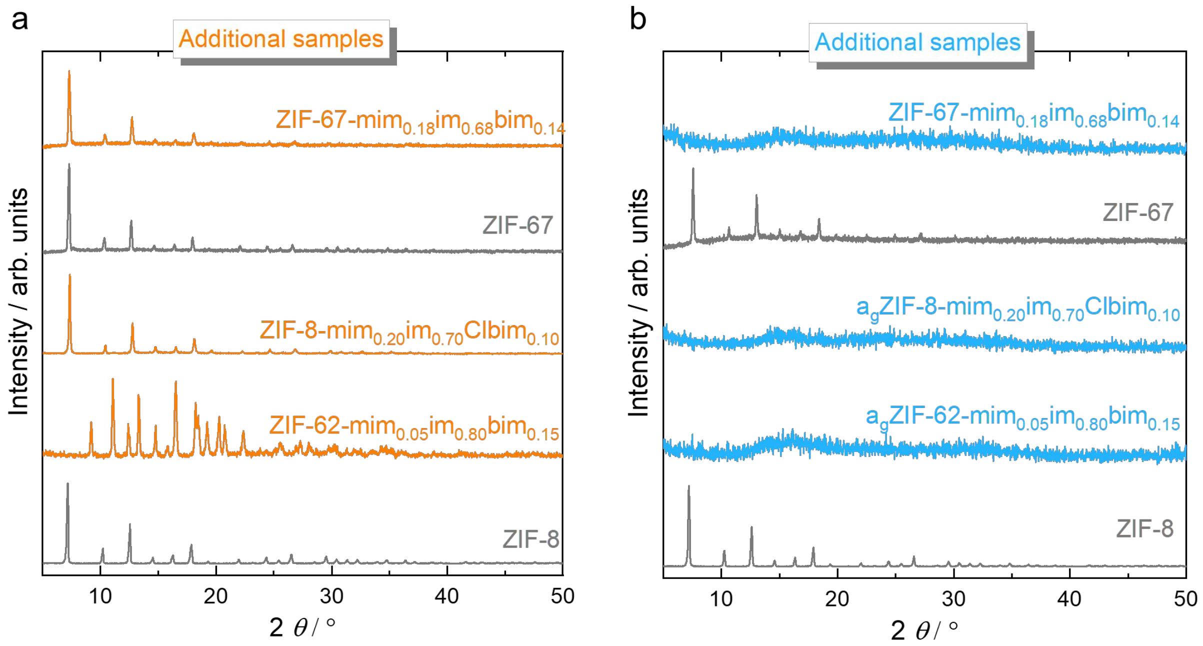

Supplementary Figure 29. a XRPD patterns collected for ZIF-8 and ZIF-67 derivatives after varying SALE reaction times with a solution containing Him and Hbim or HClbim in other molar ratios (see Supplementary Table 1). **b** Corresponding XRPD patterns of samples obtained after heating the corresponding samples to 440 or 450 °C followed by rapid cooling to room temperature. ZIF-62-$mim_{0.05}im_{0.80}bim_{0.15}$ was derived by stirring the SALE solution with ZIF-8 for 15 min per day (total SALE time was 3 days).

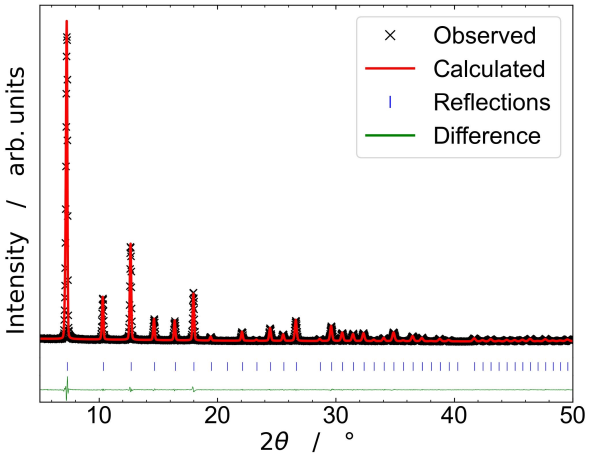

Supplementary Figure 30. Profile fit (Pawley method) performed on the XRPD pattern of ZIF-8. The blue tick marks indicate the positions of allowed Bragg peaks of crystalline ZIF-8.

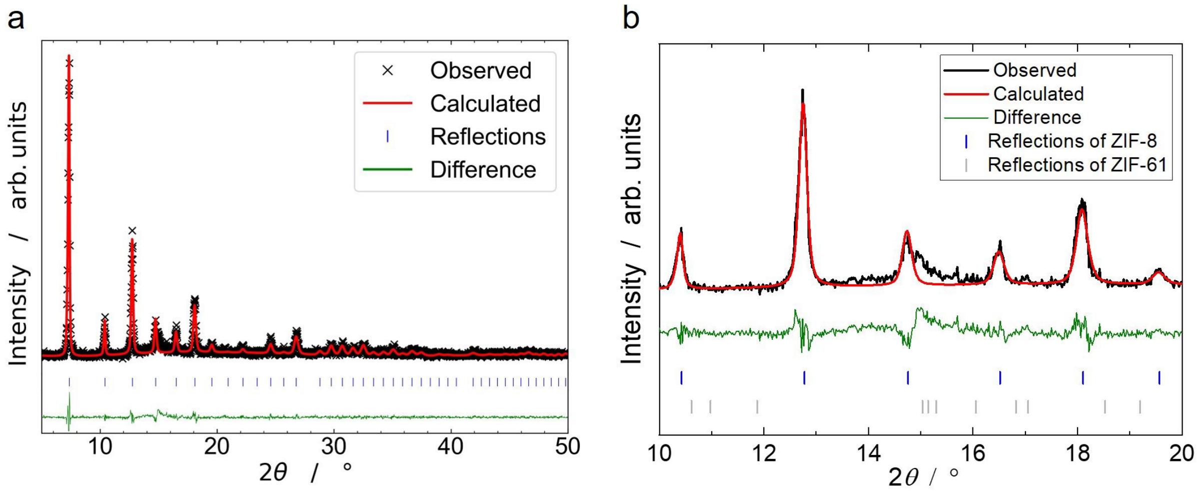

Supplementary Figure 31. a Profile fit (Pawley method) performed on the XRPD pattern of ZIF-8-mim$_{0.15}$im$_{0.85}$. The blue tick marks indicate the positions of allowed Bragg peaks for ZIF-8. **b** Magnified view of the Pawley fit showing the fraction from 10° to 20°. The part that cannot be fitted is the characteristic peak of the **zni** phase (2θ = 15.1°), i.e. ZIF-61. The blue tick marks indicate the Bragg peak positions of ZIF-8, and the gray tick marks indicate the Bragg peak positions of ZIF-61.

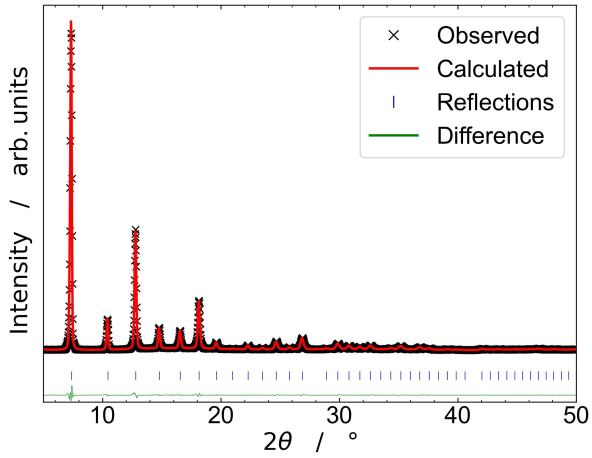

Supplementary Figure 32. Profile fit (Pawley method) performed on the XRPD pattern of ZIF-8-$mim_{0.15}im_{0.74}bim_{0.11}$. The blue tick marks indicate the positions of allowed Bragg peaks of crystalline ZIF-8.

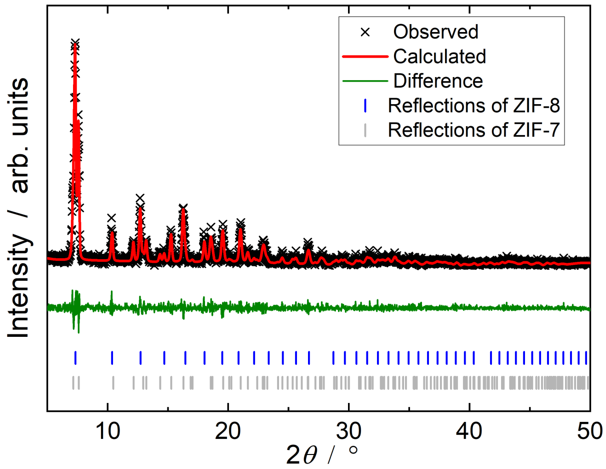

Supplementary Figure 33. Mixed-phase profile fit (Pawley method) performed on the XRPD pattern of ZIF-8-$mim_{0.11}im_{0.13}bim_{0.76}$. The blue tick marks indicate the positions of allowed Bragg peaks. The blue tick marks indicate the Bragg peak positions of ZIF-8, and the gray tick marks indicate the Bragg peak positions of ZIF-7.

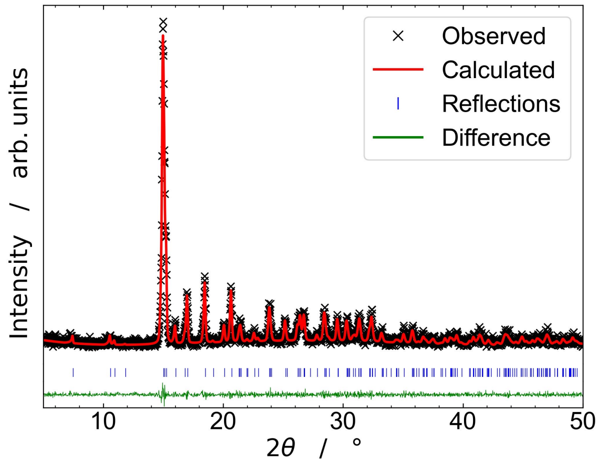

Supplementary Figure 34. Profile fit (Pawley method) performed on the XRPD pattern of ZIF-61-$mim_{0.15}im_{0.85}$ (recrystallized ZIF-8-$mim_{0.15}im_{0.85}$). The blue tick marks indicate the positions of allowed Bragg peaks of crystalline ZIF-61.

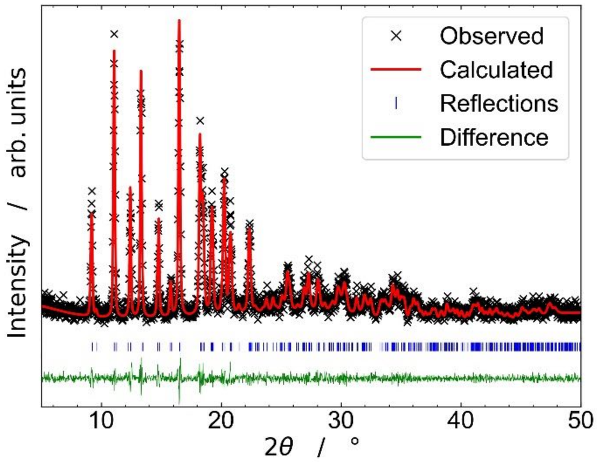

Supplementary Figure 35. Profile fit (Pawley method) performed on the XRPD pattern of ZIF-62-mim$_{0.05}$im$_{0.80}$bim$_{0.15}$ (a recrystallized ZIF-8 sample obtained by stirring the SALE reaction mixture for 15 min/day with a total reaction time of 3 days). The blue tick marks indicate the positions of allowed Bragg peaks of crystalline ZIF-62.

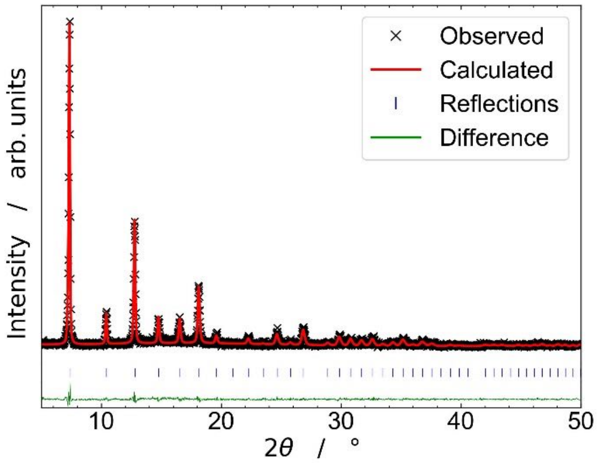

Supplementary Figure 36. Profile fit (Pawley method) performed on the XRPD pattern of ZIF-8-mim$_{0.20}$im$_{0.70}$Clbim$_{0.10}$. The blue tick marks indicate the positions of allowed Bragg peaks of crystalline ZIF-8.

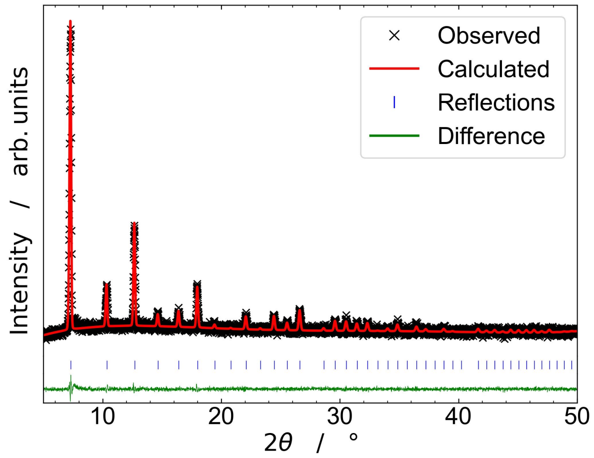

Supplementary Figure 37. Profile fit (Pawley method) performed on the XRPD pattern of ZIF-67. The blue tick marks indicate the positions of allowed Bragg peaks of crystalline ZIF-67.

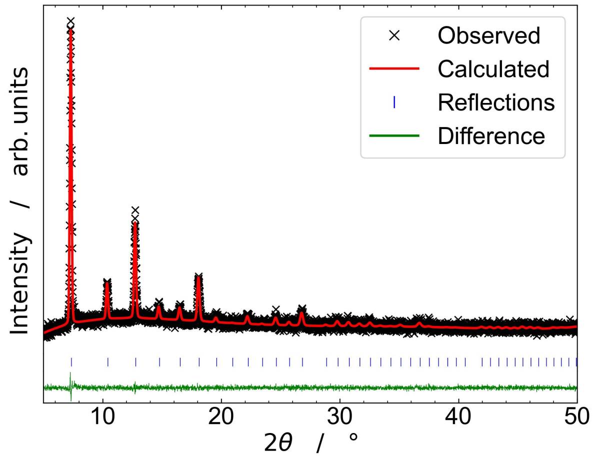

Supplementary Figure 38. Profile fit (Pawley method) performed on the XRPD pattern of ZIF-67-mim$_{0.18}$im$_{0.68}$bim$_{0.14}$. The blue tick marks indicate the positions of allowed Bragg peaks of crystalline ZIF-67.

Supplementary Table 2. Unit cell parameters and corresponding R_{wp}, R_{exp} and χ values determined by the above displayed structureless profile fits (Pawley method).

Sample	ZIF-8	ZIF-8-$mim_{0.15}im_{0.85}$	ZIF-8-$mim_{0.15}im_{0.71}bim_{0.11}$	ZIF-8-$mim_{0.11}im_{0.13}bim_{0.76}$		ZIF-61-$mim_{0.15}im_{0.85}$	ZIF-62-$mim_{0.05}im_{0.80}bim_{0.15}$	ZIF-8-$mim_{0.20}im_{0.70}$ $Clbim_{0.10}$	ZIF-67	ZIF-67-$mim_{0.18}im_{0.68}bim_{0.14}$
crystal system	cubic	cubic	cubic	Cubic (ZIF-8)	Trigonal (ZIF-7)	tetragonal	orthorhombic	cubic	cubic	cubic
space group	*I-43m*	*I-43m*	*I-43m*	*I-43m*	*R-3*	$I4_1cd$	*Pbca*	*I-43m*	*I-43m*	*I-43m*
a / Å	17.0466(10)	16.961(3)	16.9063(8)	17.009(6)	23.170(9)	23.555(8)	15.708(11)	16.913(2)	17.049(3)	16.924(6)
b / Å	17.0466(10)	16.961(3)	16.9063(8)	17.009(6)	23.170(9)	23.555(8)	15.923(10)	16.913(2)	17.049(3)	16.924(6)
c / Å	17.0466(10)	16.961(3)	16.9063(8)	17.009(6)	15.583(7)	12.490(4)	18.329(19)	16.913(2)	17.049(3)	16.924(6)
α / °	90	90	90	90	90	90	90	90	90	90
β / °	90	90	90	90	90	90	90	90	90	90
γ / °	90	90	90	90	120	90	90	90	90	90
V / Å^3	4953.5(9)	4879(3)	4832.2(7)	4921(5)	7245(6)	6930(5)	4585(6)	4838(2)	4956(3)	4848(5)
R_{wp} / %	22.37	25.04	7.75	28.03		25.61	23.30	21.00	5.56	5.47
R_{exp} / %	14.97	19.43	3.10	23.67		23.60	19.82	17.78	5.15	5.20
χ	1.49	1.29	2.50	1.18		1.09	1.18	1.18	1.08	1.05

Supplementary Methods 2.2 – Variable temperature XRPD

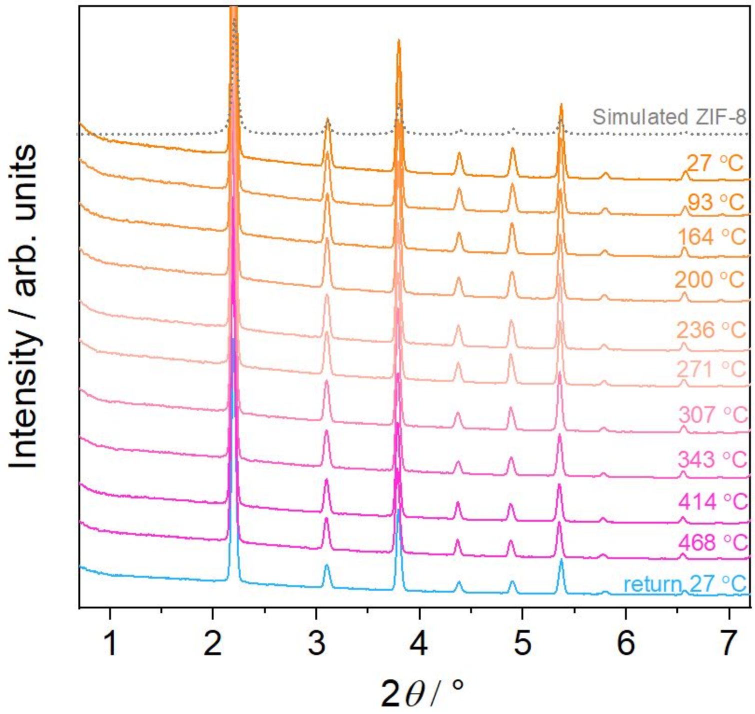

Supplementary Figure 39. Temperature dependent XRPD patterns (λ = 0.45920 Å) of ZIF-8. The patterns are not normalized but vertically offset for clarity. The corresponding contour map is shown in Figure 2a left.

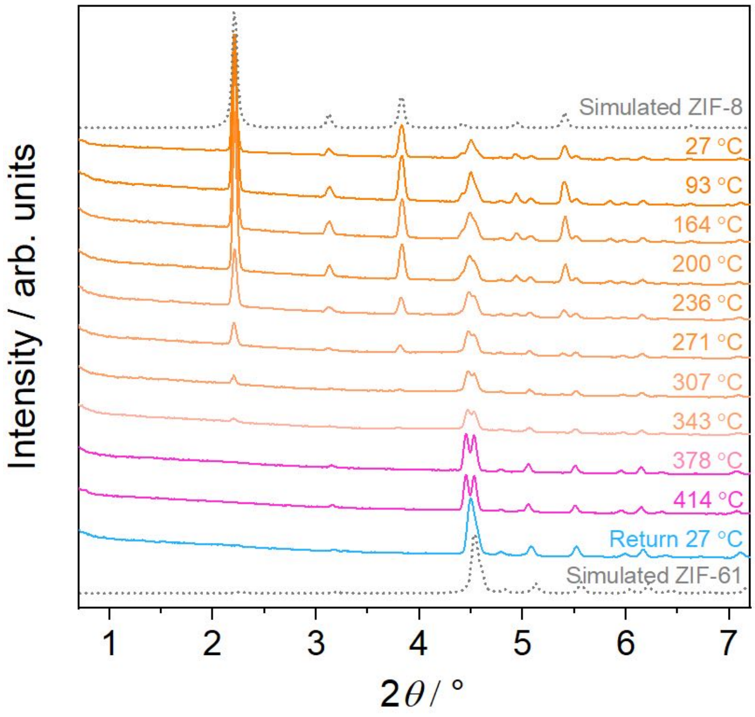

Supplementary Figure 40. Temperature dependent XRPD patterns (λ = 0.45920 Å) of ZIF-8-$mim_{0.15}im_{0.85}$. The patterns are not normalized but vertically offset for clarity. The corresponding contour map is shown in Figure 2a middle.

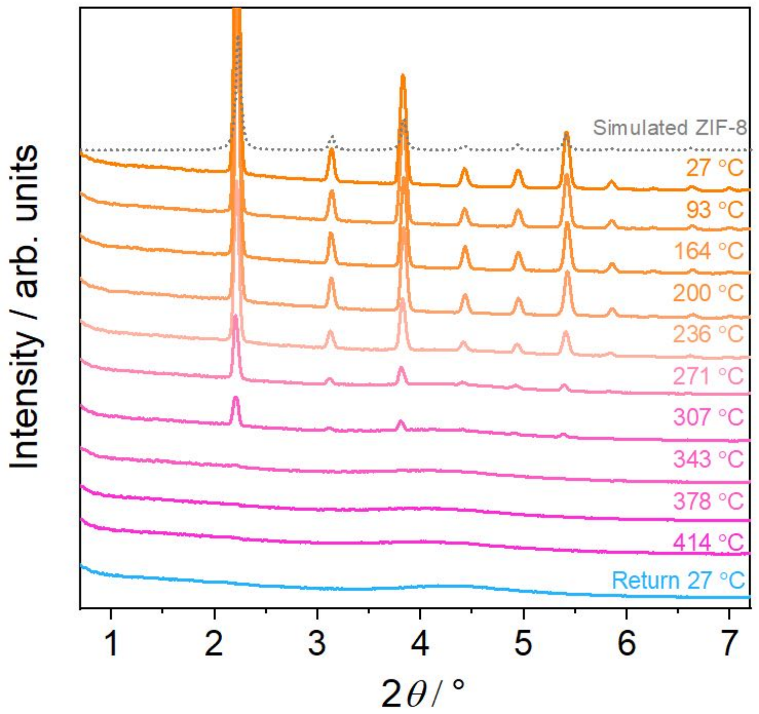

Supplementary Figure 41. Temperature dependent XRPD patterns (λ = 0.45920 Å) of ZIF-8-$mim_{0.15}im_{0.74}bim_{0.11}$. The patterns are not normalized but vertically offset for clarity. The corresponding contour map is shown in Figure 2a right.

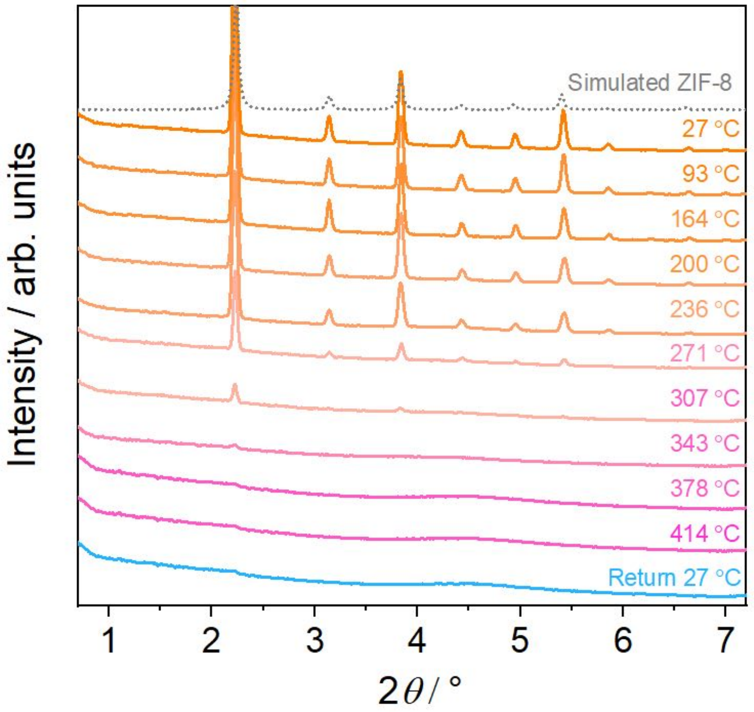

Supplementary Figure 42. Temperature dependent XRPD patterns (λ = 0.45920 Å) of ZIF-8-$mim_{0.17}im_{0.75}bim_{0.08}$. The patterns are not normalized but vertically offset for clarity.

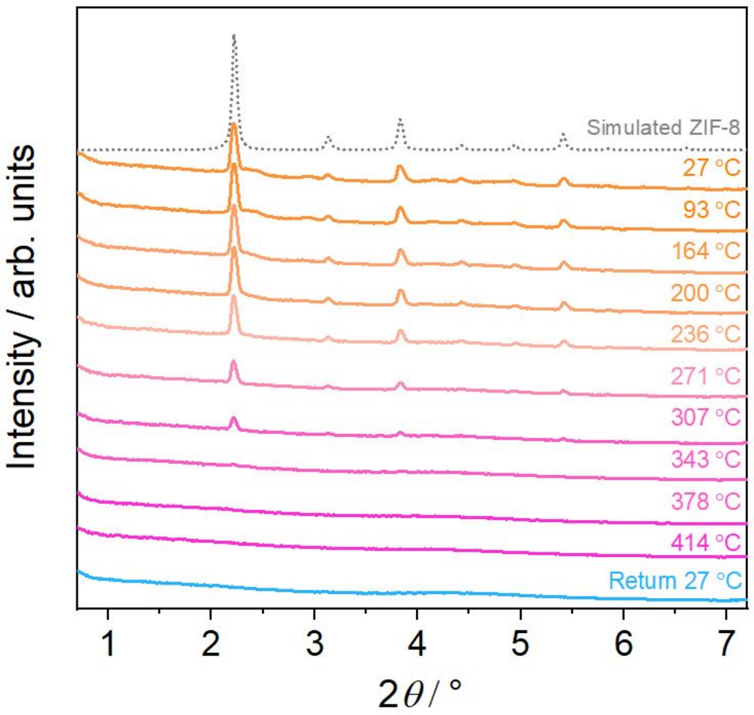

Supplementary Figure 43. Temperature dependent XRPD patterns (λ = 0.45920 Å) of ZIF-8-mim$_{0.16}$im$_{0.72}$bim$_{0.12}$. The patterns are not normalized but vertically offset for clarity.

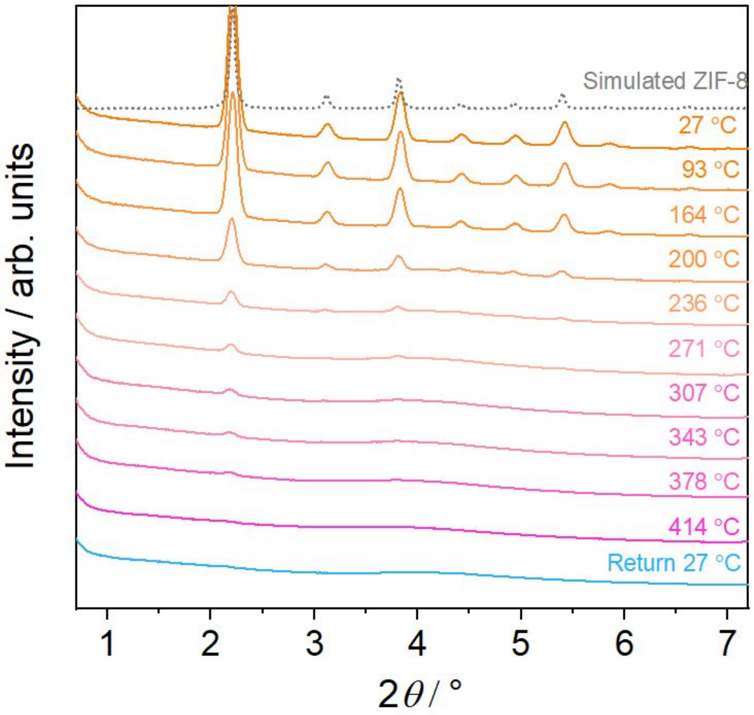

Supplementary Figure 44. Temperature dependent XRPD patterns (λ = 0.45920 Å) of ZIF-8-mim$_{0.27}$im$_{0.54}$bim$_{0.19}$. The patterns are not normalized but vertically offset for clarity.

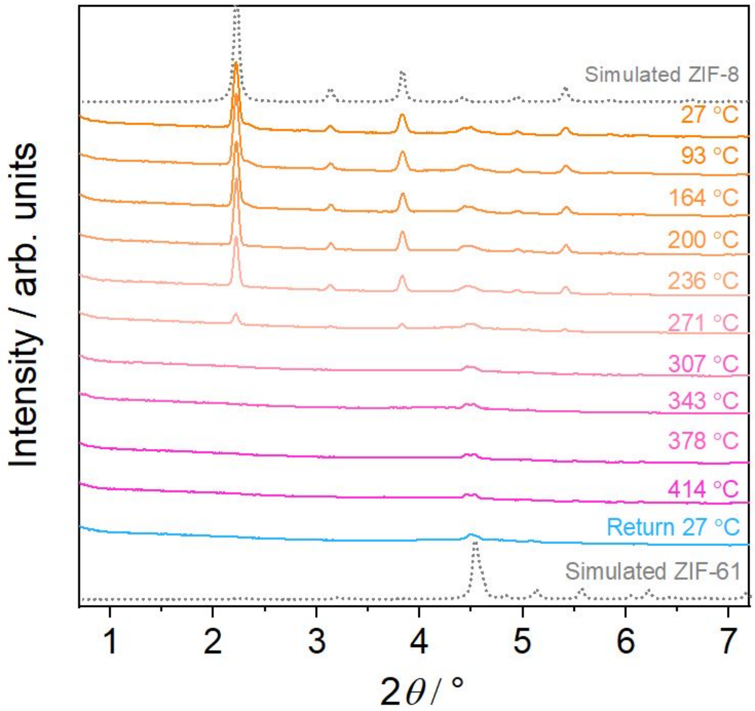

Supplementary Figure 45. Temperature dependent XRPD patterns (λ = 0.45920 Å) of ZIF-8-$mim_{0.18}im_{0.81}bim_{0.01}$. The patterns are not normalized but vertically offset for clarity.

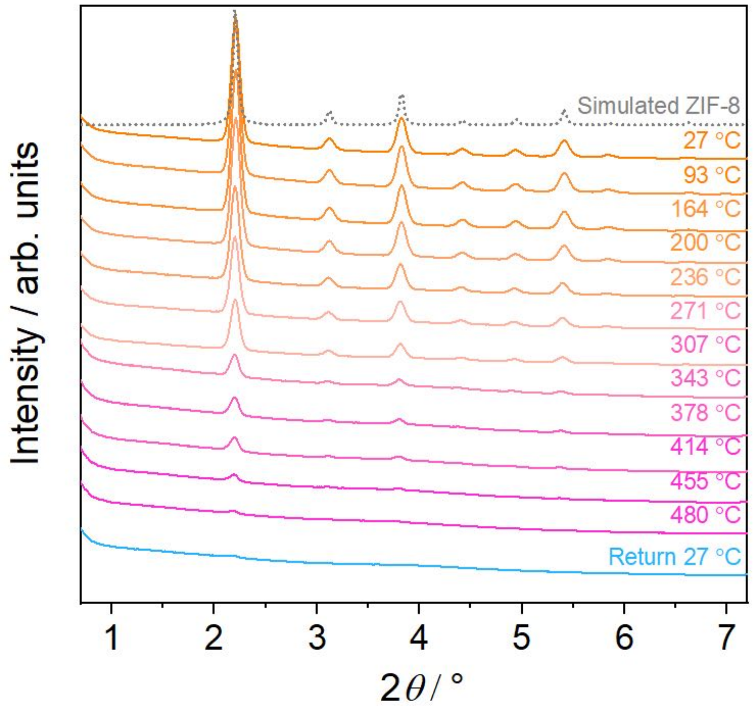

Supplementary Figure 46. Temperature dependent XRPD patterns (λ = 0.45920 Å) of ZIF-8-$mim_{0.43}im_{0.38}bim_{0.19}$. The patterns are not normalized but vertically offset for clarity.

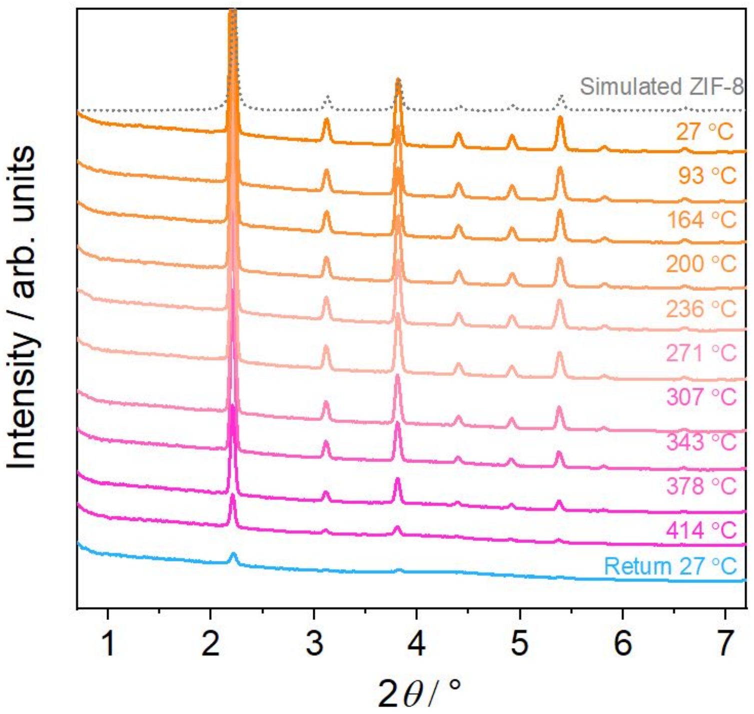

Supplementary Figure 47. Temperature dependent XRPD patterns (λ = 0.45920 Å) of ZIF-8-$mim_{0.51}im_{0.47}bim_{0.02}$. The patterns are not normalized but vertically offset for clarity.

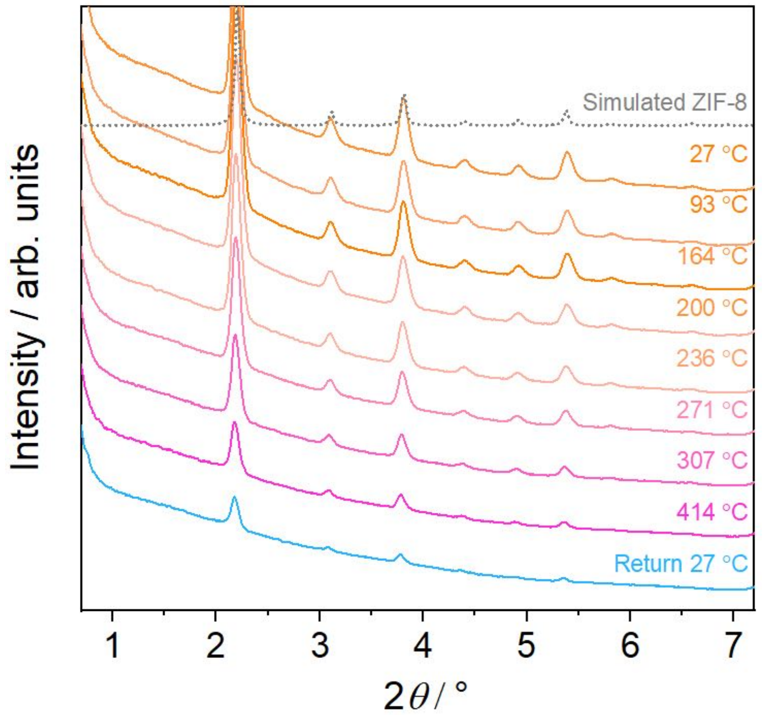

Supplementary Figure 48. Temperature dependent XRPD patterns (λ = 0.45920 Å) of ZIF-8-$mim_{0.17}im_{0.34}bim_{0.49}$. The patterns are not normalized but vertically offset for clarity.

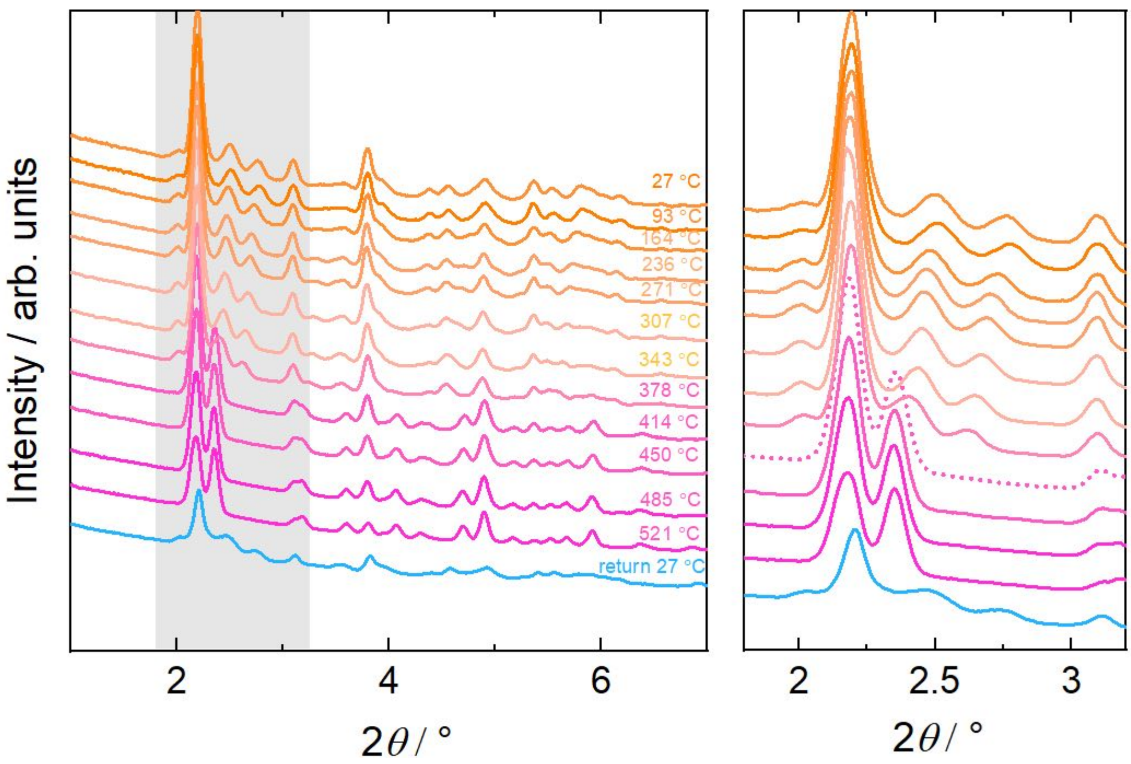

Supplementary Figure 49. Temperature dependent XRPD patterns (λ = 0.45920 Å) of ZIF-8-$mim_{0.24}bim_{0.76}$. The patterns are not normalized but vertically offset for clarity. The material clearly consists of a phase mixture of ZIF-8 (i.e. the cubic **sod** phase) and ZIF-7 (i.e. a distorted **sod** phase with triclinic symmetry at low temperature and rhombohedral symmetry at high temperature[2]).

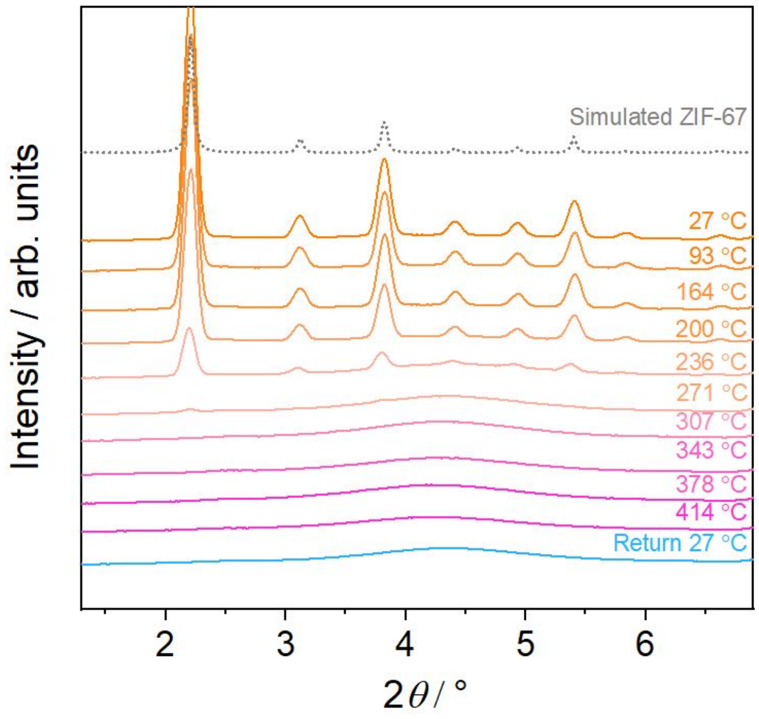

Supplementary Figure 50. Temperature dependent XRPD patterns (λ = 0.45920 Å) of ZIF-67-$mim_{0.18}im_{0.68}bim_{0.14}$. The patterns are not normalized but vertically offset for clarity.

Supplementary Methods 3 – X-ray total scattering

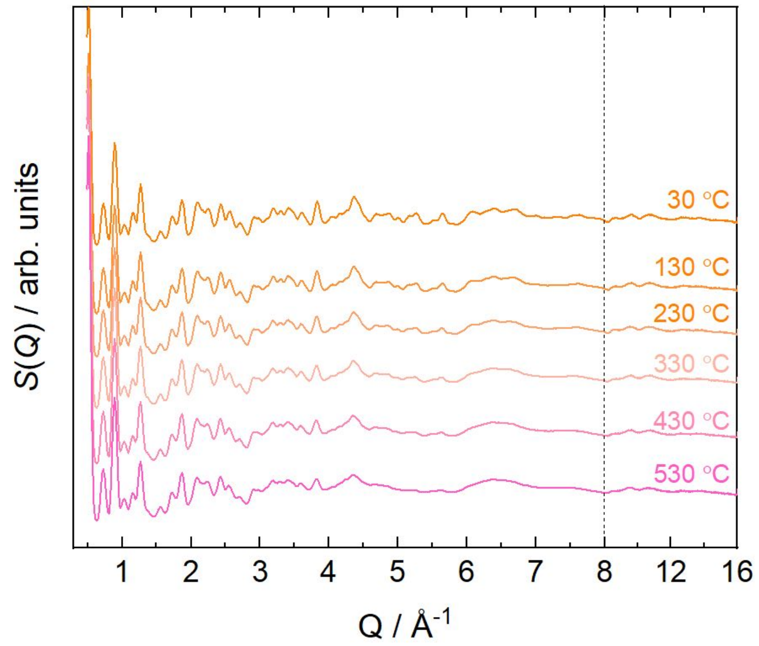

Supplementary Figure 51. Temperature dependent X-ray total scattering data in the form of *S*(*Q*) of ZIF-8. The corresponding PDF data in the form of *D*(*r*) are shown in Figure 2b. The vertical axis below 8 Å^{-1} is displayed magnified to show the variation of the scattering function in more detail.

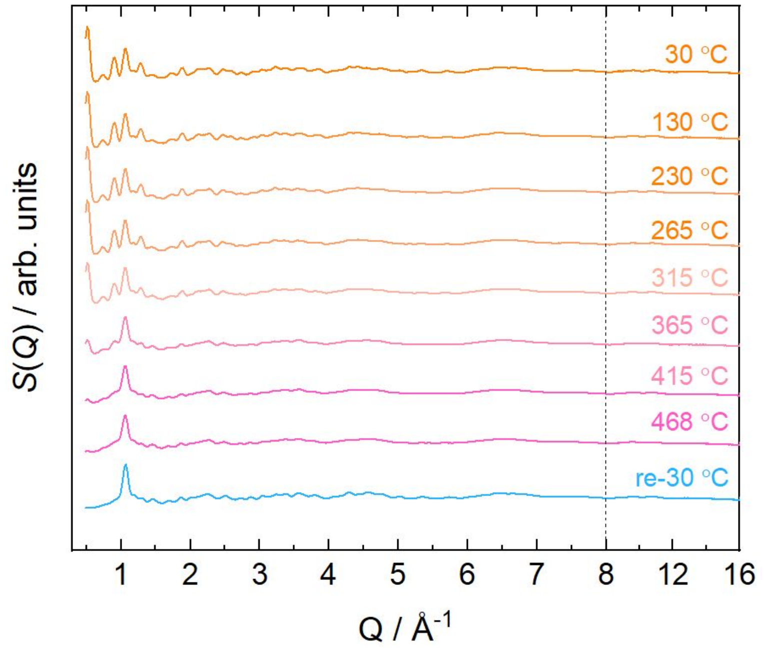

Supplementary Figure 52. Temperature dependent X-ray total scattering data in the form of *S*(*Q*) of ZIF-8-mim$_{0.15}$im$_{0.85}$. The corresponding PDF data in the form of *D*(*r*) are shown in Figure 2c. The vertical axis below 8 Å^{-1} is displayed magnified to show the variation of the scattering function in more detail.

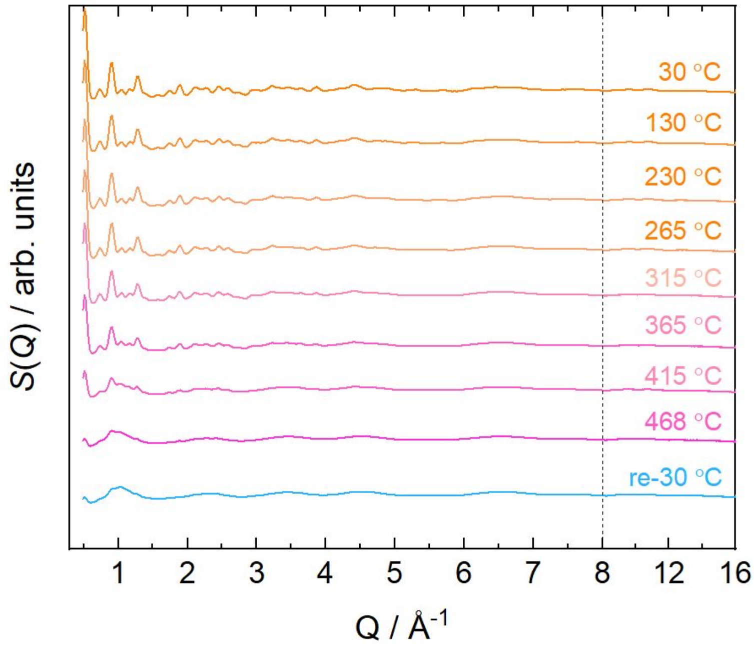

Supplementary Figure 53. Temperature dependent X-ray total scattering data in the form of *S*(*Q*) of ZIF-8-$mim_{0.15}im_{0.74}bim_{0.11}$. The corresponding PDF data in the form of *D*(*r*) are shown in Figure 2d. The vertical axis below 8 $Å^{-1}$ is displayed magnified to show the variation of the scattering function in more detail.

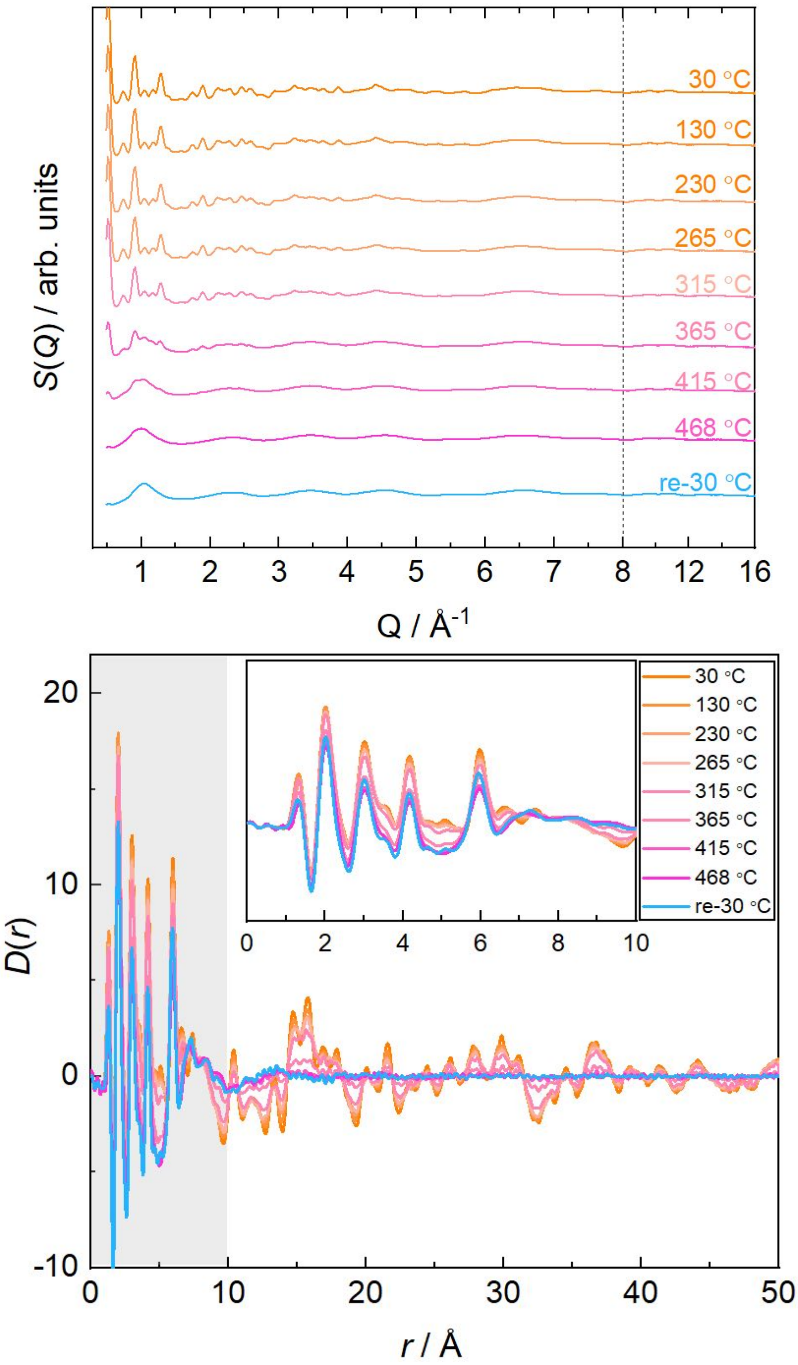

Supplementary Figure 54. Top: Temperature dependent X-ray total scattering data in the form of *S*(*Q*) data of ZIF-8-$mim_{0.17}im_{0.75}bim_{0.08}$. The vertical axis below 8 $Å^{-1}$ is displayed magnified to show the variation of the scattering function in more detail. Bottom: PDF in the form of *D*(*r*) of ZIF-8-$mim_{0.17}im_{0.75}bim_{0.08}$.

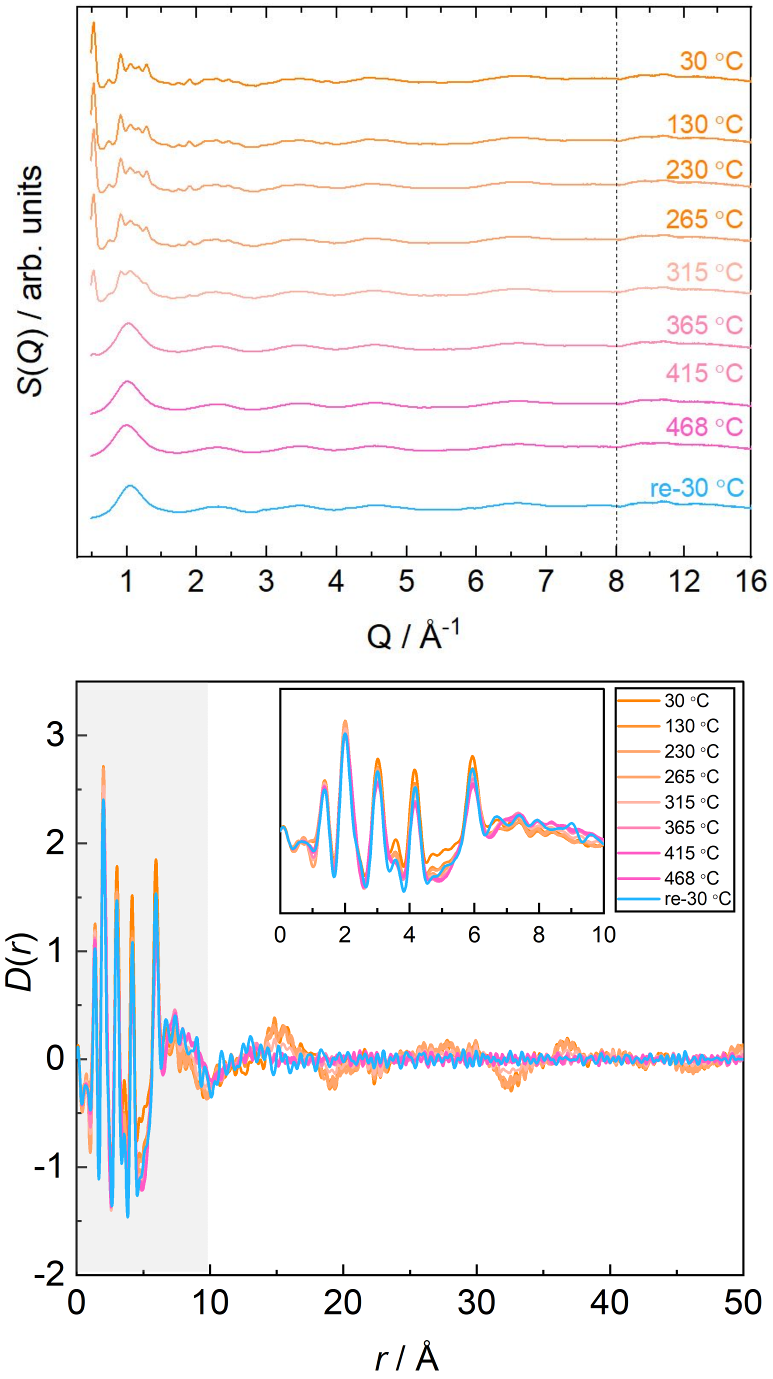

Supplementary Figure 55. Top: Temperature dependent X-ray total scattering data in the form of *S*(*Q*) data of ZIF-67-$mim_{0.18}im_{0.68}bim_{0.14}$. The vertical axis below 8 $Å^{-1}$ is displayed magnified to show the variation of the scattering function in more detail. Bottom: PDF in the form of *D*(*r*) of ZIF-67-$mim_{0.18}im_{0.68}bim_{0.14}$.

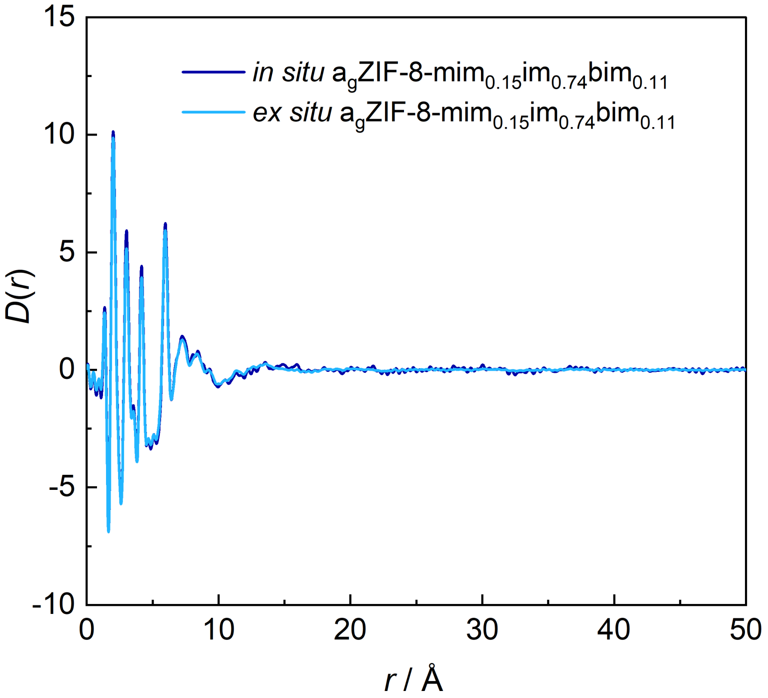

Supplementary Figure 56. Room temperature PDFs in the form *D*(*r*) of a_gZIF-8-$mim_{0.15}im_{0.74}bim_{0.11}$. The dark blue function is derived from the VT total scattering experiment by *in situ* heating to 468 °C and returning to room temperature, the light blue sample (*ex situ*) is prepared with the DSC instrument by heating to 430 °C followed by cooling to room temperature under N_2.

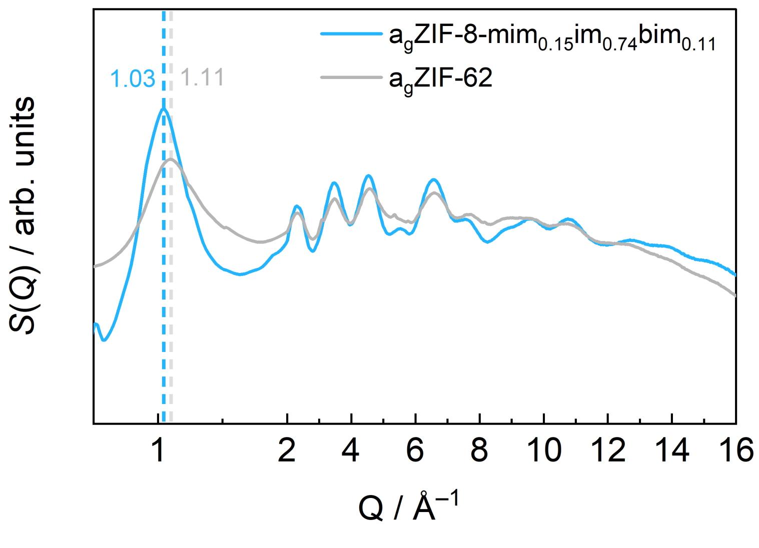

Supplementary Figure 57. Comparison of X-ray total scattering data in the form of *S*(*Q*) of a_gZIF-8-$mim_{0.15}im_{0.74}bim_{0.11}$ with a_gZIF-62. The smaller magnitude of the scattering vector for the first sharp scattering peak of a_gZIF-8-$mim_{0.15}im_{0.74}bim_{0.11}$ (1.03 $Å^{-1}$) compared to a_gZIF-62 (1.11 $Å^{-1}$) is an indication for the lower density and higher porosity of the former.

Supplementary Methods 4 – Solid state 2D spin diffusion NMR spectroscopy

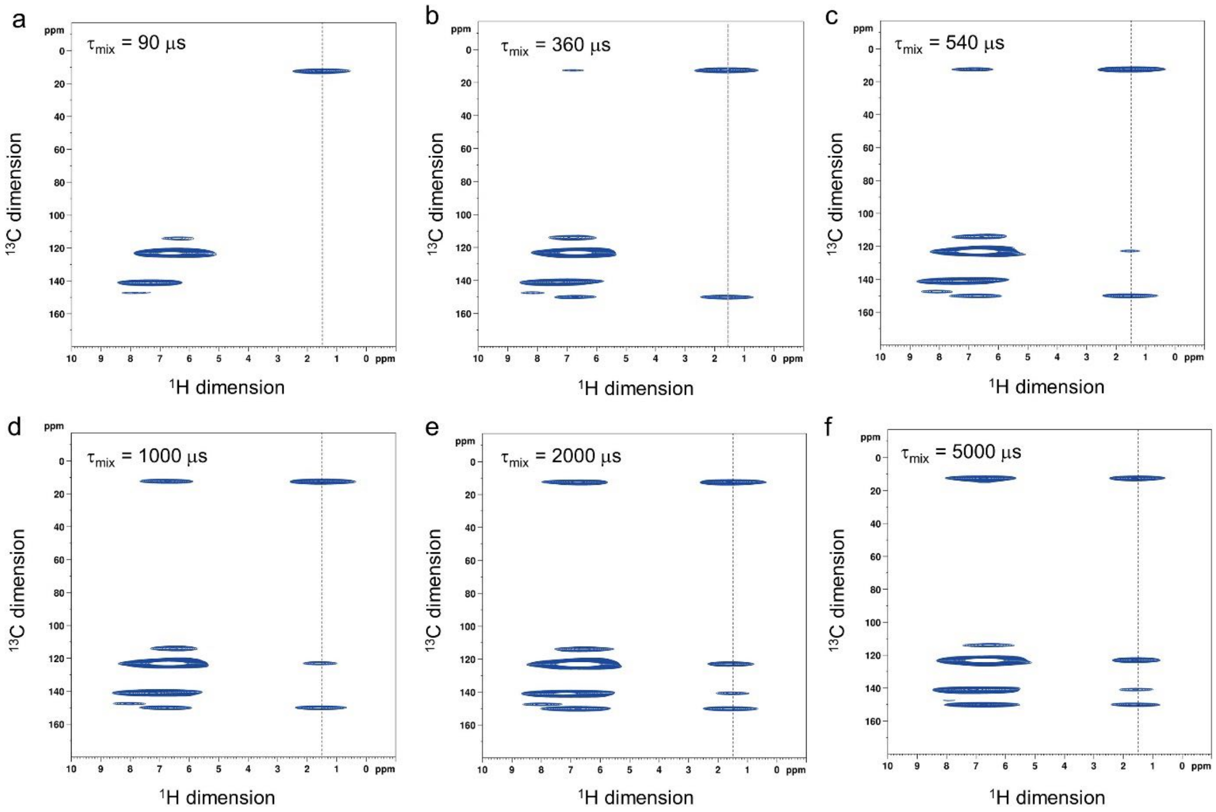

Supplementary Figure 58. ^{1}H–^{13}C proton-detected 2D proton-spin-diffusion spectra of the microcrystalline ZIF-8-mim$_{0.15}$im$_{0.74}$bim$_{0.11}$ sample recorded with different CP times. Vertical dashed lines indicate the magnetization transfer from –CH_3 group to the imidazolate rings.

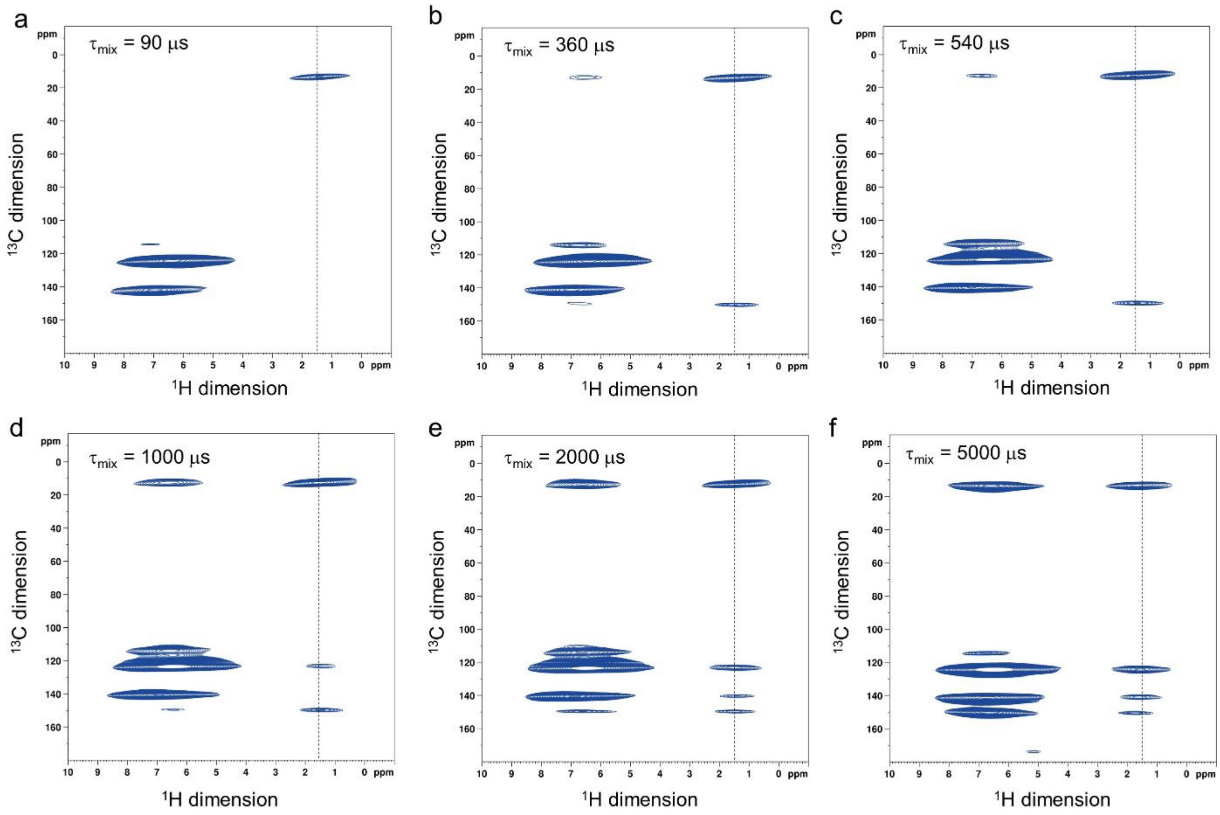

Supplementary Figure 59. ^{1}H–^{13}C proton-detected 2D proton-spin-diffusion spectra of the glassy a_gZIF-8-mim$_{0.15}$im$_{0.74}$bim$_{0.11}$ sample recorded with different CP times. Vertical dashed lines indicate the magnetization transfer from $–CH_3$ group to the imidazolate rings.

Supplementary Methods 5 – FTIR spectra

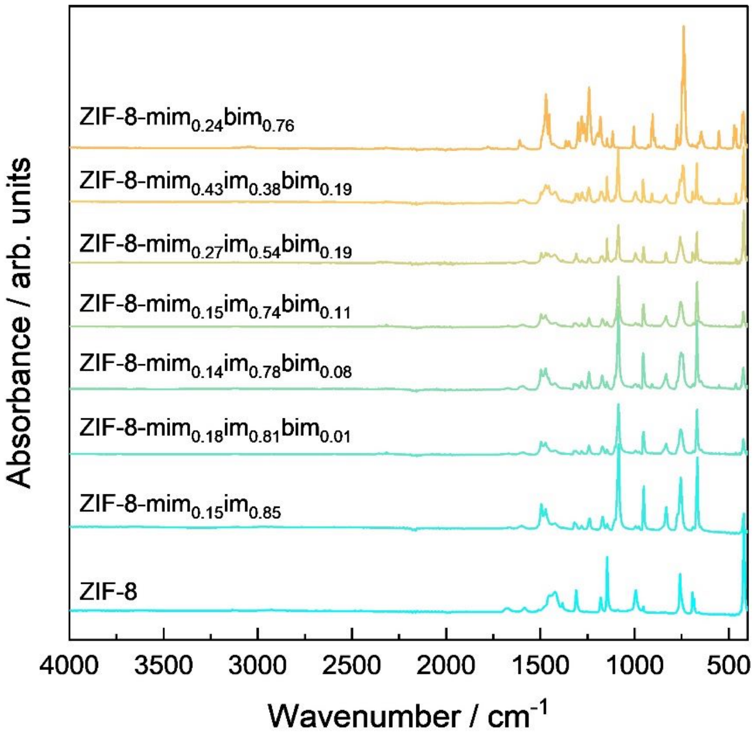

Supplementary Figure 60. FTIR spectra of selected ZIF-8 derivatives after SALE and activation. The selected samples correspond to the ones in Figure 1b.

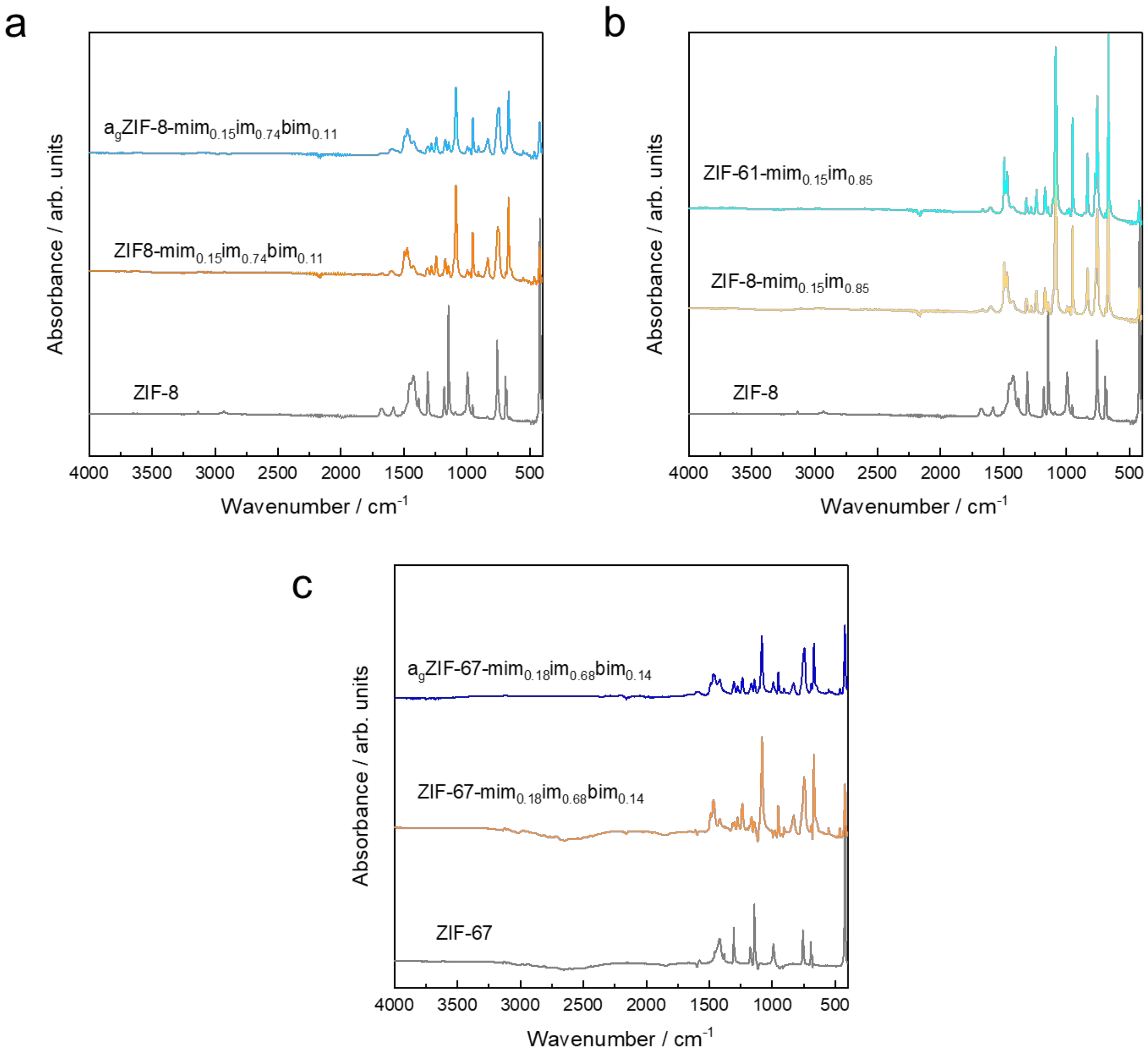

Supplementary Figure 61. Comparison of FTIR spectra of selected ZIF samples. **a** ZIF-8, ZIF-8-$mim_{0.15}im_{0.74}bim_{0.11}$, and a_gZIF-8-$mim_{0.15}im_{0.74}bim_{0.11}$. **b** ZIF-8, ZIF-8-$mim_{0.15}im_{0.85}$, and ZIF-61-$mim_{0.15}im_{0.85}$. **c** ZIF-67, ZIF-67-$mim_{0.18}im_{0.68}bim_{0.14}$, and a_gZIF-67-$mim_{0.18}im_{0.68}bim_{0.14}$.

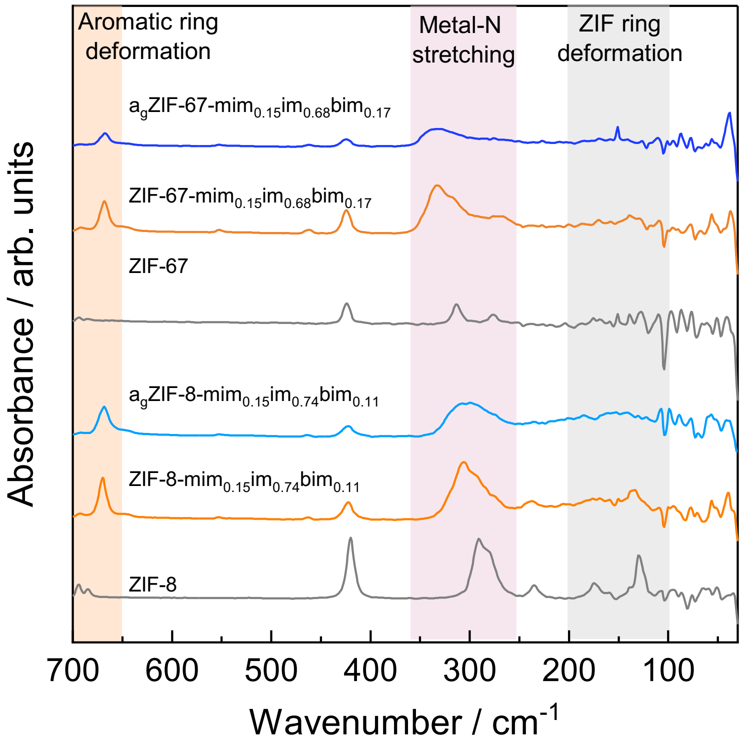

Supplementary Figure 62. Comparison of far-IR spectra data of ZIF-8 derivatives with ZIF-67 derivatives in the crystalline and glassy states.

Supplementary Methods 6 – Thermal analysis

Supplementary Methods 6.1 – TGA and DSC

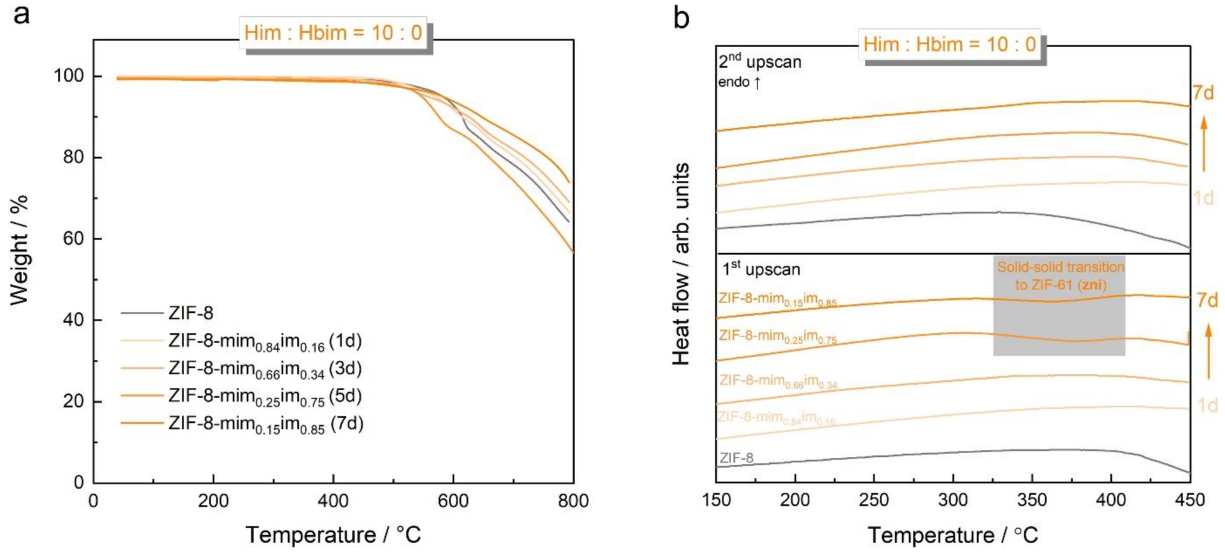

Supplementary Figure 63. a TGA data collected for ZIF-8 derivatives after varying SALE reaction times with a solution containing only Him. **b** DSC data of the same samples showing two consecutive upscans. A weak and broad exothermic signal is visible on the first upscan for the samples containing large im$^-$ concentrations. These signal is assigned to the solid-solid transition of the sample from the **sod** phase (ZIF-8) to the **zni** phase (ZIF-61).

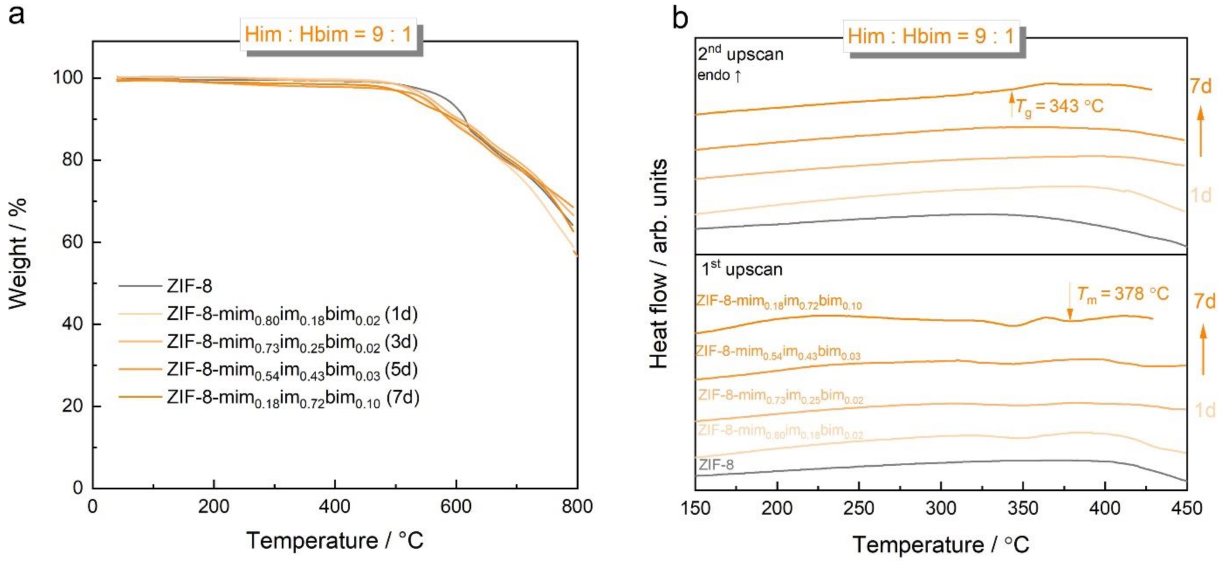

Supplementary Figure 64. a TGA data collected for ZIF-8 derivatives after varying SALE reaction times with a solution containing Him and Hbim in a molar ratio of 9:1. **b** DSC data of the same samples showing two consecutive upscans. An exothermic signal followed by an endothermic signal is visible in the first upscan for the sample containing a large im$^-$ concentration and a small bim$^-$ concentration. The exothermic signal is attributed to the collapse of the frameworks and the endothermic signal is assigned to melting (T_m). A similar thermal behaviour has previously been observed for the liquid- and glass-forming ZIF-4 as well as some functionalized derivatives of ZIF-4.[3,4] The material that undergoes melting in the first upscan shows a clear glass transition signal (T_g) in the second upscan.

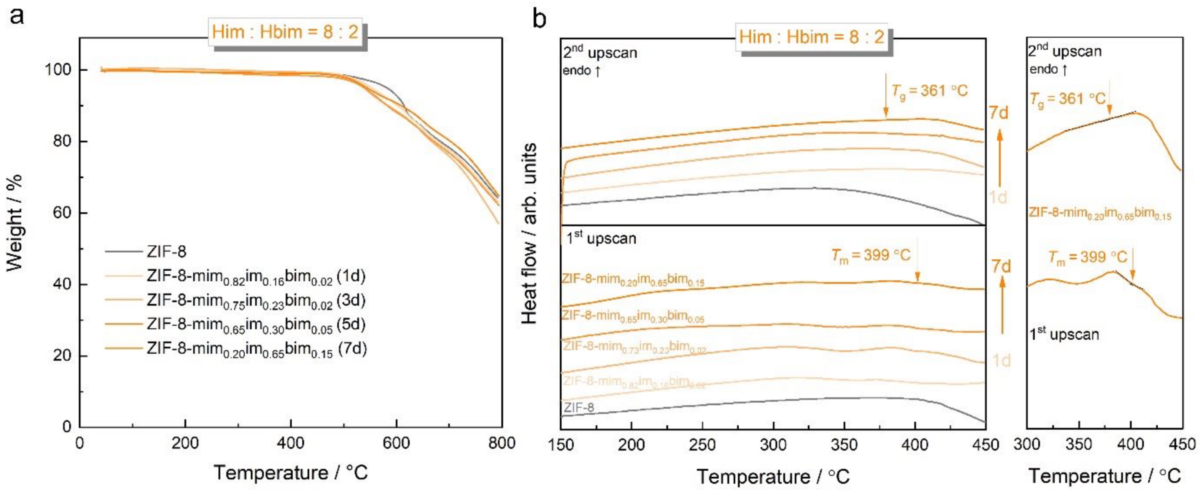

Supplementary Figure 65. a TGA data collected for ZIF-8 derivatives after varying SALE reaction times with a solution containing Him and Hbim in a molar ratio of 8:2. **b** DSC data of the same samples showing two consecutive upscans. The zoomed-in curves on the right are used to demonstrate the determination of T_m and T_g. An exothermic signal followed by an endothermic signal is visible in the first upscan for the sample containing a large im$^-$ concentration and small bim$^-$ concentration. The exothermic signal is attributed to the collapse of the frameworks and the endothermic signal is assigned to melting (T_m). A similar thermal behaviour has previously been observed for the liquid- and glass-forming ZIF-4 as well as some functionalized derivatives of ZIF-4.[3,4] The material that undergoes melting in the first upscan displays a signal which can be assigned to the glass transition (T_g) in the second upscan.

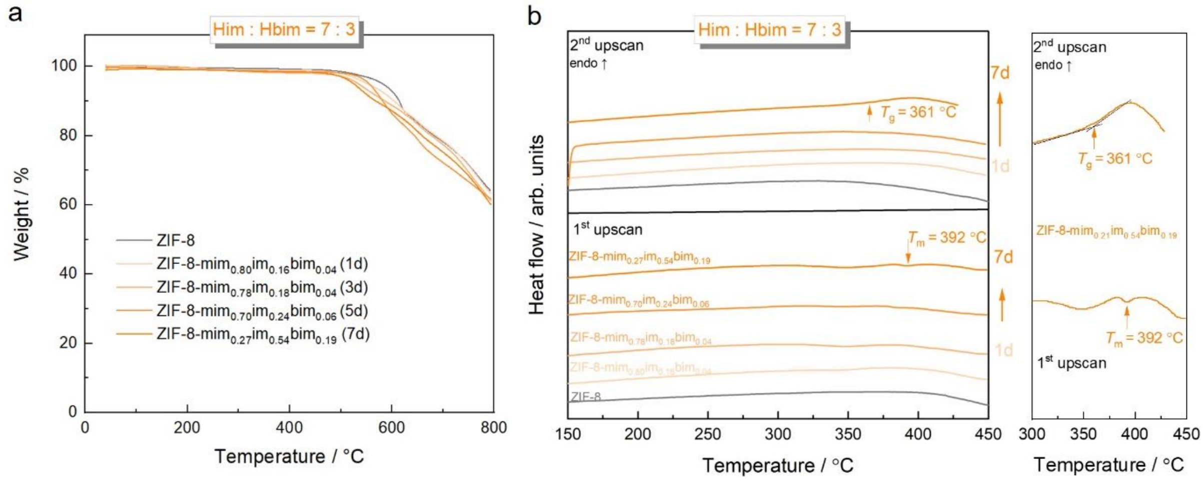

Supplementary Figure 66. a TGA data collected for ZIF-8 derivatives after varying SALE reaction times with a solution containing Him and Hbim in a molar ratio of 7:3. **b** DSC data of the same samples showing two consecutive upscans. The zoomed-in curves on the right are used to demonstrate the determination of T_m and T_g The exothermic signal is attributed to the collapse of the frameworks and the endothermic signal is assigned to melting (T_m). A similar thermal behaviour has previously been observed for the liquid- and glass-forming ZIF-4 as well as some functionalized derivatives of ZIF-4.[3,4] The material that undergoes melting in the first upscan displays a signal which can be assigned to the glass transition (T_g) in the second upscan.

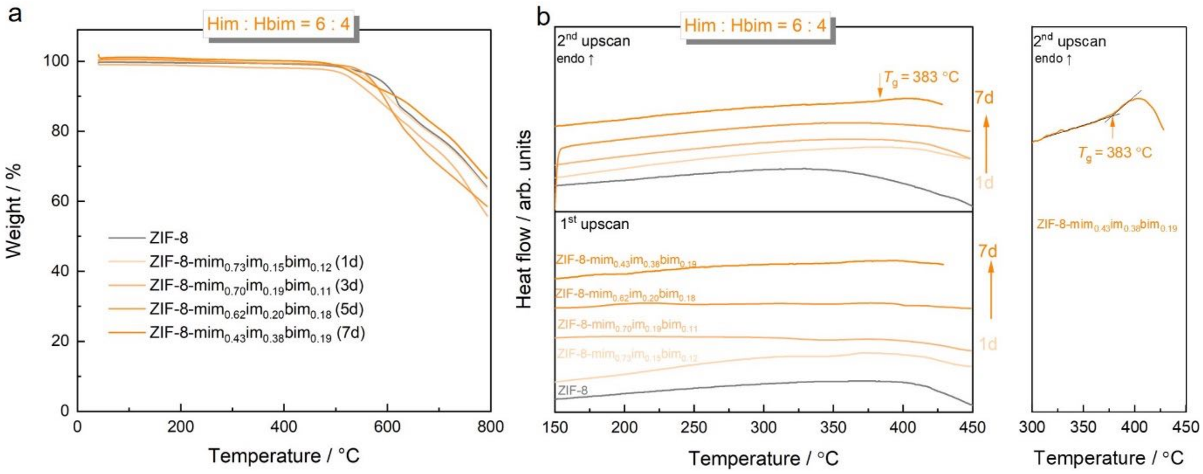

Supplementary Figure 67. a TGA data collected for ZIF-8 derivatives after varying SALE reaction times with a solution containing Him and Hbim in a molar ratio of 6:4. **b** DSC data of the same samples showing two consecutive upscans. The zoomed-in curve on the right is used to demonstrate the determination of T_g. ZIF-8-mim$_{0.43}$im$_{0.38}$bim$_{0.19}$ forms a CGC during the first heating cycle. The formation of the CGC composite appears to be a thermoneutral process, however, glassy behaviour is proven by a glass transition signal (T_g) in the second upscan.

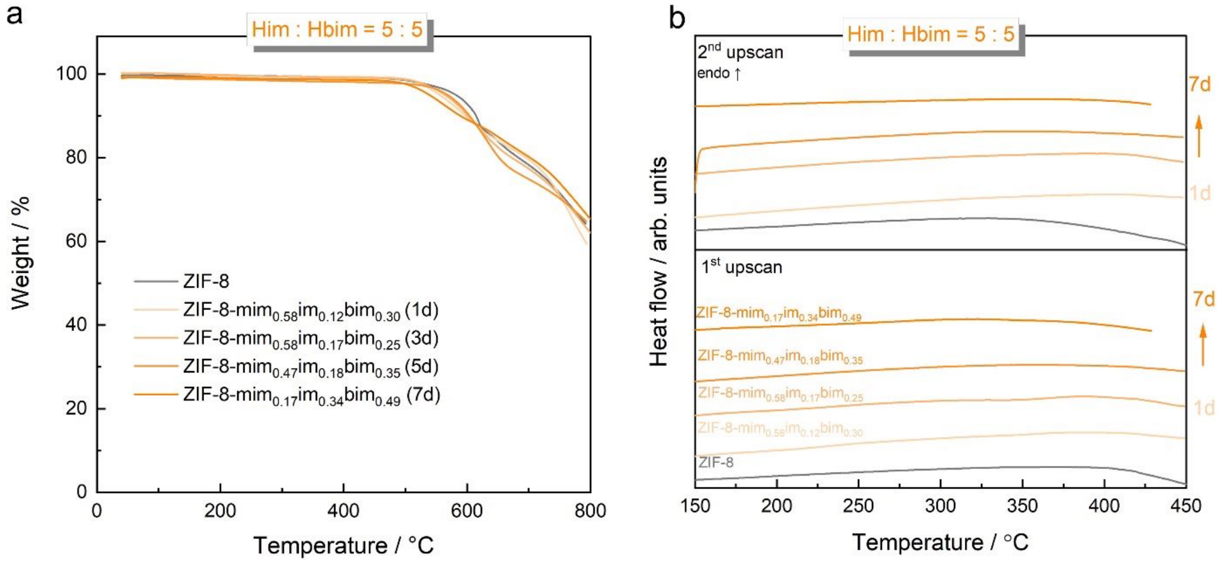

Supplementary Figure 68. a TGA data collected for ZIF-8 derivatives after varying SALE reaction times with a solution containing Him and Hbim in a molar ratio of 5:5. **b** DSC data of the same samples showing two consecutive upscans.

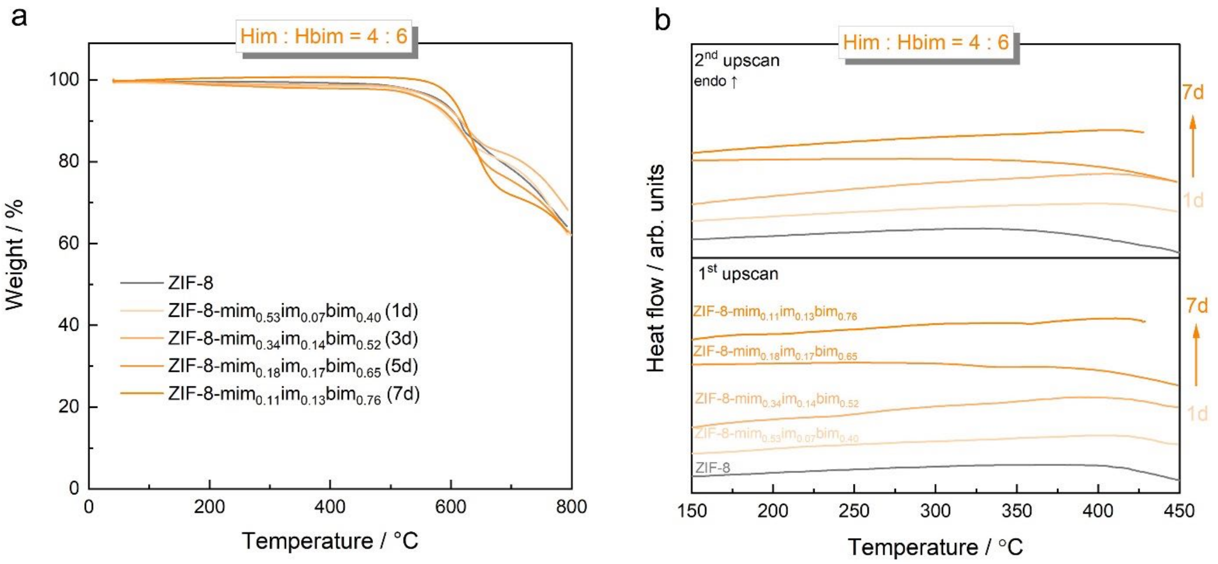

Supplementary Figure 69. a TGA data collected for ZIF-8 derivatives after varying SALE reaction times with a solution containing Him and Hbim in a molar ratio of 4:6. **b** DSC data of the same samples showing two consecutive upscans.

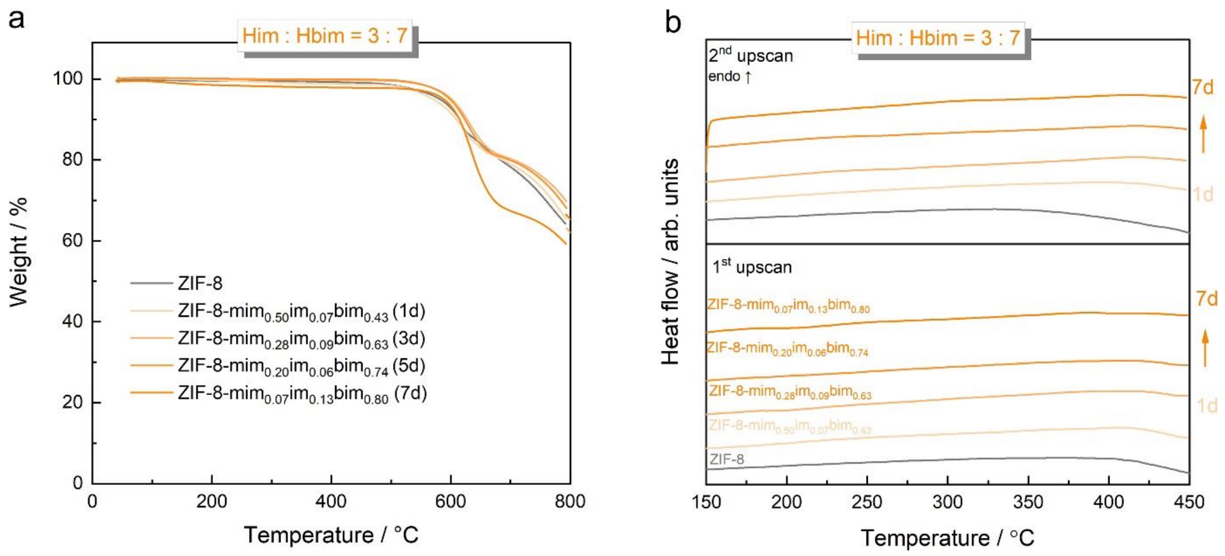

Supplementary Figure 70. a TGA data collected for ZIF-8 derivatives after varying SALE reaction times with a solution containing Him and Hbim in a molar ratio of 3:7. **b** DSC data of the same samples showing two consecutive upscans.

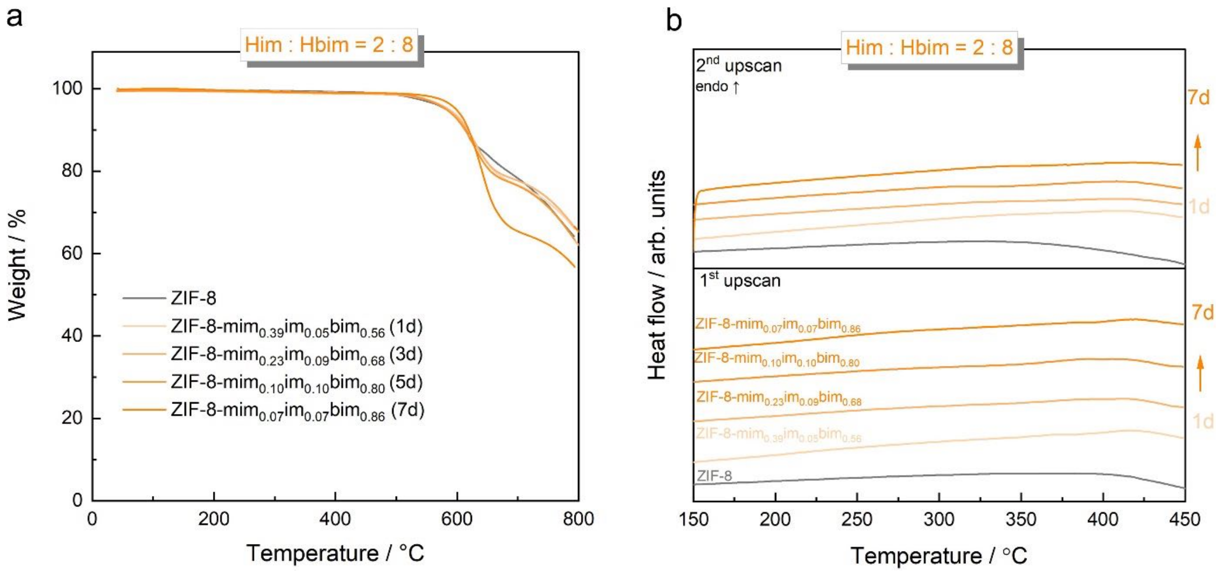

Supplementary Figure 71. a TGA data collected for ZIF-8 derivatives after varying SALE reaction times with a solution containing Him and Hbim in a molar ratio of 2:8. **b** DSC data of the same samples showing two consecutive upscans.

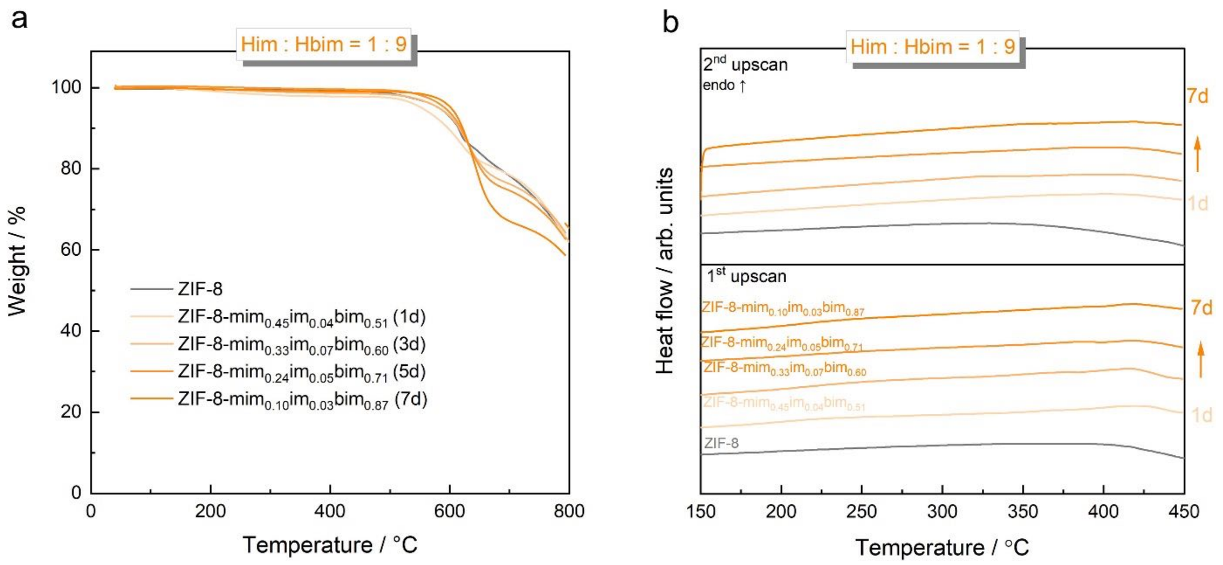

Supplementary Figure 72. a TGA data collected for ZIF-8 derivatives after varying SALE reaction times with a solution containing Him and Hbim in a molar ratio of 1:9. **b** DSC data of the same samples showing two consecutive upscans.

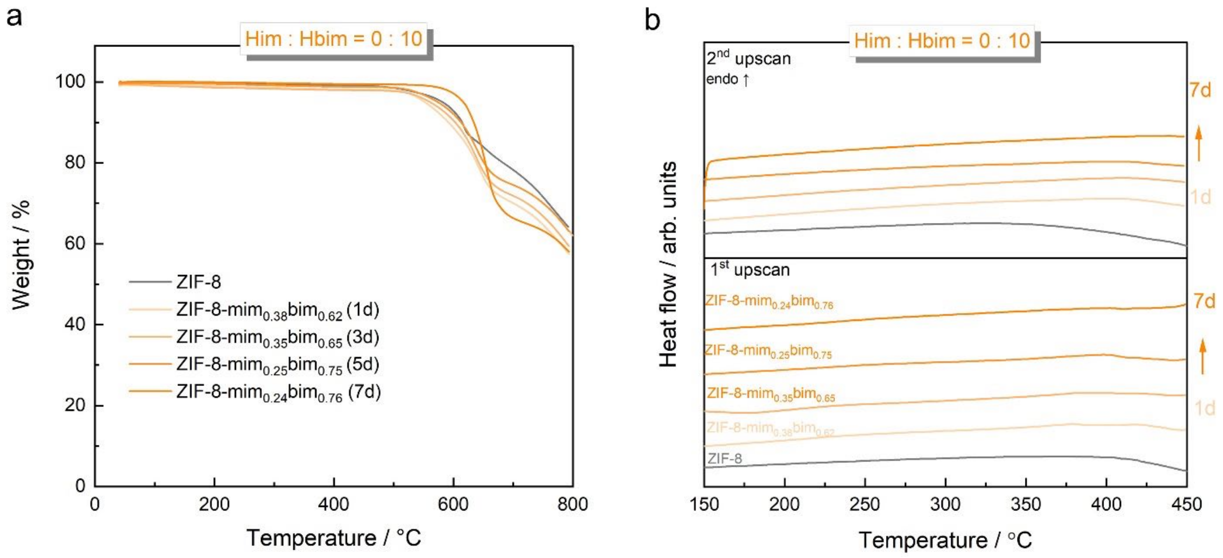

Supplementary Figure 73. a TGA data collected for ZIF-8 derivatives after varying SALE reaction times with a solution containing only Hbim. **b** DSC data of the same samples showing two consecutive upscans.

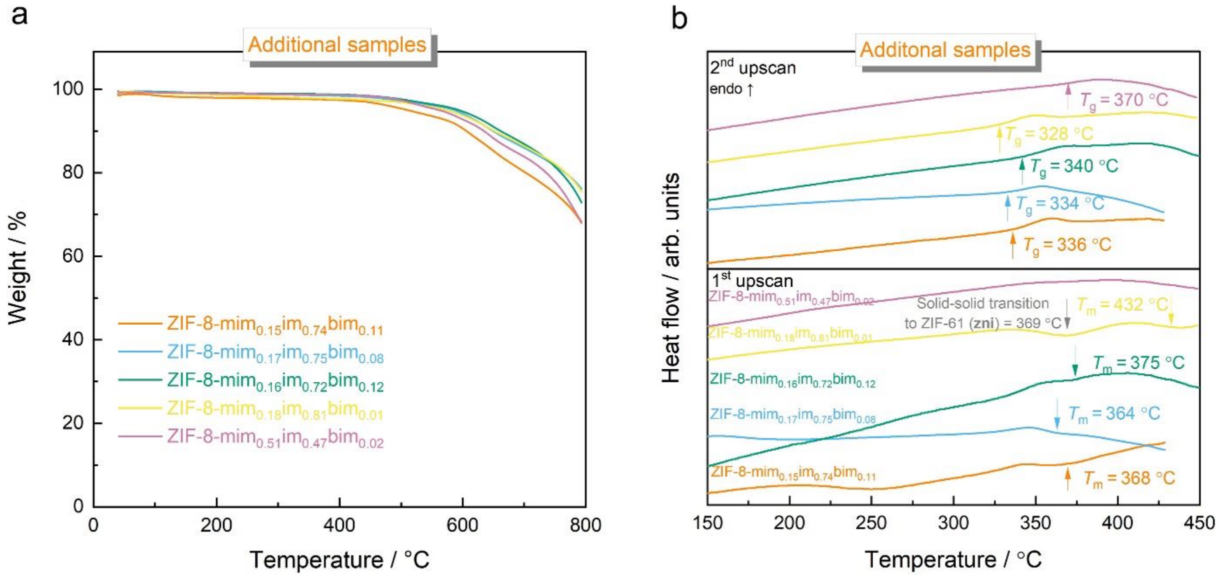

Supplementary Figure 74. a TGA for ZIF-8 derivatives made from a more refined applied exchange ratio of imidazole and benzimidazole. **b** DSC data of the same samples showing two consecutive upscans. An exothermic signal is visible on the first upscan for the sample containing large im⁻ concentrations and the smallest bim⁻ concentrations. This signal is assigned to the solid-solid transition of parts of the sample from the **sod** phase (ZIF-8) to the **zni** phase (ZIF-61). In addition, endothermic signals are visible on the first upscan for the samples containing large im⁻ concentrations and small bim⁻ concentrations. These signals are assigned to melting (T_m). For all materials, a glass transition signal (T_g) is observed in the second scan.

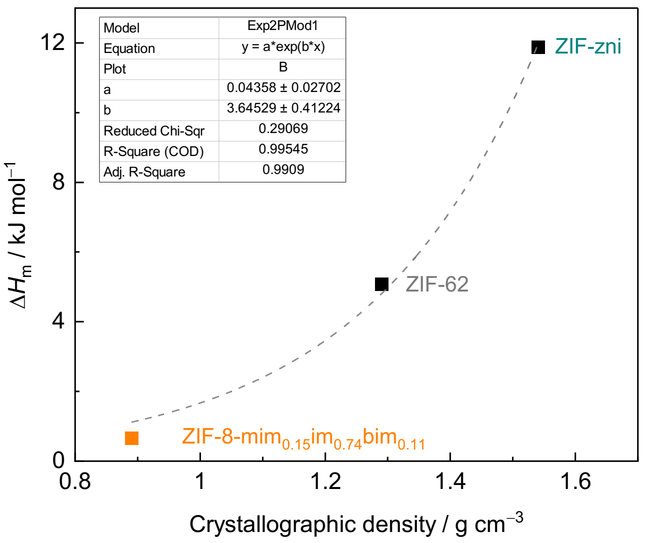

Supplementary Figure 75. Plot of crystallographic density against the molar melting enthalpy (ΔH_m) of ZIF-8-mim$_{0.15}$im$_{0.74}$bim$_{0.11}$, ZIF-62 and ZIF-zni. ΔH_m of ZIF-8-mim$_{0.15}$im$_{0.74}$bim$_{0.11}$ was determined from the integral of the melting signal in the first DSC upscan. For the crystallographic density see Supplementary Table 4. The enthalpies and crystallographic densities of ZIF-62 and ZIF-zni were taken from reference[5]. The dashed curve represents an exponential fit to the data and is provided as a guide to the eye.

Supplementary Table 3. Comparison of thermodynamic melting parameters of ZIF-8-mim$_{0.15}$im$_{0.74}$bim$_{0.11}$ with other reported meltable ZIFs.

Material	Composition	T_m (°C)	T_m (K)	ΔH_m (kJ mol^{-1})	ΔS_m (J K^{-1} mol^{-1})	References
ZIF-8-mim$_{0.15}$im$_{0.74}$bim$_{0.11}$	Zn(mim)$_{0.30}$(im)$_{1.48}$(bim)$_{0.22}$	368	641	0.66	1.03	This work
ZIF-62	Zn(im)$_{1.75}$(bim)$_{0.25}$	435	708	2.92	4.12	*Sci. Adv.* 4, eaao6827 (2018)
ZIF-62-bim$_{0.35}$	Zn(im)$_{1.65}$(bim)$_{0.35}$	438	711	5.08	7.14	*Nat. Commun.* 13, 7750 (2022)
ZIF-4	Zn(im)$_2$	579	852	11.46	13.45	*Nat. Commun.* 13, 7750 (2022)
ZIF-zni	Zn(im)$_2$	585	858	11.88	13.85	*Nat. Commun.* 13, 7750 (2022)
TIF-4	Zn(im)$_{1.68}$(mbim)$_{0.32}$	436	709	2.69	3.79	Nat. Commun. 13, 7750 (2022)
ZIF-UC-5	Zn(im)$_{1.80}$(Clbim)$_{0.20}$	428	701	2.50	3.60	*CrystEngComm*, ,22, 3627-3637 (2020)
ZIF-UC-6	Zn(im)$_{1.82}$(abim)$_{0.18}$	345	618	1.70	2.80	*Chem. Mater.* 34, 2187–2196 (2022)

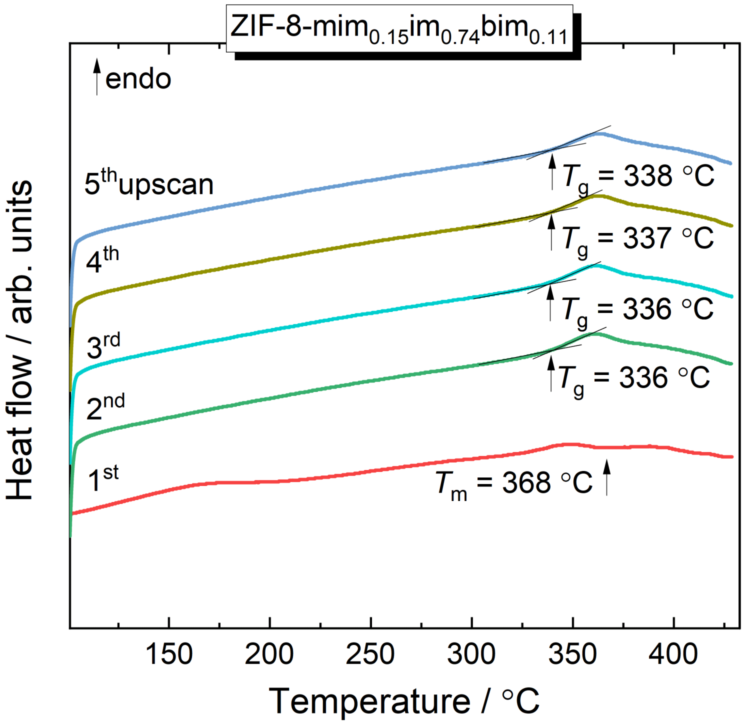

Supplementary Figure 76. Five consecutive DSC cycles for ZIF-8-$mim_{0.15}im_{0.74}bim_{0.11}$ showcasing the stability of T_g.

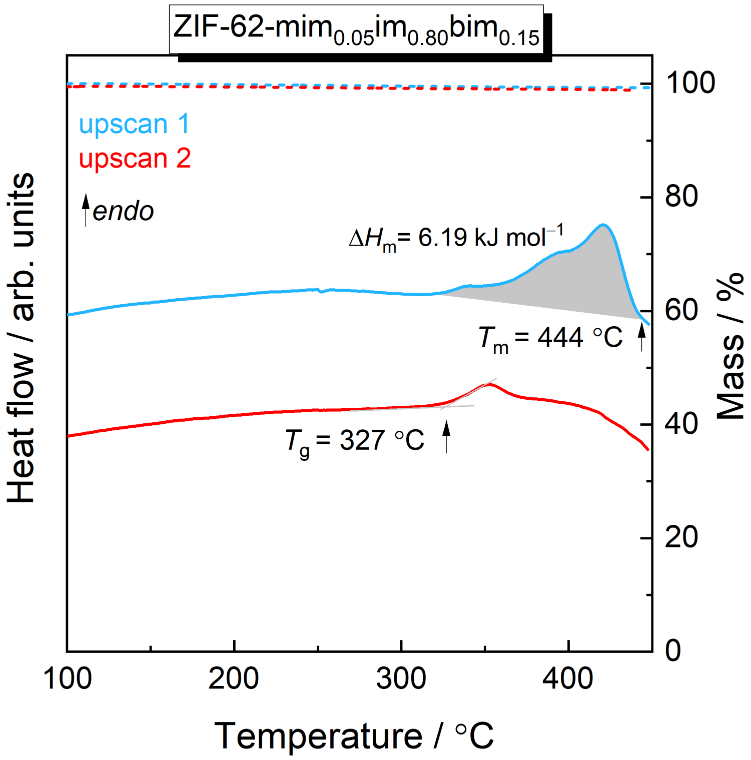

Supplementary Figure 77. TGA (dotted lines) and DSC (solid lines) for ZIF-62-$mim_{0.05}im_{0.80}bim_{0.15}$. The sample was cycled from 50 °C to 450 °C with a heating/cooling rate of ±10 °C/min for two times. The sample is obtained by a SALE process from ZIF-8 in which the reaction mixture was stirred for 15 min/day (total reaction time is 3 days).

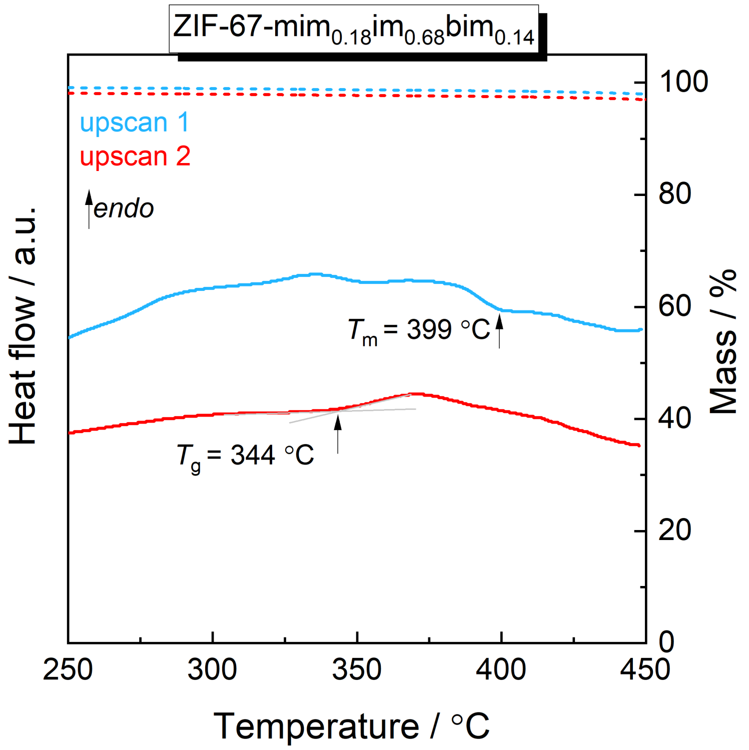

Supplementary Figure 78. TGA (dotted lines) and DSC (solid lines) for ZIF-67-$mim_{0.18}im_{0.68}bim_{0.14}$. The sample was cycled from 50 °C to 450 °C with a heating/cooling rate of ±10 °C/min for two times.

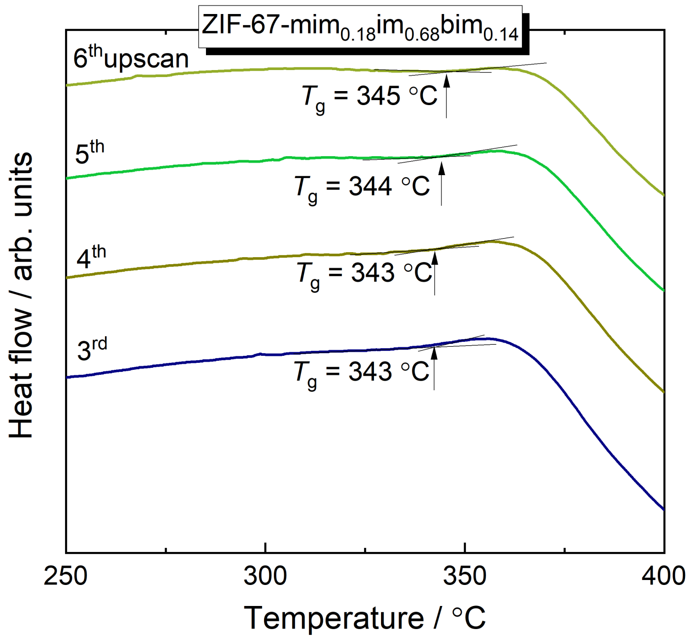

Supplementary Figure 79. The third to sixth consecutive DSC cycles for ZIF-67-$mim_{0.18}im_{0.68}bim_{0.14}$ showing the stability of T_g.

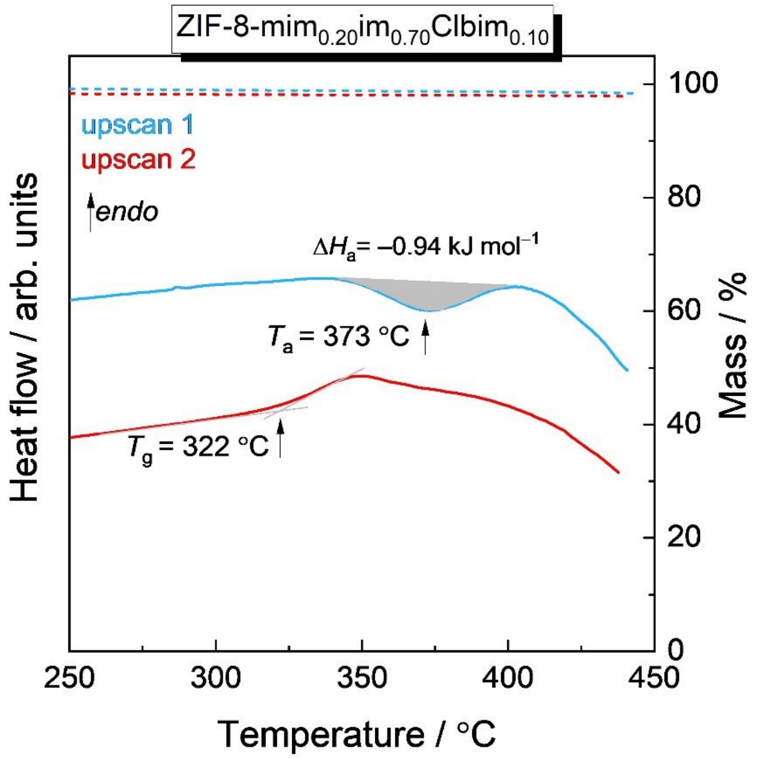

Supplementary Figure 80. TGA (dotted lines) and DSC (solid lines) for ZIF-8-$mim_{0.20}im_{0.70}Clbim_{0.10}$. The sample was cycled from 50 °C to 440 °C with a heating/cooling rate of ±10 °C/min for two times. The peak in the first upscan is defined as the amorphisation temperature (T_a) due to its exothermic nature. The fact that a glass transition is observable at 322 °C in the second upscan suggests that the amorphization involves a slightly exothermic transition to a liquid phase. Other slightly exothermic transitions to ZIF liquids were previously reported for ZIF-4 and some of its functionalized derivatives.[3,4]

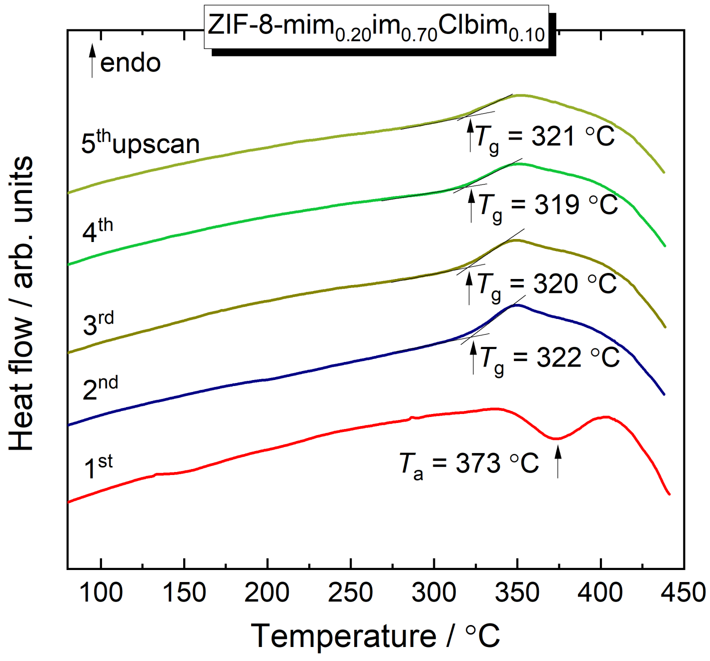

Supplementary Figure 81. Five consecutive DSC cycles for ZIF-8-$mim_{0.20}im_{0.70}Clbim_{0.10}$ showcasing the stability of T_g.

Supplementary Table 4. Summary of the transition temperatures determined from the DSC data.

Material	T_{rc} / °C	T_m / °C	T_g / °C	Material after heat treatment
ZIF-8-$mim_{0.25}im_{0.75}$	363	/	/	ZIF-61-$mim_{0.25}im_{0.75}$
ZIF-8-$mim_{0.15}im_{0.85}$	374	/	/	ZIF-61-$mim_{0.15}im_{0.85}$
ZIF-8-$mim_{0.18}im_{0.72}bim_{0.10}$	/	378	343	a_gZIF-8-$mim_{0.18}im_{0.72}bim_{0.10}$
ZIF-8-$mim_{0.20}im_{0.65}bim_{0.15}$	/	399	361	a_gZIF-8-$mim_{0.20}im_{0.65}bim_{0.15}$
ZIF-8-$mim_{0.27}im_{0.54}bim_{0.19}$	/	392	361	a_gZIF-8-$mim_{0.27}im_{0.54}bim_{0.19}$
ZIF-8-$mim_{0.43}im_{0.38}bim_{0.19}$	/	/	383	ZIF-8/a_gZIF-8-$mim_{0.43}im_{0.38}bim_{0.19}$ CGC
ZIF-8-$mim_{0.15}im_{0.74}bim_{0.11}$	/	368	336	a_gZIF-8-$mim_{0.15}im_{0.74}bim_{0.11}$
ZIF-8-$mim_{0.17}im_{0.75}bim_{0.08}$	/	360	334	a_gZIF-8-$mim_{0.17}im_{0.75}bim_{0.08}$
ZIF-8-$mim_{0.16}im_{0.72}bim_{0.12}$	/	375	340	a_gZIF-8-$mim_{0.16}im_{0.72}bim_{0.12}$
ZIF-8-$mim_{0.18}im_{0.81}bim_{0.01}$	369	432	328	ZIF-61/a_gZIF-8-$mim_{0.18}im_{0.81}bim_{0.01}$ CGC
ZIF-8-$mim_{0.51}im_{0.47}bim_{0.02}$	/	/	370	ZIF-8/a_gZIF-8-$mim_{0.51}im_{0.47}bim_{0.02}$ CGC
ZIF-62-$mim_{0.05}im_{0.80}bim_{0.15}$	/	444	327	a_gZIF-62-$mim_{0.05}im_{0.80}bim_{0.15}$
ZIF-8-$mim_{0.20}im_{0.70}Clbim_{0.10}$	/	373[a]	322	a_gZIF-8-$mim_{0.20}im_{0.70}Clbim_{0.10}$
ZIF-67-$mim_{0.18}im_{0.68}bim_{0.14}$	/	399	344	ZIF-67-$mim_{0.18}im_{0.68}bim_{0.14}$

[a] defined as the amorphisation temperature (T_a) due to the exothermic transition into a (supercooled) liquid state.

Supplementary Methods 6.2 – Heat capacity measurements

The evolution of heat capacity (C_p) of a_gZIF-8-mim$_{0.15}$im$_{0.74}$bim$_{0.11}$ in the range from 200 to 415 °C was determined by modulated DSC using a DSC 25 calorimeter (TA Instruments). In this measurement, a sinusoidal modulation with a temperature amplitude of ±1 °C and a modulation period of 120 s was overlaid on a linear heating ramp with an average heating rate of 2 °C min^{-1}. Baseline and sapphire reference scans were collected before the sample scan using the same temperature program.

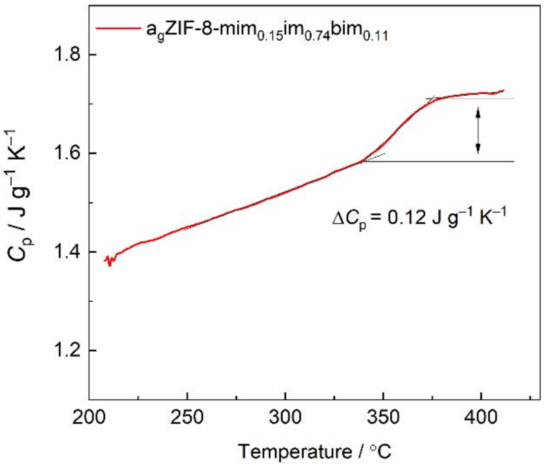

Supplementary Figure 82. Heat capacity (C_p) scan of a_gZIF-8-mim$_{0.15}$im$_{0.74}$bim$_{0.11}$. The heat capacity change around the glass transition (ΔC_p) was determined using the difference between the two intersections of the onset and offset tangent lines of the glass transition signal.

Supplementary Table 5. Comparison of the heat capacity change (ΔC_p) around the glass transition of a_gZIF-8-mim$_{0.15}$im$_{0.74}$bim$_{0.11}$ and other reported ZIF glasses.

Material	Composition	T_g (°C)	ΔC_p (J g^{-1} K^{-1})	References
a_gZIF-8-mim$_{0.15}$im$_{0.74}$bim$_{0.11}$	Zn(mim)$_{0.30}$(im)$_{1.48}$(bim)$_{0.22}$	336	0.12	This work
a_gZIF-62	Zn(im)$_{1.75}$(bim)$_{0.25}$	322	0.19	*Sci. Adv.* 4, eaao6827 (2018)
a_g(IL@ZIF-8)	[EMIM][TFSI]@Zn(mim)$_2$	322	0.11	*Nat. Commun.* 12, 5703 (2021)
a_gZIF-4 (HDA)	Zn(im)$_2$	292	0.16	*Nat. Commun.* 6, 8079 (2015)
a_gZIF-4 (LDA)		316	0.11	

Supplementary Methods 6.3 – Determination of the calorimetric fragility

The fragility of the a_gZIF-8-$mim_{0.15}im_{0.74}bim_{0.11}$ was determined according to procedures published in previous work[6]. The melt-quenched ZIF glass was cycled from 50 °C to 430 °C, with varying heating and cooling rates from ±25 to ±5 °C/min. T_f is the fictive temperature of the glass sample prepared with different cooling rates (q_c). T_f at a heating/cooling rate of ±10 °C/min corresponds to T_g. The calorimetric fragility index m is determined as the slope of the plot of $\log_{10}(1/q_c)$ versus T_g/T_f (i.e. the T_g scaled reciprocal T_f, absolute temperatures in Kelvin).

$$m = \frac{\partial \log_{10}\left(\frac{1}{q_c}\right)}{\partial\left(\frac{T_g}{T_f}\right)} \qquad (1)$$

The fragility index m is a measure for the activation energy of viscous flow at T_g. Glasses with a low fragility are called strong (for example vitreous silica with m = 20) while glasses with a fragility higher than about 40 are called fragile.

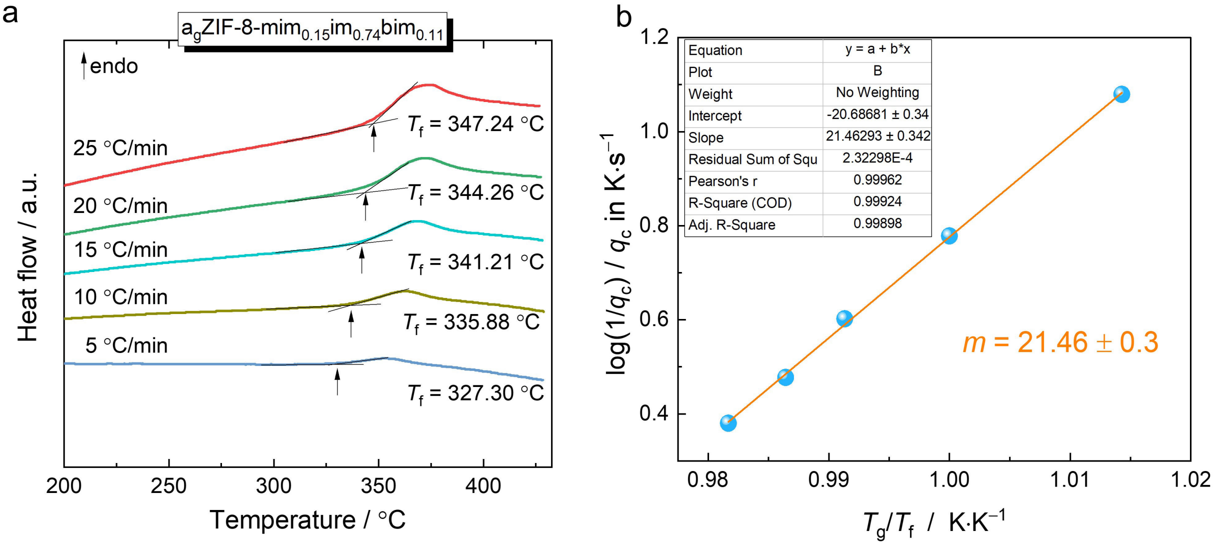

Supplementary Figure 83. a DSC upscans of a_gZIF-8-$mim_{0.15}im_{0.74}bim_{0.11}$ with heating rates from 25 to 5 °C/min. The cooling rate before each of these scans has been identical to its heating rate. **b** Determination of the calorimetric fragility index m by a linear fit of $\log(1/q_c)$ against T_g/T_f.

Supplementary Methods 7 – Micrography

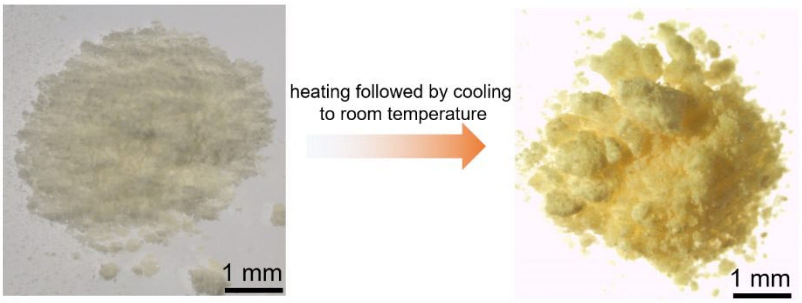

Supplementary Figure 84. Micrographs of ZIF-8 obtained before and after two upscans in the DSC instrument (maximum temperature reached was 450 °C).

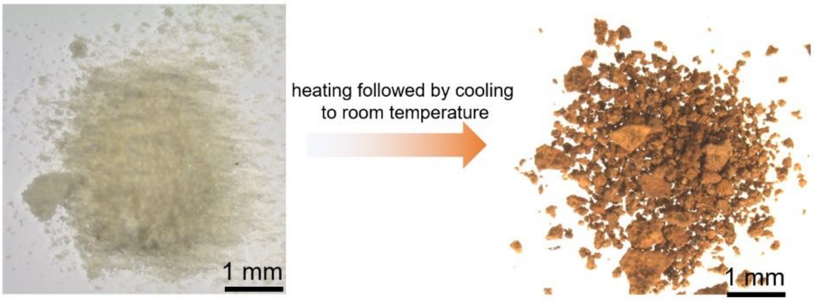

Supplementary Figure 85. Micrographs of ZIF-8-mim$_{0.15}$im$_{0.85}$ obtained before and after two upscans in the DSC instrument (maximum temperature reached was 450 °C).

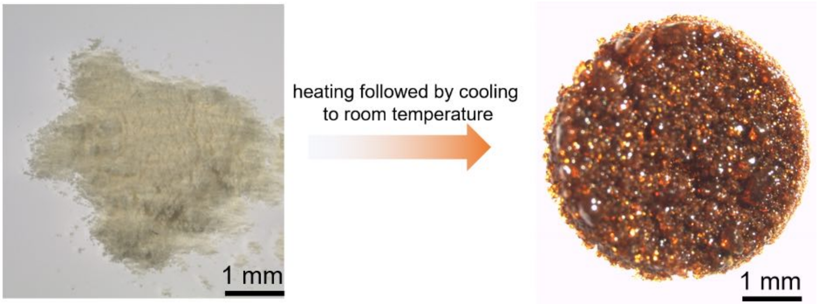

Supplementary Figure 86. Micrographs of ZIF-8-mim$_{0.15}$im$_{0.74}$bim$_{0.11}$ obtained before and after the preparative TGA/DSC experiment (maximum temperature reached was 430 °C).

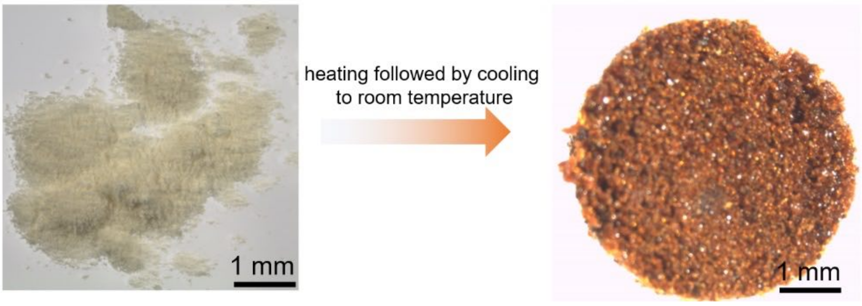

Supplementary Figure 87. Micrographs of ZIF-8-mim$_{0.18}$im$_{0.72}$bim$_{0.10}$ obtained before and after two upscans of DSC experiment (maximum temperature reached was 430 °C).

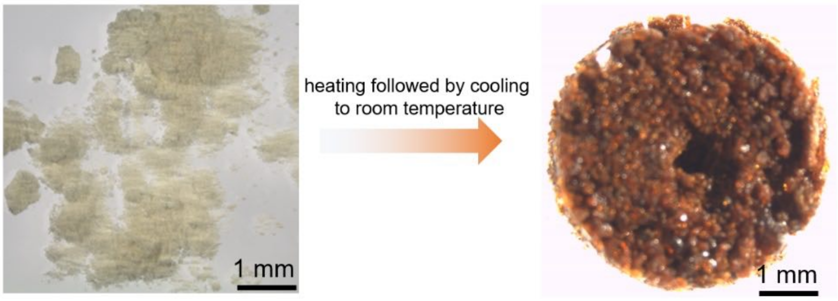

Supplementary Figure 88. Micrographs of ZIF-8-mim$_{0.20}$im$_{0.65}$bim$_{0.15}$ obtained before and after two upscans of DSC experiment (maximum temperature reached was 450 °C).

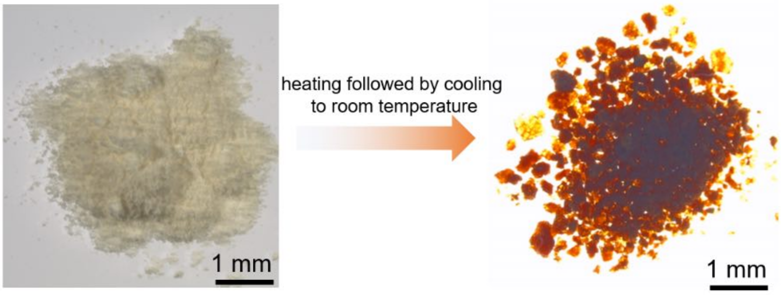

Supplementary Figure 89. Micrographs of ZIF-8-mim$_{0.27}$im$_{0.54}$bim$_{0.19}$ obtained before and after two upscans of DSC experiment (maximum temperature reached was 450 °C).

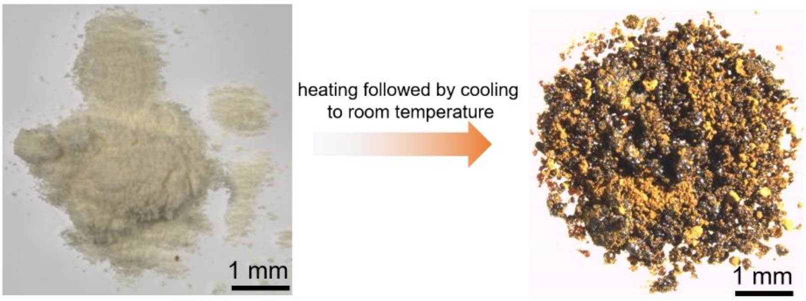

Supplementary Figure 90. Micrographs of ZIF-8-mim$_{0.43}$im$_{0.38}$bim$_{0.19}$ obtained before and after two upscans of DSC experiment (maximum temperature reached was 430 °C).

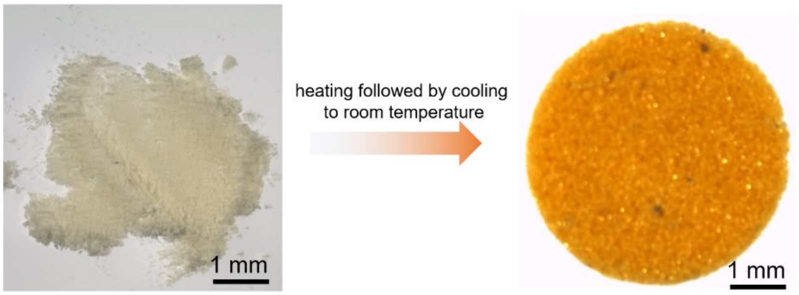

Supplementary Figure 91. Micrographs of ZIF-8-mim$_{0.17}$im$_{0.75}$bim$_{0.08}$ obtained before and after two upscans of DSC experiment (maximum temperature reached was 430 °C).

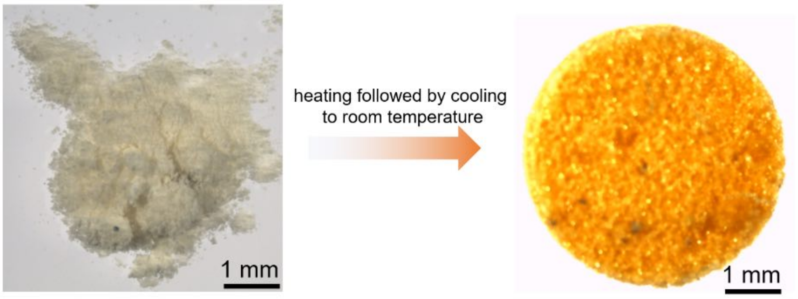

Supplementary Figure 92. Micrographs of ZIF-8-mim$_{0.16}$im$_{0.72}$bim$_{0.12}$ obtained before and after the preparative TGA/DSC experiment (maximum temperature reached was 450 °C).

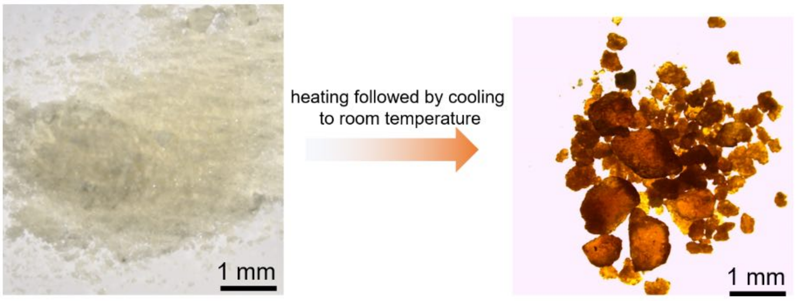

Supplementary Figure 93. Micrographs of ZIF-8-mim$_{0.18}$im$_{0.81}$bim$_{0.01}$ obtained before and after the preparative TGA/DSC experiment (maximum temperature reached was 450 °C).

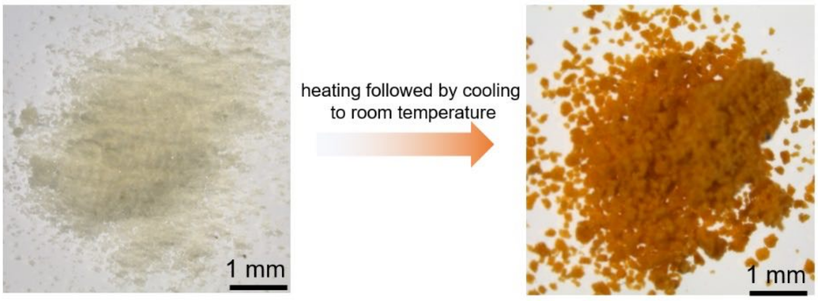

Supplementary Figure 94. Micrographs of ZIF-8-mim$_{0.51}$im$_{0.47}$bim$_{0.02}$ obtained before and after the preparative TGA/DSC experiment (maximum temperature reached was 450 °C).

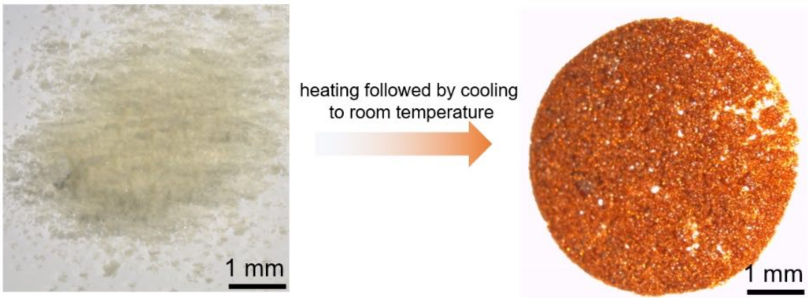

Supplementary Figure 95. Micrographs of ZIF-8-mim$_{0.20}$im$_{0.70}$Clbim$_{0.10}$ obtained before and after the preparative TGA/DSC experiment (maximum temperature reached was 440 °C).

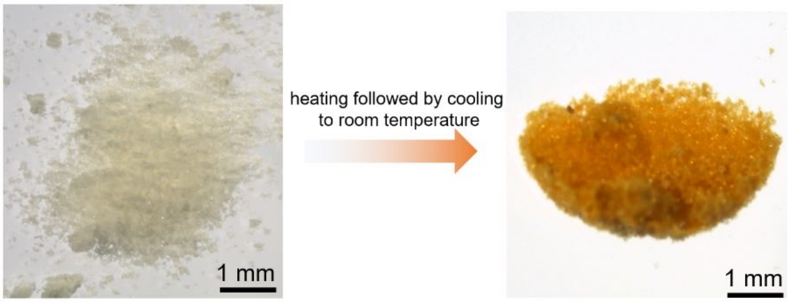

Supplementary Figure 96. Micrographs of ZIF-62-$mim_{0.05}im_{0.80}bim_{0.15}$ obtained before and after the preparative TGA/DSC experiment (maximum temperature reached was 450 °C).

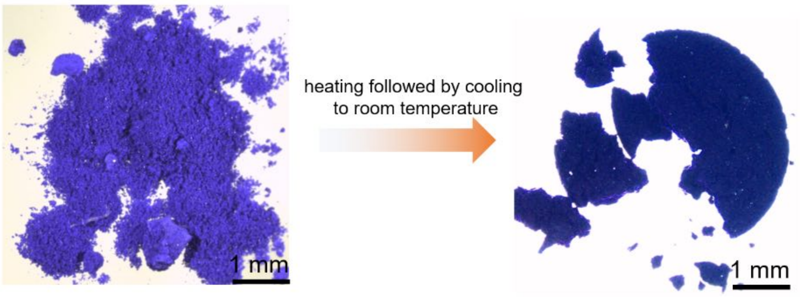

Supplementary Figure 97. Micrographs of ZIF-67-$mim_{0.18}im_{0.68}bim_{0.14}$ obtained before and after the preparative TGA/DSC experiment (maximum temperature reached was 450 °C).

Supplementary Methods 8 – Gas physisorption studies

Supplementary Methods 8.1 – Determination of pore volumes

The specific pore volumes (V_{pore}) were calculated according to previous work[5] with amendments:

$$V_{\text{pore}} = \frac{n_{\text{ads}}^{\text{max}} \cdot M_{\text{CO2}}}{\rho_{\text{sl}}} \qquad (2)$$

with $n_{\text{ads}}^{\text{max}}$ the specific molar amount of gas adsorbed (mmol of gas/g material) at 195 K and 95 kPa, M_{CO2} the molar mass of CO_2, and ρ_{sl} the density of the supercooled liquid at 195 K (that is 1.258 g cm^{-3}). For these calculations the implemented routine in the Quantachrome ASiQwin version 5.2 software was used. The obtained values are summarized in Supplementary Table 6.

Supplementary Table 6. Summary of maximum gas capacities ($V_{\text{ads}}^{\text{max}}$ and $n_{\text{ads}}^{\text{max}}$), specific pore volumes (V_{pore}) and BET surface area (S_{BET}) obtained from the CO_2 gas physisorption isotherms collected at 195 K or N_2 gas physisorption isotherms collected at 77 K.

**Sample**	Method	$V_{\text{ads}}^{\text{max}}$ (cm^3 g^{-1})	$n_{\text{ads}}^{\text{max}}$ (mmol g^{-1})	S_{BET} (m^2 g^{-1})	V_{pore} (cm^3 g^{-1})
ZIF-8	CO_2@195K	293.4	13.1	1434	0.46
	N_2@77K	410.9	18.3	1992	0.66
ZIF-8-$mim_{0.15}im_{0.74}bim_{0.11}$	CO_2@195K	293.0	13.1	989	0.46
	N_2@77K	303.7	13.6	1060	0.42
a_gZIF-8-$mim_{0.15}im_{0.74}bim_{0.11}$	CO_2@195K	125.0	5.6	403	0.20
	N_2@77K	108.4	4.8	229	0.16
ZIF-67-$mim_{0.18}im_{0.68}bim_{0.14}$	CO_2@195K	223.9	10.0	897	0.35
a_gZIF-67-$mim_{0.18}im_{0.68}bim_{0.14}$	CO_2@195K	118.5	5.3	316	0.18
a_gZIF-62[a]	CO_2@195K	74.9	3.4	200	0.12

[a] data taken from reference[5].

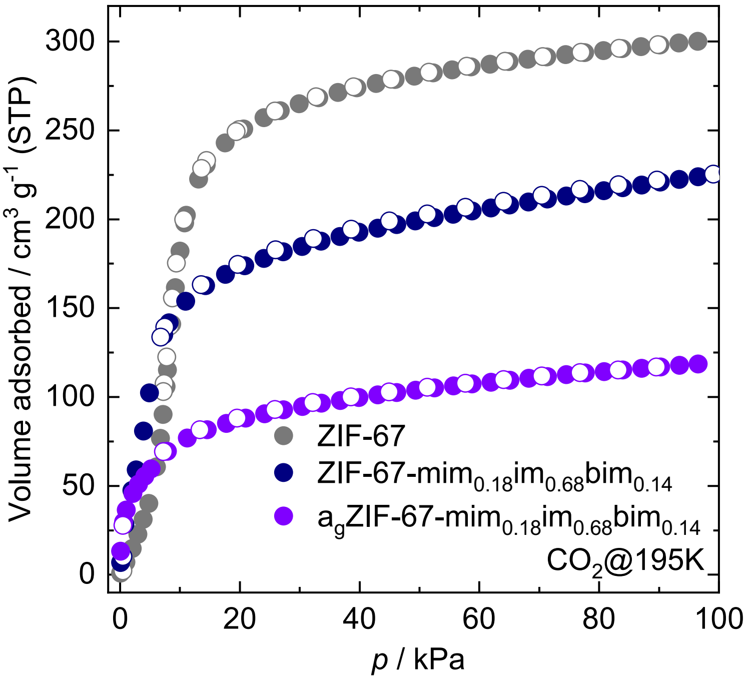

Supplementary Figure 98. CO_2 sorption isotherms recorded at 195 K for ZIF-67, ZIF-67-mim$_{0.18}$im$_{0.68}$bim$_{0.14}$ and a$_g$ZIF-67-mim$_{0.18}$im$_{0.68}$bim$_{0.14}$.

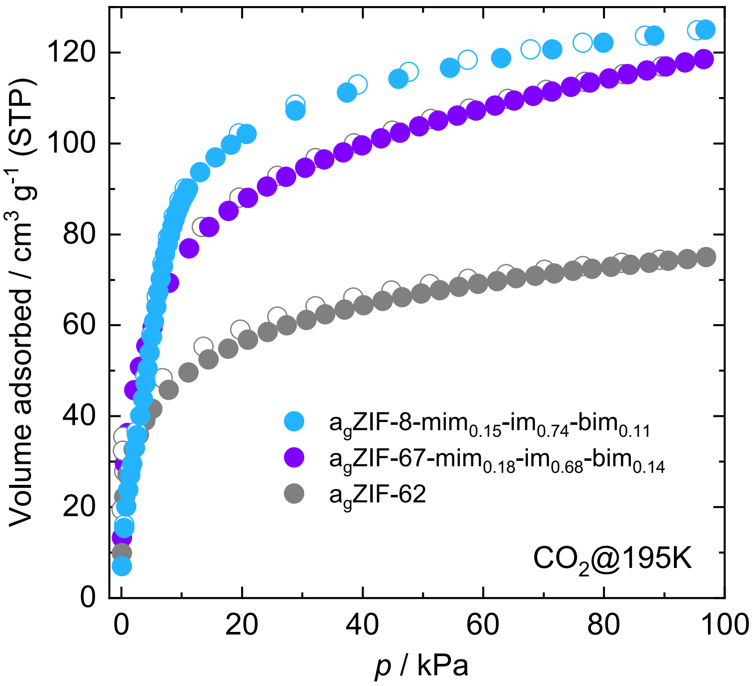

Supplementary Figure 99. CO_2 sorption isotherms recorded at 195 K for a$_g$ZIF-8-mim$_{0.15}$im$_{0.74}$bim$_{0.11}$, a$_g$ZIF-67-mim$_{0.18}$im$_{0.68}$bim$_{0.14}$ and a$_g$ZIF-62 (data taken from previous work reference[5]).

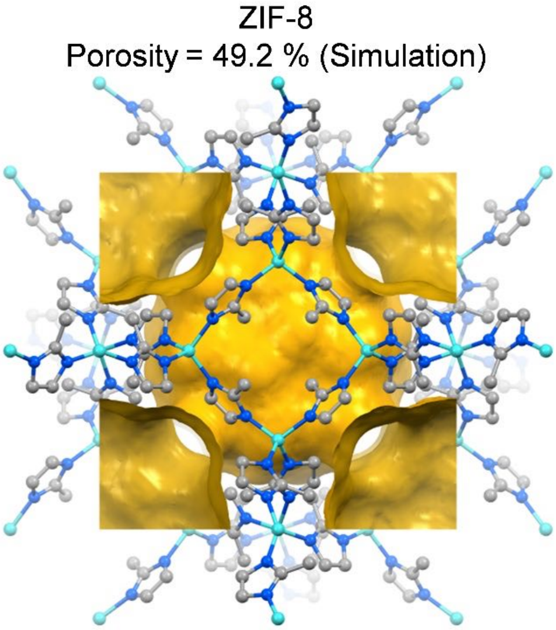

Supplementary Figure 100. Theoretical void fraction of ZIF-8 (CCDC code FAWCEN[7]) was calculated with a probe radius of 1.6 Å and a grid spacing of 0.2 Å by Mercury software V2021.3.0 and is shown in pale yellow. Structure viewed along the crystallographic *a* axis.

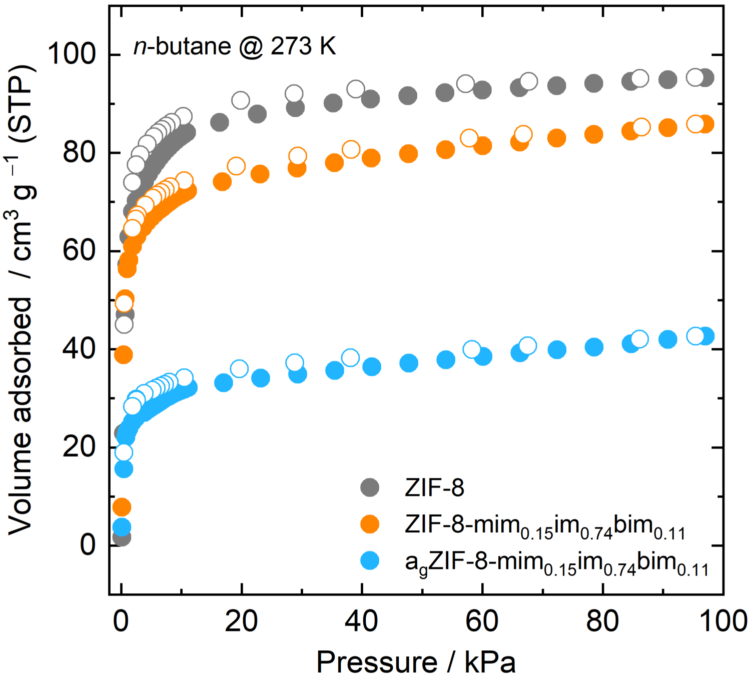

Supplementary Figure 101. *n*-butane sorption isotherms recorded at 273 K for ZIF-8, ZIF-8-$mim_{0.15}im_{0.74}bim_{0.11}$ and a_gZIF-8-$mim_{0.15}im_{0.74}bim_{0.11}$.

Supplementary Table 7. Comparison of the hydrocarbon gas capacities (propylene, propane, and *n*-butane) of a_gZIF-8-$mim_{0.15}im_{0.74}bim_{0.11}$ and a_gZIF-62.

Sample	V_{ads}^{max} (propylene) (cm^3 g^{-1})	V_{ads}^{max} (propane) (cm^3 g^{-1})	V_{ads}^{max} (*n*-butane) (cm^3 g^{-1})	
			273K	293K
a_gZIF-8-$mim_{0.15}im_{0.74}bim_{0.11}$	42.2	40.4	42.6	36.4
a_gZIF-62[a]	14.8	6.72	9.9	4.7

[a] data was taken from reference[5].

Supplementary Table 8. Comparison of the specific pore volumes (V_{pore}) of ZIF-8 derivatives obtained from gas sorption isotherms of *n*-butane (@273 K) and CO_2 (@195 K).

Sample	V_{pore} (CO_2)[a] (cm^3 g^{-1})	V_{pore} (*n*-butane)[b] (cm^3 g^{-1})	ratio
ZIF-8	0.46	0.41	1: 0.9
ZIF-8-$mim_{0.15}im_{0.74}bim_{0.11}$	0.46	0.41	1: 0.9
a_gZIF-8-$mim_{0.15}im_{0.74}bim_{0.11}$	0.20	0.18	1: 0.9

[a] $p \approx$ 95 kPa, applied value for the density of pure liquid adsorbate ρ_{liq}(CO_2 @195K) = 1.258 g cm^{-3}.[5]
[b] $p \approx$ 95 kPa, applied value for the density of pure liquid adsorbate ρ_{liq}(*n*-butane @273K) = 0.601 g cm^{-3}.[5]

Supplementary Methods 8.2 – Kinetic gas adsorption

To quantitatively analyze the kinetics for C_3H_6, C_3H_8, and n-C_4H_{10} adsorption in ZIF-8, ZIF-8-$mim_{0.15}im_{0.74}bim_{0.11}$ and a_gZIF-8-$mim_{0.15}im_{0.74}bim_{0.11}$, the time-dependent normalized uptake data were fitted using stretched exponential models[8] (pseudo-first-order does not fit the measured curves). The equation describing the relationship between the normalized uptake (corresponding to $(P_i - P)/(P_i - P_e)$) and time by a stretched exponential model is given as follows:

$$\text{normalized uptake} = \frac{(P_i - P)}{(P_i - P_e)} = 1 - e^{-(kt)^{\beta}} \tag{5}$$

where P_i is the initial pressure, P is the pressure at time t (in s), P_e is the pressure at equilibrium, k is the rate constant of gas adsorption (in s^{-1}), and β is the exponent parameter related to the width of the distribution of diffusion rate constants.

Supplementary Table 9. Kinetic parameters for C_3H_6, C_3H_8, and n-C_4H_{10} adsorption on ZIF-8, ZIF-8-$mim_{0.15}im_{0.74}bim_{0.11}$ and a_gZIF-8-$mim_{0.15}im_{0.74}bim_{0.11}$ at 293 K.

Sample	Adsorbate	k / s^{-1}	β	R^2
ZIF-8	propylene	3.26×10^{-2}	0.74	0.986
	propane	3.18×10^{-3}	0.39	0.991
	n-butane	3.06×10^{-3}	0.40	0.992
ZIF-8-$mim_{0.15}im_{0.74}bim_{0.11}$	propylene	2.14×10^{-2}	0.59	0.983
	propane	6.21×10^{-3}	0.42	0.996
	n-butane	4.48×10^{-3}	0.38	0.990
a_gZIF-8-$mim_{0.15}im_{0.74}bim_{0.11}$	propylene	9.37×10^{-3}	0.42	0.991
	propane	1.73×10^{-3}	0.49	0.996
	n-butane	1.49×10^{-3}	0.48	0.991

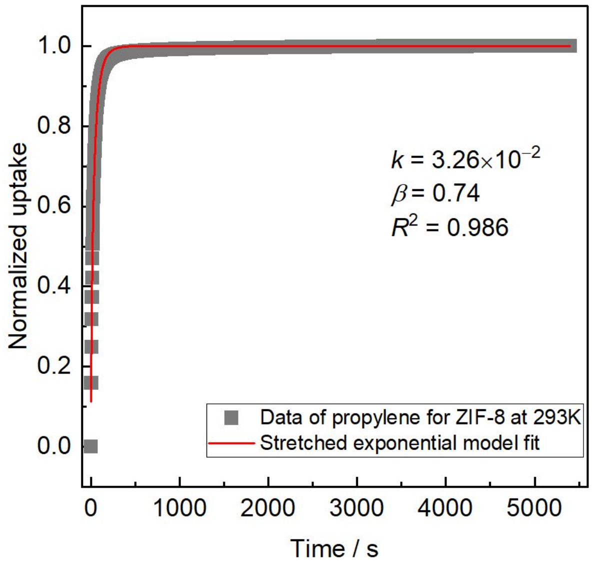

Supplementary Figure 102. Normalized uptake versus time for adsorption kinetic data of ZIF-8 for propylene at 293 K. Stretched exponential fit to the data is shown as a red line.

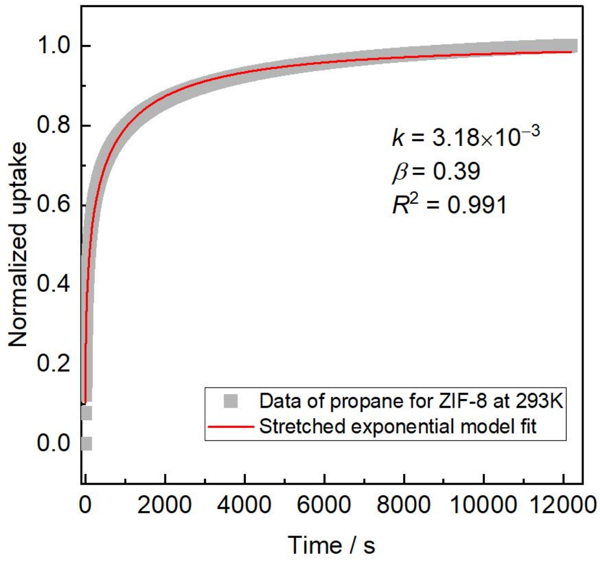

Supplementary Figure 103. Normalized uptake versus time for adsorption kinetic data of ZIF-8 for propane at 293 K. Stretched exponential fit to the data is shown as a red line.

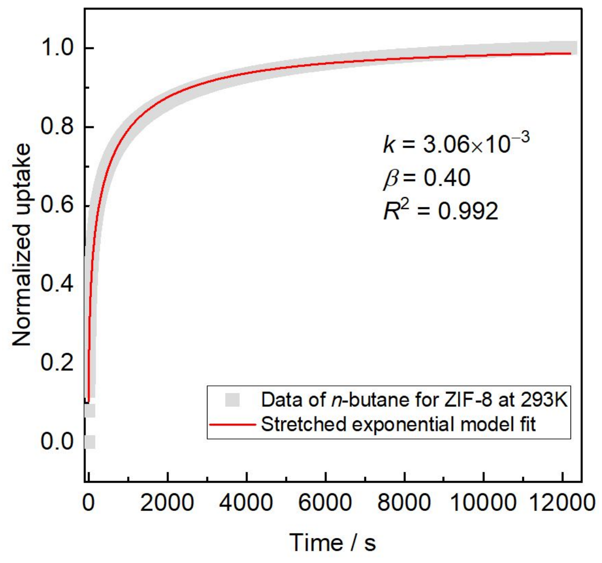

Supplementary Figure 104. Normalized uptake versus time for adsorption kinetic data of ZIF-8 for *n*-butane at 293 K. Stretched exponential fit to the data is shown as a red line.

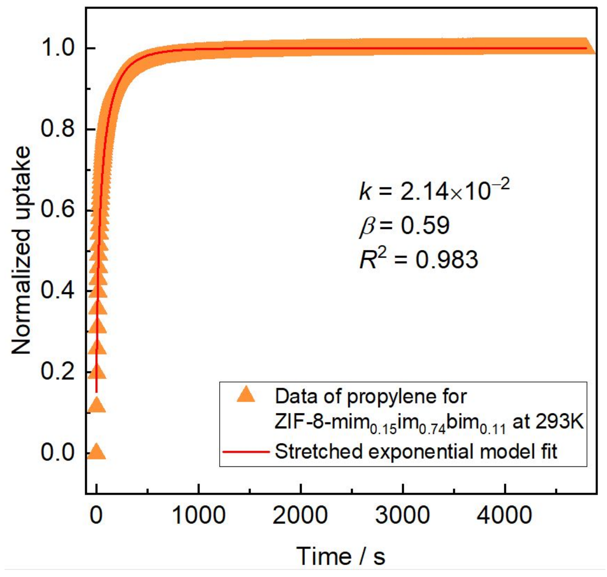

Supplementary Figure 105. Normalized uptake versus time for adsorption kinetic data of ZIF-8-$mim_{0.15}im_{0.74}bim_{0.11}$ for propylene at 293 K. Stretched exponential fit to the data is shown as a red line.

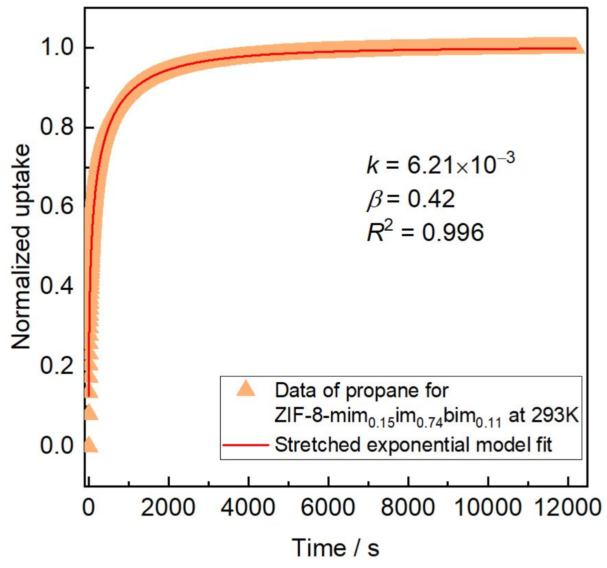

Supplementary Figure 106. Normalized uptake versus time for adsorption kinetic data of ZIF-8-mim$_{0.15}$im$_{0.74}$bim$_{0.11}$ for propane at 293 K. Stretched exponential fit to the data is shown as a red line.

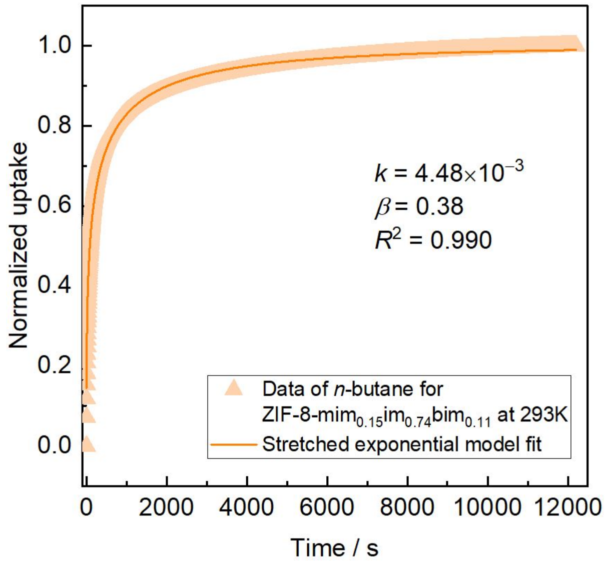

Supplementary Figure 107. Normalized uptake versus time for adsorption kinetic data of ZIF-8-mim$_{0.15}$im$_{0.74}$bim$_{0.11}$ for *n*-butane at 293 K. Stretched exponential fit to the data is shown as a red line.

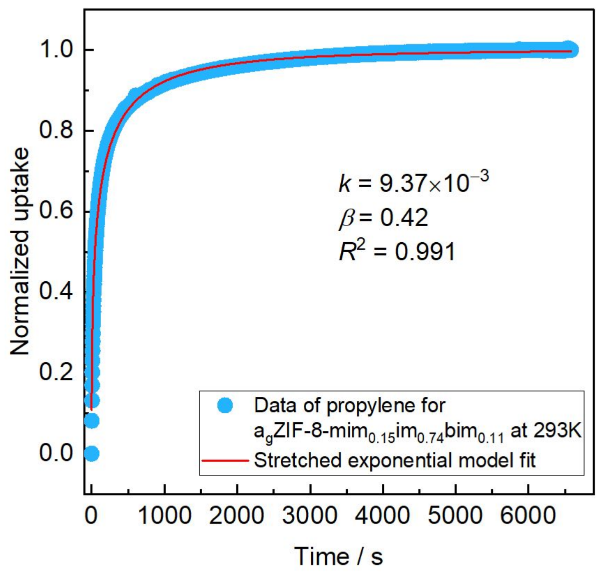

Supplementary Figure 108. Normalized uptake versus time for adsorption kinetic data of a_gZIF-8-$mim_{0.15}im_{0.74}bim_{0.11}$ for propylene at 293 K. Stretched exponential fit to the data is shown as a red line.

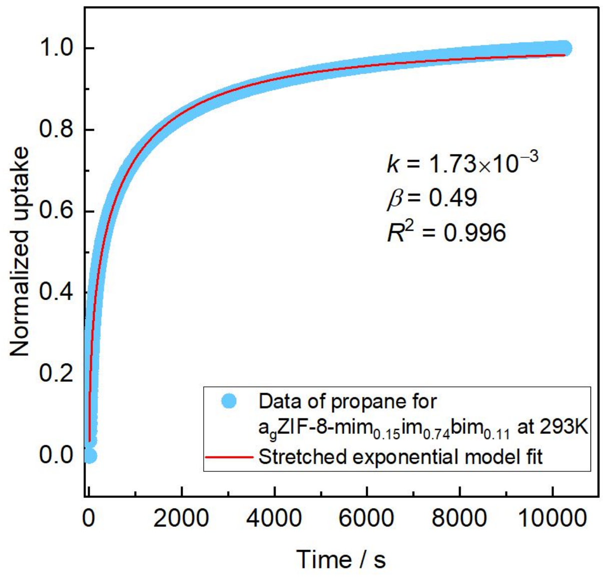

Supplementary Figure 109. Normalized uptake versus time for adsorption kinetic data of a_gZIF-8-$mim_{0.15}im_{0.74}bim_{0.11}$ for propane at 293 K. Stretched exponential fit to the data is shown as a red line.

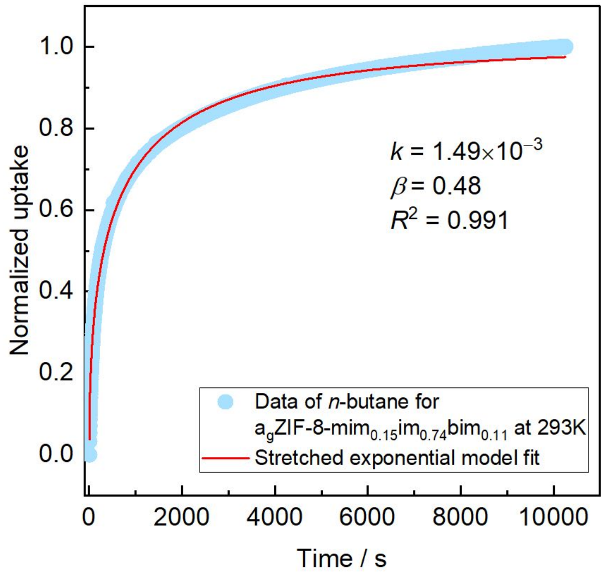

Supplementary Figure 110. Normalized uptake versus time for adsorption kinetic data of a_gZIF-8-mim$_{0.15}$im$_{0.74}$bim$_{0.11}$ for *n*-butane at 293 K. Stretched exponential fit to the data is shown as a red line.

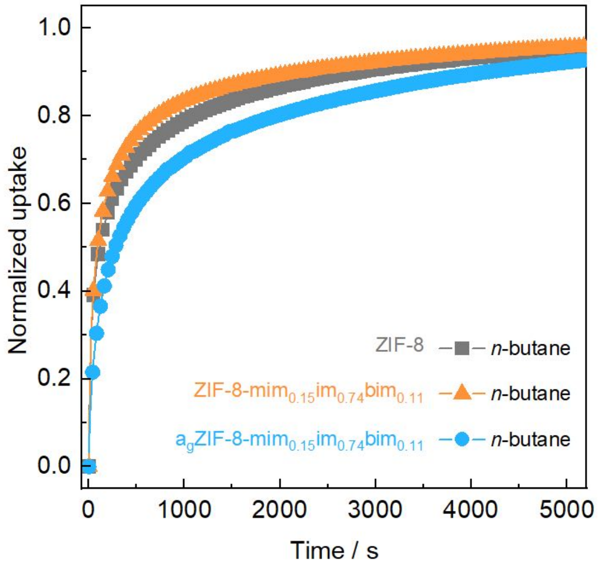

Supplementary Figure 111. Normalized uptake versus time for adsorption kinetic data of *n*-butane for ZIF-8, ZIF-8-mim$_{0.15}$im$_{0.74}$bim$_{0.11}$ (crystal) and a_gZIF-8-mim$_{0.15}$im$_{0.74}$bim$_{0.11}$ (glass) recorded at 293 K with an equilibrium pressure of about 80 kPa.

Supplementary References

1. Walba H, Isensee RW. Acidity constants of some arylimidazoles and their cations. *J. Org. Chem.* **26**, 2789-2791 (1961).
2. Du Y*, et al.* New high- and low-temperature phase changes of zif-7: elucidation and prediction of the thermodynamics of transitions. *J. Am. Chem. Soc.* **137**, 13603-13611 (2015).
3. Bennett TD*, et al.* Hybrid glasses from strong and fragile metal-organic framework liquids. *Nat. Commun.* **6**, 8079 (2015).
4. Song J*, et al.* Modulating liquid–liquid transitions and glass formation in zeolitic imidazolate frameworks by decoration with electron-withdrawing cyano groups. *J. Am. Chem. Soc.* **145**, 9273-9284 (2023).
5. Frentzel-Beyme L, Kolodzeiski P, Weiß J-B, Schneemann A, Henke S. Quantification of gas-accessible microporosity in metal-organic framework glasses. *Nat. Commun.* **13**, 7750 (2022).
6. Qiao A*, et al.* A metal-organic framework with ultrahigh glass-forming ability. *Sci. Adv.* **4**, eaao6827 (2018).
7. Morris W, Stevens CJ, Taylor RE, Dybowski C, Yaghi OM, Garcia-garibay MA. NMR and X-ray study revealing the rigidity of zeolitic imidazolate frameworks. *J. Phys. Chem. C* **116**, 13307-13312 (2012).
8. Zheng X*, et al.* Composite with a glassy nonporous coordination polymer enhances gas adsorption selectivity. *Inorg. Chem.* **62**, 1257-1263 (2023).
